# Solvent-controlled g-C_3_N_4_ visible-light photoredox benzylic C–H oxidation: selective synthesis of fluorenones and benzo[*c*]coumarins from 2-methyl-1,1′-biaryls

**DOI:** 10.1039/d6ra06292j

**Published:** 2026-08-24

**Authors:** Palani Natarajan, Pooja, Meena

**Affiliations:** a Department of Chemistry & Centre for Advanced Studies in Chemistry, Punjab University Chandigarh–160014 India pnataraj@pu.ac.in

## Abstract

Solvent-controlled reactivity, which enables the selective formation of distinct products from the same starting precursors by choosing the appropriate reaction media, is a crucial green chemistry approach. In fact, it reduces the need for additional reagents or precursors, restricts waste formation, and improves reaction efficiency and selectivity. Herein, using heterogeneous graphitic carbon nitride (g-C_3_N_4_) visible-light photoredox catalysis, we describe a novel solvent-regulated method to selectively synthesize a series of fluorenones (2) and benzo[*c*]coumarins (3) from a common precursor, 2-methyl-1,1′-biaryls (1), using dry CH_3_CN and CH_3_CN–water (9 : 1, v/v), respectively. Under anhydrous conditions, the reaction preferentially produces fluorenones (2) *via* intramolecular radical cyclization of *in situ* generated 2-phenylbenzyl radical intermediates followed by oxidation. In contrast, water and a surplus quantity of oxygen cause the chemical pathway to switch to oxidative lactonization, which results in the production of benzo[*c*]coumarins (3) with high selectivity. To the best of our knowledge, this is the first photocatalytic system that uses facile solvent control to produce two highly valuable heterocyclic frameworks from the same precursor.

## Abstract 2

Solvent-controlled g-C_3_N_4_ photocatalysis selectively converts 2-methyl-1,1′-biaryls into fluorenones in dry CH_3_CN or benzo[*c*]coumarins in aqueous CH_3_CN. Water and O_2_ switch the pathway from radical cyclization to an oxidative lactonization.

## Graphical abstract

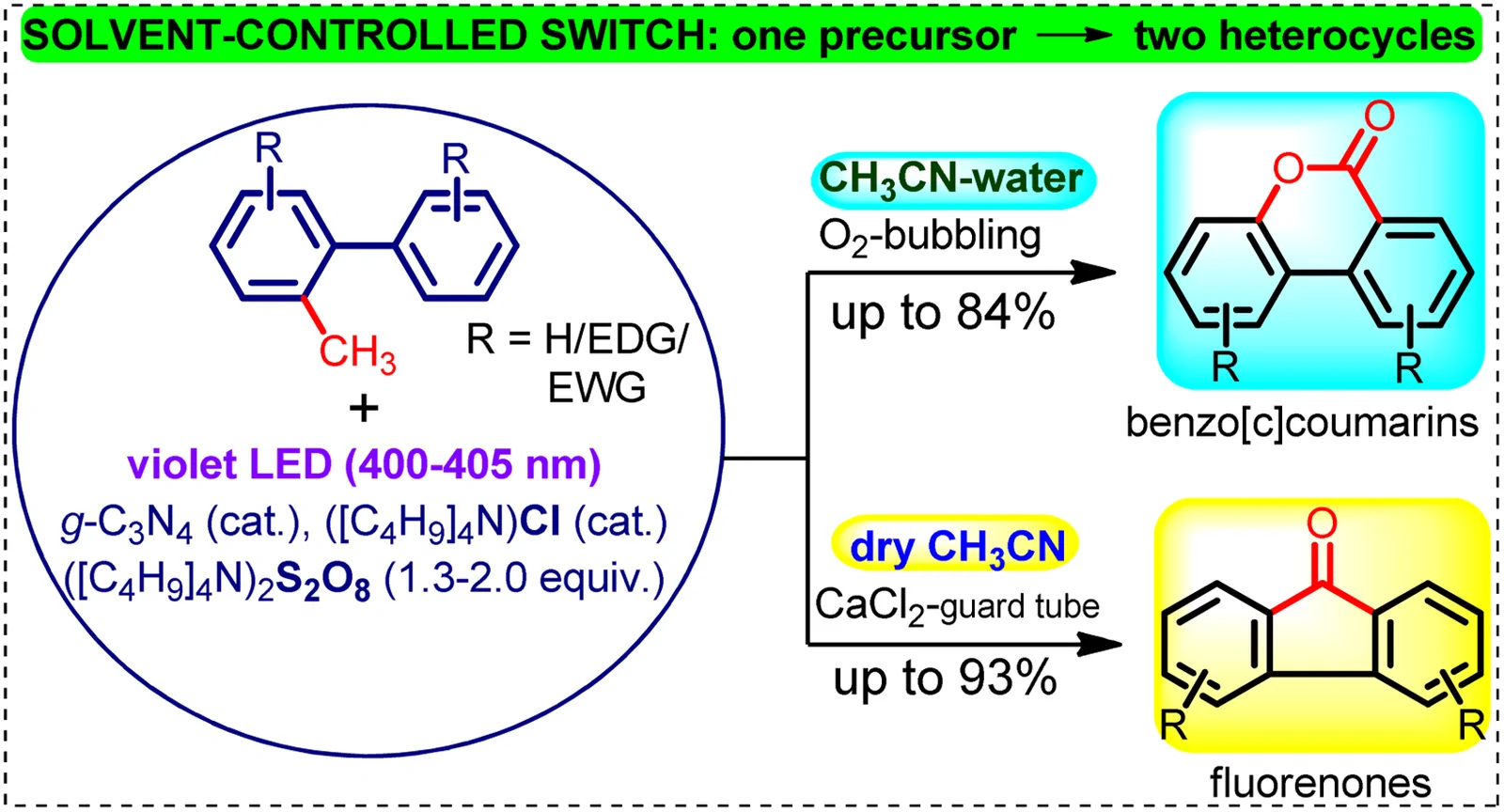

## 1 Introduction

Divergent synthesis has emerged as an attractive strategy in organic and medicinal chemistry because it enables the effective formation of structurally distinct molecular frameworks from the same starting precursors.^1^ In light of this advantage, a handful of switchable transformations based on divergent reaction pathways have been reported over the past two decades.^2^ While it is still relatively new and challenging, controlling reaction outcomes through solvent effects is one of various ways that is potentially easier to adopt and more environmentally friendly.^3^ Therefore, the development of solvent-dependent divergent methodologies for the synthesis of structurally diverse heterocycles is very desirable.^4^

**Fig. 1:**
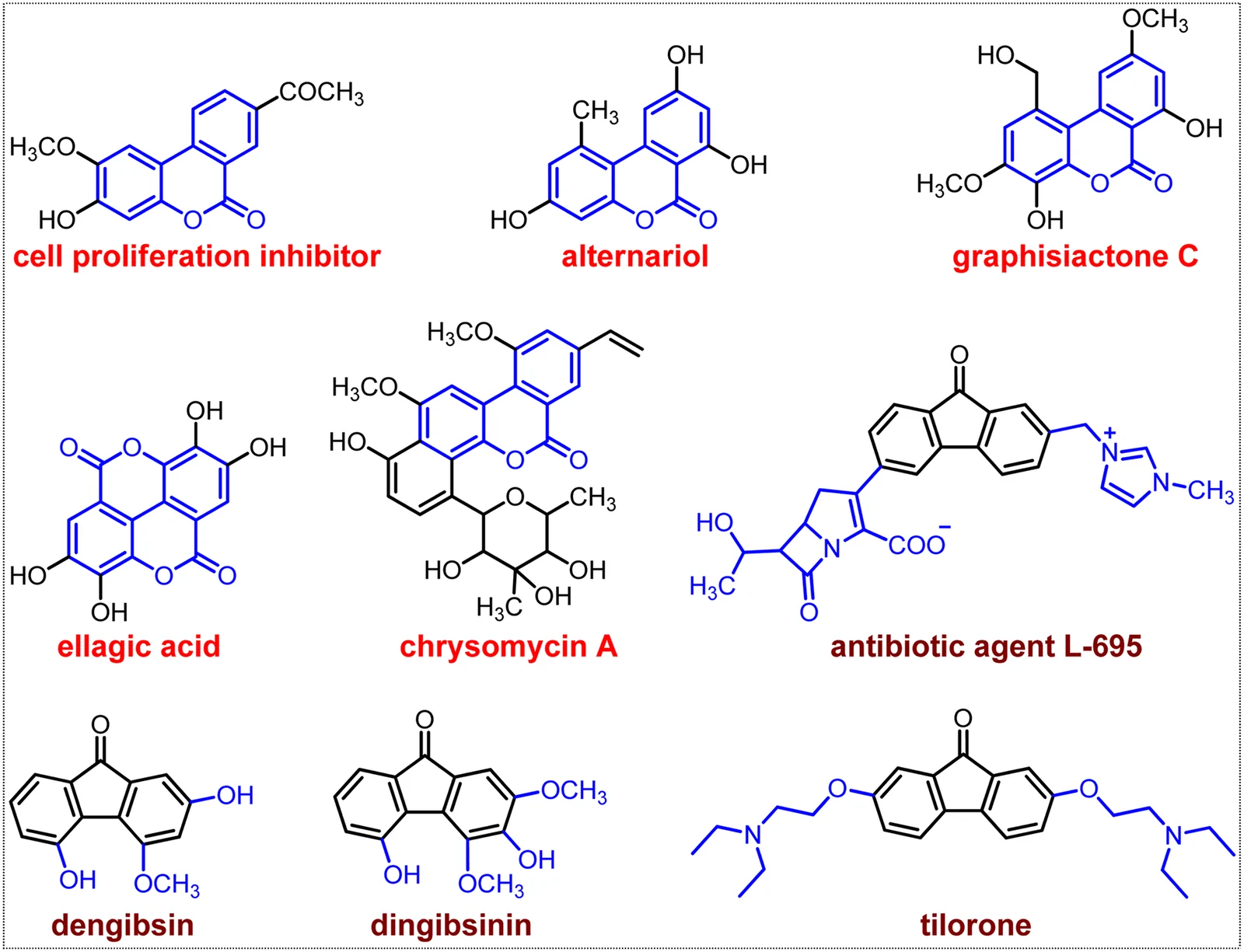
Few biologically active and important molecules containing fluorenones (2) and benzo[*c*]coumarins (3) frameworks in their structure.

Fluorenones and benzo[*c*]coumarins are two important classes of oxygen-containing polycyclic frameworks that are commonly found in biologically active molecules,^5^ functional materials,^6^ and natural products (Fig. 1).^7^ Owing to their extended π-conjugated systems, derivatives of these scaffolds have attracted a lot of interest in materials science, particularly for use in molecular sensing,^8^ organic electrical devices,^9^ liquid-crystal displays,^10^ molecular wires,^11^ medicines,^12^ and agrochemicals (Fig. 1).^13^ As a result, substantial efforts have been made to develop efficient synthetic strategies for their preparation. For example, benzo[*c*]coumarins are usually made by lactonization of functionalized biaryl carboxylic acids,^14,15^ oxidative C–H lactonization of 2-arylbenzaldehydes,^16^ and related oxidative annulation processes,^17^ or Baeyer–Villiger oxidation of cyclic-ketones,^18^ whereas fluorenones are commonly synthesized by intramolecular cyclization of biaryl precursors,^19^ oxidative transformations of fluorene derivatives,^20^ or transition-metal-catalyzed annulation reactions.^21^ Despite their significance, most of these methods frequently depend on homogeneous (metal) catalysts and hazardous substrates/reagents, which often limit their sustainability and use. Thus, from the perspective of industrial applicability and green chemistry,^22^ it would be highly desirable to develop a heterogeneous-catalyzed^23^ and solvent-controlled divergent strategy that allows selective access to both fluorenone and benzo[*c*]coumarin frameworks from common starting precursors.

**Scheme 1:**
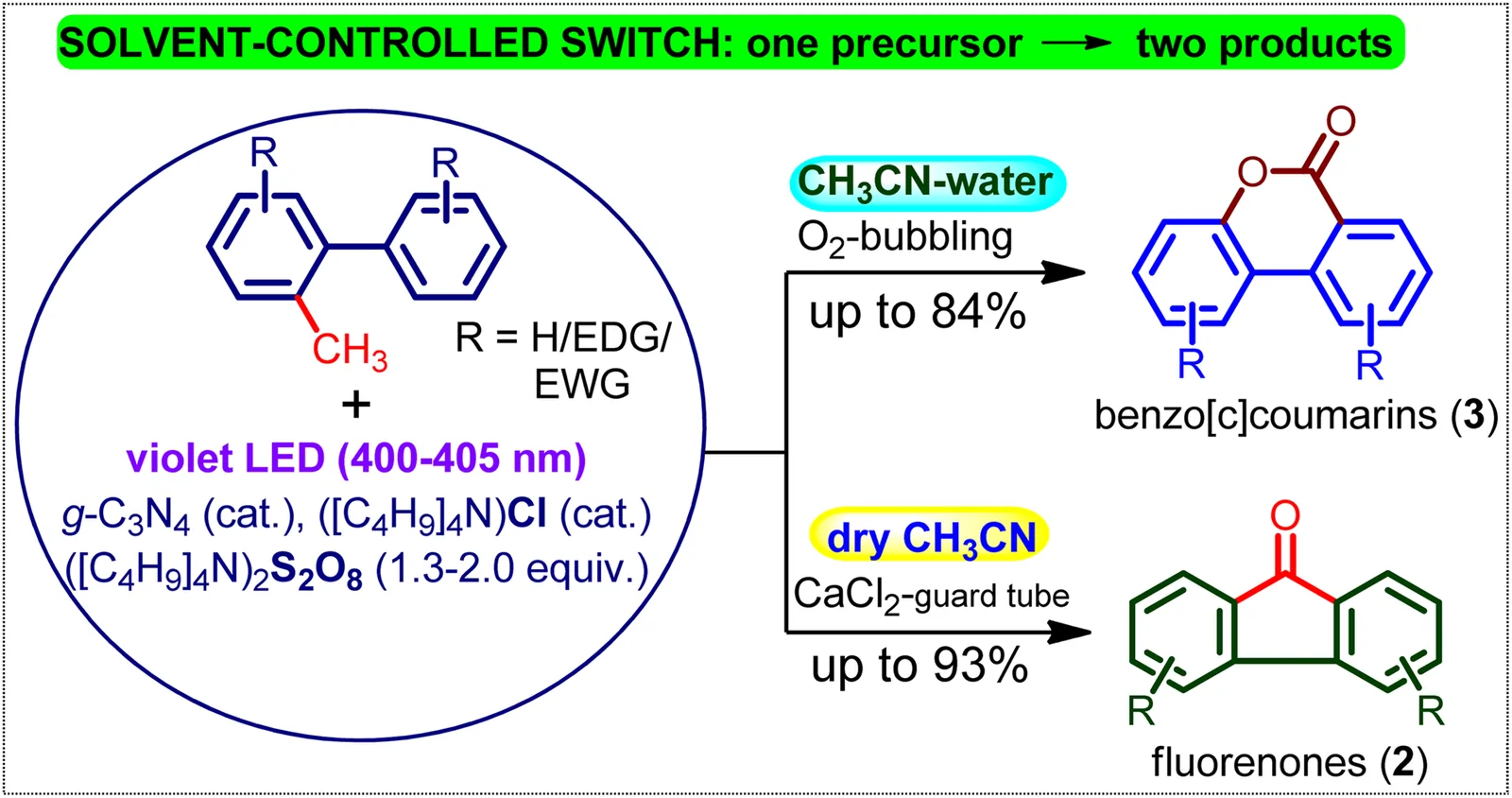
A novel solvent-regulated method to selectively synthesize a series of fluorenones (2) and benzo[*c*]coumarins (3) from a common precursor, 2-methyl-1,1′-biaryls (1) herein reported.

Persulfate (S_2_O_8_^2−^), commonly known as peroxydisulfate or peroxodisulfate, is a cheap crystalline oxidant that is stable under ambient conditions and easy to handle and store.^24^ Persulfate can produce highly reactive sulfate radical anions (SO_4_˙^−^, *E* = 2.5–3.1 V *vs.* NHE) upon activation, which function as potent one-electron oxidants.^25^ Such activation has frequently been achieved by heat treatment, metal ions, UV radiation, or visible-light photocatalysis with nitrogen-doped carbon material.^26^ Among these methods, the use of visible-light photocatalysis with nitrogen-doped carbon material—specifically, g-C_3_N_4_ visible-light photocatalysis—to activate persulfate has garnered more interest in recent years.^26,27^ The g-C_3_N_4_ offers several advantages, including excellent stability under acidic and basic environments, high availability, low toxicity, large surface areas, and the practical benefits of easy separation and recyclability.^27^ Consequently, in addition to environmental applications, the g-C_3_N_4_ visible-light photocatalytic system has proven valuable in organic synthesis.^28^ Recently, our group demonstrated the synthesis of several heterocyclic frameworks, including 3-nitro-4-aryl-2*H*-chromen-2-ones,^29^ 2*H*-benzo[*b*][1,4]oxazin-2-ones,^30^ and methyl(iso)quinolones^31^ utilizing g-C_3_N_4_ visible-light photocatalysis. In continuation, we herein report a solvent-regulated strategy for the selective synthesis of fluorenones (2) and benzo[*c*]coumarins (3) from a common precursor, 2-methyl-1,1′-biaryls (1, Scheme 1). It is interesting to note that the reaction's outcome can be readily controlled by altering the reaction medium: dry CH_3_CN affords fluorenones, whereas CH_3_CN–water (9 : 1, v/v) leads to benzo[*c*]coumarins in good yields.

## 2 Results and discussion

In continuation of our previous works on persulfate and g-C_3_N_4_-catalyzed visible-light photoredox catalysis,^29–31^ optimization studies were initiated using commercially available 2-methylbiphenyl (1a, 101 mg, 0.6 mmol) as the model substrate, g-C_3_N_4_ (5 mg) as photocatalyst, Na_2_S_2_O_8_ (143 mg, 0.6 mmol, 1.0 equiv.) as the source of SO_4_˙^−^ radicals, water (2 mL) as the solvent, and a blue LED (12 W, *λ* = 455 ± 10 nm) as the irradiation source (Table 1, entry 1). However, no product was observed even after 24 h of stirring at 25 °C under open-air atmosphere, most likely as a result of substrate 1a's poor water solubility. In order to resolve this problem, the reaction was repeated in dry CH_3_CN under a CaCl_2_ guard tube atmosphere. Thankfully, fluorenone (2a) was obtained in 7% yield (Table 1, entry 2). The product 2a was isolated and characterized by NMR and mass analysis, which were consistent with literature reports (ESI). The impact of the irradiation source was then investigated. The reaction efficiency was increased by adopting a violet LED (5 W, *λ* = 400–405 nm, luminous flux: 741 mW) in place of the blue LED, resulting in 2a with a 13% yield (Table 1, entry 5). Importantly, no product was observed in the absence of an irradiation source (Table 1, entry 7). Next, the impact of the solvent was studied (Table 1, entries 8–16). Low yields of 2a were obtained using common organic solvents like dry dichloromethane, dry ethyl acetate, dry ethanol, dry acetone, and dry DMF, as well as mixed solvent systems including CH_3_CN–water (19 : 1, v/v), ethanol–water (19 : 1, v/v), DMF–water (19 : 1, v/v), and acetone–water (19 : 1, v/v, Table 1, entries 8–16). As a result, acetonitrile was selected as the most suitable solvent for 2a formation (Table 1, entry 5). It is interesting to note that the product distribution altered substantially when a CH_3_CN–H_2_O mixture (19 : 1, v/v) was utilized as a medium (Table 1, entry 13). Benzo[*c*]coumarin (3a) was also found in addition to fluorenone (2a), indicating that the aqueous medium has a significant impact on the reaction pathway (Table 1, entry 13). To boost 2a yield, we looked into adding chloride (Cl^−^) salts in the reaction, which are known to facilitate benzylic C–H activation through hydrogen abstraction by chlorine radicals.^32^ As expected, tetrabutylammonium chloride (TBACl, 5 mol%) markedly raised the yield of 2a to 30% in dry acetonitrile (Table 1, entry 19). Other chloride sources, including LiCl, HCl, and NaCl, were less efficient (Table 1, entries 17–18 and 20), highlighting the importance of the tetrabutylammonium counterion, possibly due to its non-deliquescent nature and superior solubility in organic solvents.^32^ Afterwards, TBACl loading was optimized (Table 1, entries 21–23). Conversion decreased at lower chloride loadings (Table 1, entry 21). Increasing the TBACl loading from 5 to 10 mol% improved the yield further (Table 1, entry 22), whereas higher concentrations led to a slight decrease in 2a yield (Table 1, entry 23). After that, a variety of persulfate oxidants were assessed, such as K_2_S_2_O_8_, (NH_4_)_2_S_2_O_8_, Li_2_S_2_O_8_, and tetrabutylammonium persulfate ([C_4_H_4_]_4_N)_2_S_2_O_8_, TBAPS). TBAPS produced the greatest outcomes among them (Table 1, entries 24–27). Subsequent optimization revealed that the yield of 2a increased to 76% when the quantity of TBAPS was raised from 1.0 to 1.3 equivalents (Table 1, entry 28). Similarly, increasing the amount of g-C_3_N_4_ from 5 to 8 mg resulted in a modest increase in product yield (Table 1, entry 30). On the other hand, in the absence of g-C_3_N_4_, no product was detected, confirming its crucial role in the reaction (Table 1, entry 32). Additionally, we assessed a number of traditional heterogeneous photoredox catalysts, such as titanium dioxide (TiO_2_, band gap 3.2 eV), bismuth oxide (Bi_2_O_3_, band gap 2.8 eV), cadmium sulfide (CdS, band gap 2.4 eV), and cadmium selenide (CdSe, band gap 1.8 eV). None of these catalysts perform better than g-C_3_N_4_. The superior activity of g-C_3_N_4_ cannot be attributed solely to catalyst stability. It likely arises from a combination of band-edge energies, light absorption, interfacial charge transfer, and catalyst stability.^26,27^ Also, the yield of 2a was negligible when the reaction was carried out under a nitrogen gas environment (Table 1, entry 33). Therefore, the condition described in Table 1, entry 30, was selected as optimal, which afforded fluorenone (2a) in 92% yield.

**Table 1:** Selected results of screening the optimal reaction conditions to access fluorenones (2) and benzo[*c*]coumarins (3) from 2-methyl-1,1′-biaryls (1)^a^

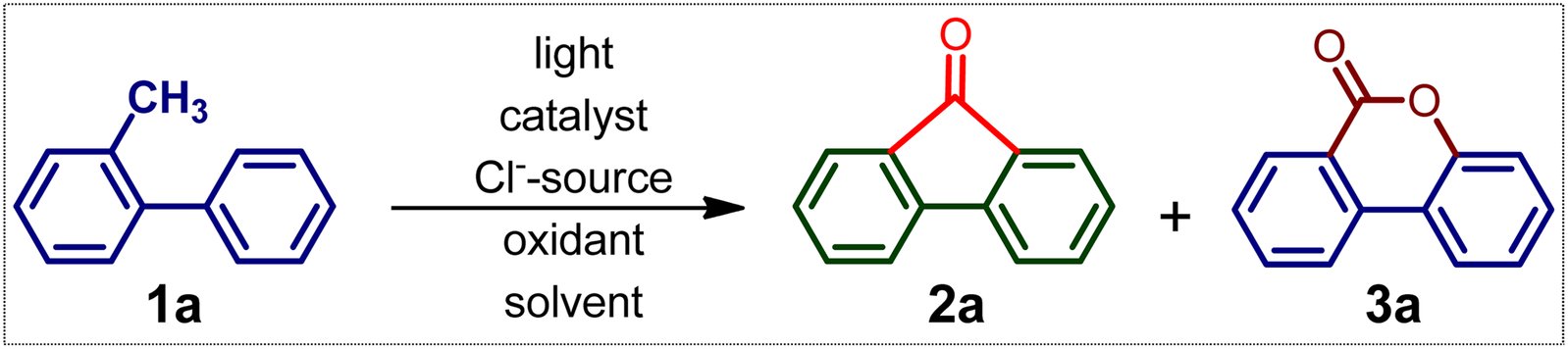							
Entry	Solvent (mL)^b^	Persulfate (equiv.)^c^	Cl^−^-source (mol%)^c^	g-C_3_N_4_ (mg)^c^	Time (h)	Yield of 2a (%)^d^	Yield of 3a (%)^e^
1	Water (2)	Na_2_S_2_O_8_ (1.0)	—	5	24	ND^f^	ND
2	CH_3_CN (2)	Na_2_S_2_O_8_ (1.0)	—	5	24	7^f^	ND
3	CH_3_CN (2)	Na_2_S_2_O_8_ (1.0)	—	5	24	ND^g^	ND
4	CH_3_CN (2)	Na_2_S_2_O_8_ (1.0)	—	5	24	<5^h^	ND
5	CH_3_CN (2)	Na_2_S_2_O_8_ (1.0)	—	5	24	13	ND
6	CH_3_CN (2)	Na_2_S_2_O_8_ (1.0)	—	5	24	6^i^	ND
7	CH_3_CN (2)	Na_2_S_2_O_8_ (1.0)	—	5	24	ND^j^	ND
8	CH_2_Cl_2_ (2)	Na_2_S_2_O_8_ (1.0)	—	5	24	ND	ND
9	Ethyl acetate (2)	Na_2_S_2_O_8_ (1.0)	—	5	24	ND	ND
10	Ethanol (2)	Na_2_S_2_O_8_ (1.0)	—	5	24	<5	ND
11	Acetone (2)	Na_2_S_2_O_8_ (1.0)	—	5	24	ND	ND
12	DMF (2)	Na_2_S_2_O_8_ (1.0)	—	5	24	<10	ND
13	CH_3_CN–H_2_O (19 : 1, v/v, 2)	Na_2_S_2_O_8_ (1.0)	—	5	24	6	8
14	Ethanol–H_2_O (19 : 1, v/v, 2)	Na_2_S_2_O_8_ (1.0)	—	5	24	ND	Trace
15	DMF–H_2_O (19 : 1, v/v, 2)	Na_2_S_2_O_8_ (1.0)	—	5	24	Trace	ND
16	Acetone–H_2_O (19 : 1, v/v, 2)	Na_2_S_2_O_8_ (1.0)	—	5	24	ND	ND
17	CH_3_CN (2)	Na_2_S_2_O_8_ (1.0)	LiCl (5)	5	24	21	ND
18	CH_3_CN (2)	Na_2_S_2_O_8_ (1.0)	HCl (5)	5	24	<10	11
19	CH_3_CN (2)	Na_2_S_2_O_8_ (1.0)	TBACl (5)	5	24	30	ND
20	CH_3_CN (2)	Na_2_S_2_O_8_ (1.0)	NaCl (5)	5	24	16	ND
21	CH_3_CN (2)	Na_2_S_2_O_8_ (1.0)	TBACl (3)	5	24	22	ND
22	CH_3_CN (2)	Na_2_S_2_O_8_ (1.0)	TBACl (10)	5	24	52	Trace
23	CH_3_CN (2)	Na_2_S_2_O_8_ (1.0)	TBACl (20)	5	24	49	Trace
24	CH_3_CN (2)	K_2_S_2_O_8_ (1.0)	TBACl (10)	5	24	23	ND
25	CH_3_CN (2)	(NH_4_)_2_S_2_O_8_ (1.0)	TBACl (10)	5	24	Trace	ND
26	CH_3_CN (2)	Li_2_S_2_O_8_ (1.0)	TBACl (10)	5	24	48	Trace
27	CH_3_CN (2)	TBAPS (1)	TBACl (10)	5	24	63	ND
28	CH_3_CN (2)	TBAPS (1.3)	TBACl (10)	5	22	76	ND
29	CH_3_CN (2)	TBAPS (1.5)	TBACl (10)	5	22	75	ND
**30**	**CH**_**3**_ **CN (2)**	**TBAPS (1.3)**	**TBACl (10)**	**8**	**21**	**92** k	**ND**
31	CH_3_CN (2)	TBAPS (1.3)	TBACl (10)	10	21	93	ND
32	CH_3_CN (2)	TBAPS (1.3)	TBACl (10)	0	21	Trace	ND
33	CH_3_CN (2)	TBAPS (1.3)	TBACl (10)	8	21	ND^l^^,^^m^	ND
34	CH_3_CN–H_2_O (19 : 1, v/v, 2)	TBAPS (1.3)	TBACl (10)	8	24	16	23
35	CH_3_CN–H_2_O (19.9 : 0.1, v/v, 2)	TBAPS (1.3)	TBACl (10)	8	24	41	7
36	CH_3_CN–H_2_O (9 : 1, v/v, 2)	TBAPS (1.3)	TBACl (10)	8	24	Trace	40
37	CH_3_CN–H_2_O (4 : 1, v/v, 2)	TBAPS (1.3)	TBACl (10)	8	24	ND	18
38	CH_3_CN–H_2_O (9 : 1, v/v, 2)	TBAPS (1.3)	TBACl (10)	8	24	ND	46^n^
39	CH_3_CN–H_2_O (9 : 1, v/v, 2)	TBAPS (1.3)	TBACl (10)	8	24	ND	57^o^^,^^p^
**40**	**CH**_**3**_ **CN–H**_**2**_ **O (9:1, v/v,2)**	**TBAPS (2.0)**	**TBACl (10)**	**8**	**24**	**ND**	**84** k ^,^ o
41	CH_3_CN–H_2_O (9 : 1, v/v, 2)	TBAPS (2.2)	TBACl (10)	8	24	ND	85^o^
42	CH_3_CN–H_2_O (9 : 1, v/v, 2)	TBAPS (3.0)	TBACl (10)	8	24	ND	87^o^
43	CH_3_CN (2)	TBAPS (1.3)	TBAB (10)	8	21	25	ND
44	CH_3_CN (2)	TBAPS (1.3)	TBAOAc (10)	8	21	18^q^	ND

a Unless stated otherwise, all reactions were performed in a vial equipped with 2-methylbiphenyl (1a, 101 mg, 0.6 mmol), SO_4_^−^˙-source, Cl˙-source, and g-C_3_N_4_ catalyst in solvent (2 mL) and irradiation using a violet LED (5 W, *λ* = 400–405 nm, luminous flux: 741 mW) under oxygen atmosphere at 25 °C.

b Solvents were distilled before use.

c Commercially available high purity catalysts, Cl˙-source and SO_4_^−^˙-source were purchased and utilized as received.

d Unless stated otherwise, reactions were performed in dry CH_3_CN under a CaCl_2_ guard tube atmosphere and yield determined by GC analysis.

e Unless stated otherwise, reactions were performed in CH_3_CN–H_2_O (9 : 1, v/v) under oxygen bubbling atmosphere and yield determined by GC analysis.

f Done with blue LEDs.

g Done with green LEDs.

h Done with yellow LEDs.

i Done with white LEDs.

j Done in the dark.

k Isolated yield.

l Done under a N_2_ gas atmosphere.

m About 90% of 9*H*-fluorene (4) was isolated.

n Done under O_2_-balloon atmosphere.

o Done under O_2_-gas bubbling atmosphere.

p About 10% of biphenyl-2-carboxaldehyde (5) was isolated.

q About 5–6% of methylated-biphenyl and methylated-fluorenone were detected. TBAPS: tetrabutylammonium persulfate ([C_4_H_4_]_4_N)_2_S_2_O_8_. TBACl: tetrabutylammonium chloride. TBABr: tetrabutylammonium bromide. TBAOAc: tetrabutylammonium acetate. ND: not detected.

As mentioned earlier, benzo[*c*]coumarins (3), in addition to fluorenones (2), represent an important class of compounds frequently encountered in biologically active molecules, functional materials, and natural products (Fig. 1).^13^ Encouraged by the preliminary observation of formation of benzo[*c*]coumarin (3a) in aqueous conditions (Table 1, entries 13 and 18), we next focused on identifying optimal conditions for the selective synthesis of benzo[*c*]coumarins (3) from 2-methyl-1,1′-biaryls (1). Accordingly, the reaction of 2-methylbiphenyl (1a, 101 mg, 0.6 mmol) with g-C_3_N_4_ (8 mg), TBACl (17 mg, 10 mol%), and TBAPS (528 mg, 1.3 equiv.) in CH_3_CN–H_2_O (19 : 1, v/v, 2 mL) under open-air atmosphere afforded the desired benzo[*c*]coumarin (3a) in 23% yield (Table 1, entry 34). The product 3a was isolated and characterized by NMR and mass measurements (ESI). Afterwards, the water ratio in CH_3_CN was optimized (Table 1, entries 35–37). Lower water content in CH_3_CN (19.9 : 0.1, v/v) resulted in diminished conversion to 3a, highlighting the critical role of water in the reaction pathway (Table 1, entry 35). Increasing the water content from 5% to 10% significantly improved the yield of 3a (Table 1, entry 36), but much higher water concentration (20% water in acetonitrile) led to a drop in product yield (Table 1, entry 37). These results explicitly show that simple modulation of the solvent system enables a solvent-controlled switch in the reaction's outcome, thereby giving selective access to two valuable heterocyclic frameworks from the same precursor. To further improve the efficiency of benzo[*c*]coumarin formation, the effect of oxygen concentration was investigated (Table 1, entries 38–39). The yield of 3a rose to 46% when an O_2_ balloon was used in place of the open-air atmosphere (Table 1, entry 38). Further enhancement was achieved under continuous O_2_-bubbling conditions, furnishing benzo[*c*]coumarin (3a) in 57% yield (Table 1, entry 39). Subsequent optimization revealed that the yield of 3a rose to 84% when the quantity of TBAPS was increased from 1.3 to 2.0 equivalents (Table 1, entry 40). Further increases in TBAPS loading did not improve the yield (Table 1, entries 41–42). As a consequence, the conditions described in Table 1, entry 30, which afforded fluorenone (2a) in 92% yield, and those in Table 1, entry 40, which produced benzo[*c*]coumarin (3a) in 84% yield, were identified as the optimal conditions for the selective synthesis of the respective products. Under these optimized reaction conditions (Table 1, entry 30 and entry 40), the scalability was also examined. When the reactions were conducted on a 10 mmol scale of 1a, the isolated yield of 2a and 3a, respectively, decreased somewhat to 71% and 57% yields. The use of flow techniques is a well-established approach to solve the scale-up problems in this type of reactions.^33^

After optimization of the reaction conditions, the substrate scope under the two optimized catalytic modes such as Table 1, entry 30 for selective synthesis of fluorenones (2) and Table 1, entry 40 for selective synthesis of benzo[*c*]coumarins (3) was systematically examined. We first investigated the synthesis of fluorenones (2) from 2-methyl-1,1′-biaryls (1) using the optimized condition given in Table 1, entry 30. As seen in Table 2, a broad range of functionalized 2-methyl-1,1′-biaryls (1) underwent smooth oxidative cyclization to furnish the corresponding fluorenones (2) in good yields. Substrates having electron-donating groups such as *tert*-butyl, phenyl, phenoxy, and trifluoromethoxy substituents on the tolyl-ring were well tolerated, and the desired products 2b–2c and 2e–2f were produced in 88–93% yields. Polycyclic substrates also participated efficiently under the optimized condition to deliver fused fluorenones 2d and 2s in high yields. However, substrates containing electron-withdrawing groups like trifluoromethyl, cyano and ester substituents provided the corresponding products 2g–2i in moderate yields (Table 2). This reduction in efficiency is most likely due to the more difficult oxidation of the benzylic methyl group in electron-deficient tolyl-ring.^19–21^ Halogen-substituted substrates, particularly chloro- and fluoro-substituted biaryls, were found to be well compatible to the optimized reaction condition, furnishing the desired fluorenones 2k–2l, 2n, and 2p in good yields (Table 2). In contrast, the bromo-substituted substrate failed to generate the expected product 2j; instead, the corresponding *ipso*-chlorinated product 2k was obtained in 54% yield. The C–Br bond's low stability and ease of cleavage under reaction condition may account for this outcome.^34^ Similarly, the nitro-substituted substrate did not undergo the desired cyclization, and product 2m was undetectable. This behaviour is likely due to the strongly electron-withdrawing nature of the nitro group, which significantly disfavours oxidation of the benzylic methyl group.^20^ The scope was further extended to *meta*- and *ortho*-substituted biaryls and substituent on the non-tolyl unit of 2-methyl-1,1′-biaryls (1). In fact, they underwent cyclization smoothly to furnish the corresponding fluorenones 2n–2r in good yields (Table 2). The unsymmetrical polycyclic substrate was notably annulated with low regioselectivity, resulting in an inseparable combination of regioisomers 2s and 2s′ in a ratio of 1 : 0.3. This finding is in line with the behaviour frequently seen in radical-mediated aromatic cyclizations, where regioselective control is often limited.^29–31^

**Table 2:** Synthesis of fluorenones (2) from 2-methyl-1,1′-biaryls (1) using the optimized condition given in Table 1, entry 30^a^

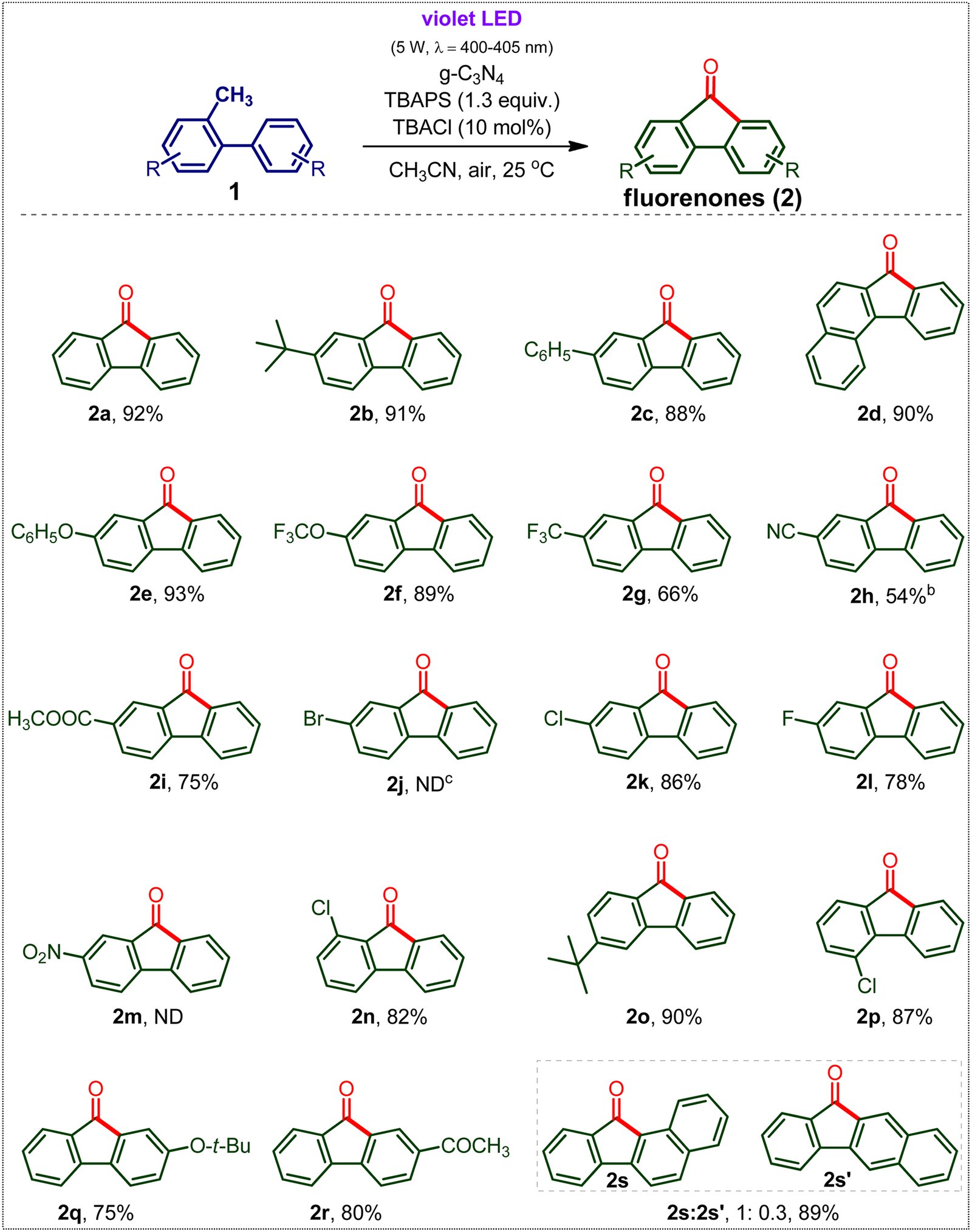

a Unless stated otherwise, all reactions were performed in a vial equipped with 2-methyl-1,1′-biaryls (1, 1.0 mmol, 1.0 equiv.), *g*-C_3_N_4_ (13.5 mg), TBAPS (1.3 mmol, 1.3 equiv.), TBACl (10 mol%, 0.1 equiv.), and dry CH_3_CN (4 mL) and were irradiation using a violet LED (5 W, *λ* = 400–405 nm, luminous flux: 741 mW) under oxygen atmosphere through CaCl_2_ guard tube at 25 °C for 21 h. Isolated yields are mentioned.

b Also, some amount of 9*H*-fluorene-2-carbonitrile was observed.

c Desired product 2j not detected, however, 2-chloro-9*H*-fluoren-9-one was detected in 54%.

We then investigated the selective synthesis of benzo[*c*]coumarins (3) from 2-methyl-1,1′-biaryls (1) using optimized condition given in Table 1, entry 40. Table 3 shows that a variety of substituted 2-methyl-1,1′-biaryls (1) underwent smooth oxidative lactonization and cyclization to afford the corresponding benzo[*c*]coumarins (3) in good yields. Substrates having electron-donating substituents on the tolyl ring, such as *tert*-butyl, phenyl, naphthyl, and phenoxy, were well tolerated in the transformation to provide the desired products 3a–3e in 73–84% yields. Similarly, the electron-deficient substituents on the tolyl-framework had no discernible effect on the cyclization efficiency. Substrates with –CF_3_ and chloro groups furnished the corresponding benzo[*c*]coumarins 3f–3h in 63–85% yields (Table 3). Furthermore, substrate bearing substituents such as phenyl, phenoxy, trifluoromethoxy, and chloro on the non-tolyl unit of 2-methyl-1,1′-biaryls (1) yielded the corresponding benzo[*c*]coumarins 3i–3l in good yields. Thus, the results shown in Tables 2 and 3 demonstrate the considerable potential of this solvent-controlled approach for the divergent synthesis of functionalized fluorenones (2) and benzo[*c*]coumarins (3) in good yields. In other words, with proper solvent management, two distinct classes of valuable heterocyclic frameworks can be selectively synthesized from the same starting materials (such as 2-methyl-1,1′-biaryls) under otherwise almost identical reaction conditions.

**Table 3:** Synthesis of benzo[*c*]coumarins (3) from 2-methyl-1,1′-biaryls (1) using the optimized condition given in Table 1, entry 40^a^

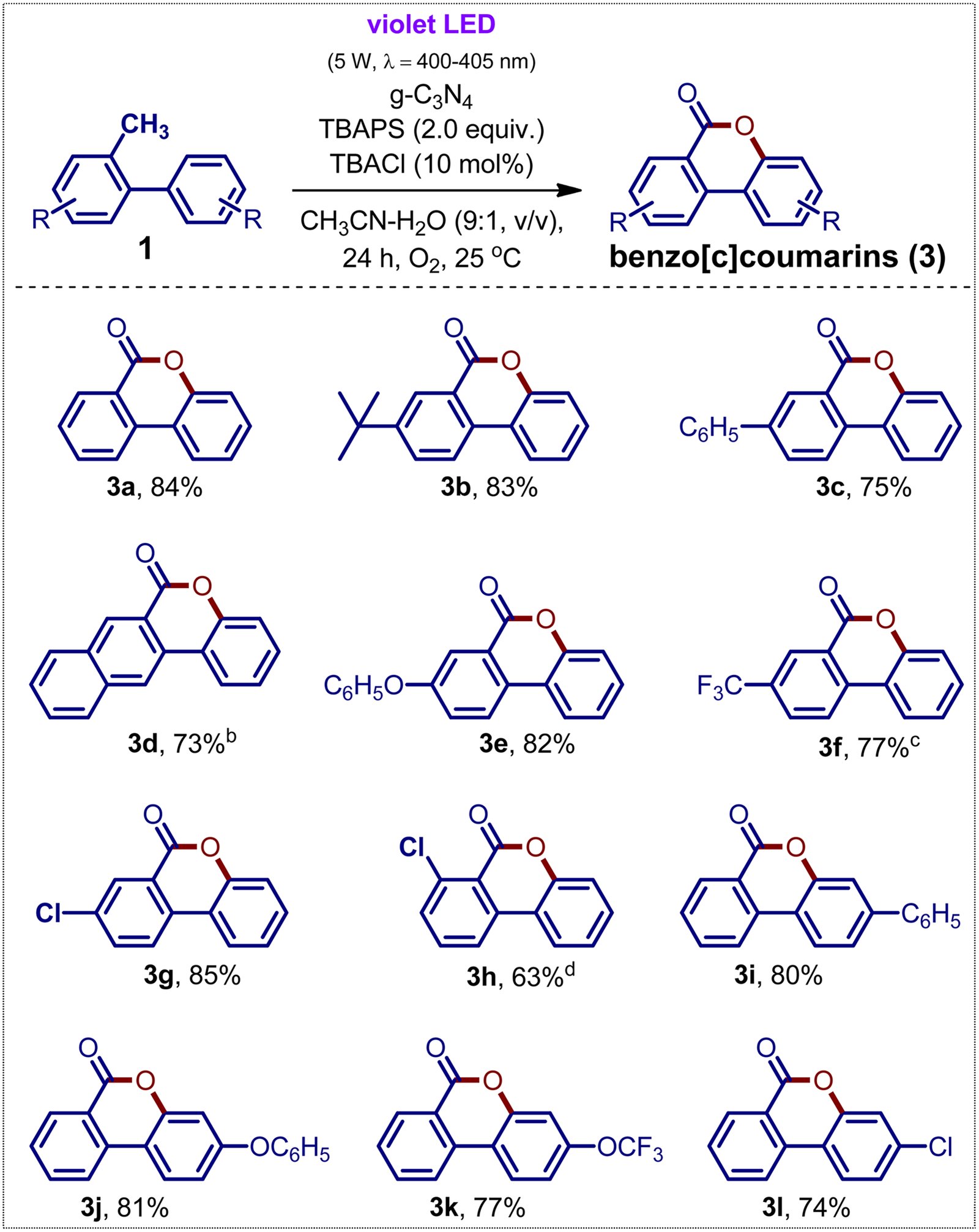

a Unless stated otherwise, all reactions were performed in a vial equipped with 2-methyl-1,1′-biaryls (1, 1.0 mmol, 1.0 equiv.), *g*-C_3_N_4_ (13.5 mg), TBAPS (2.0 mmol, 2.0 equiv.), TBACl (10 mol%, 0.1 equiv.), and CH_3_CN–water (9 : 1, v/v, 4 mL) and were irradiation using a violet LED (5 W, *λ* = 400–405 nm, luminous flux: 741 mW) under oxygen bubbling atmosphere at 25 °C for 24 h. Isolated yields are mentioned.

b Also 3-phenyl-2-naphthoic acid (5%) was observed.

c Also some amount of 4-(trifluoromethyl)–[1,1′-biphenyl]-2-carboxylic acid was observed.

d Also some amount of (3-chloro-[1,1′-biphenyl]-2 yl)methanol was observed.

**Fig. 2:**
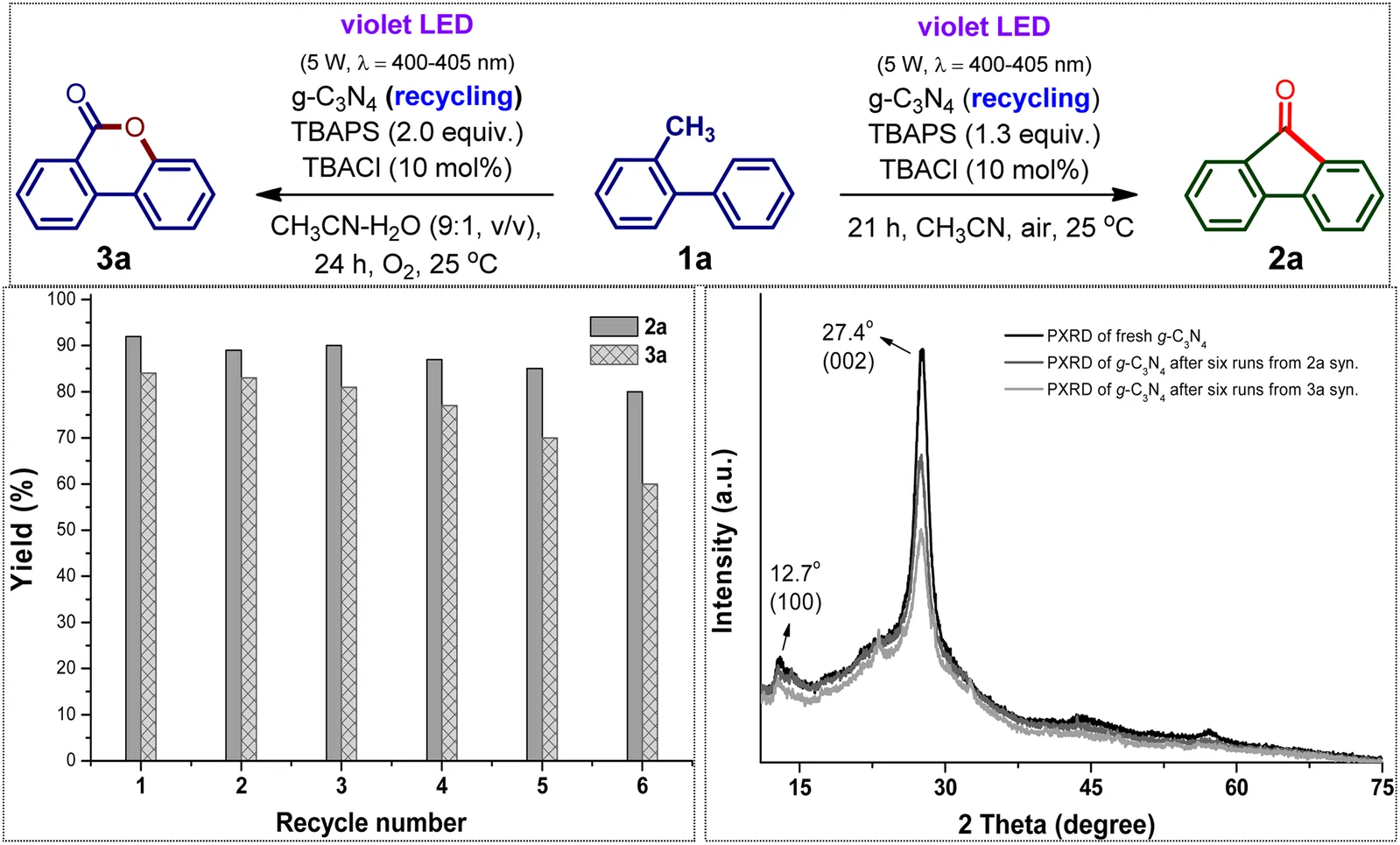
An evaluation of the recyclability of g-C_3_N_4_.

Next, to evaluate the recyclability of the photocatalyst, after completion of the reaction, g-C_3_N_4_ was recovered by centrifugation, thoroughly washed with ethyl acetate, acetonitrile, water, and again acetonitrile, and then reused in subsequent runs. This extensive washing procedure was intended to remove residual substrates, products, and other weakly or moderately adsorbed species. As shown in Fig. 2, g-C_3_N_4_ retained its high photocatalytic activity in both the conversion of 2-methylbiphenyl (1a) to fluorenone (2a) and 1a to benzo[*c*]coumarin (3a) for multiple cycles. These results clearly demonstrate the robustness and recyclability of g-C_3_N_4_ as an efficient heterogeneous photocatalyst under the reaction conditions.^27^ Nevertheless, the minor accumulation of strongly adsorbed species cannot be completely excluded.^29–31^

**Fig. 3:**
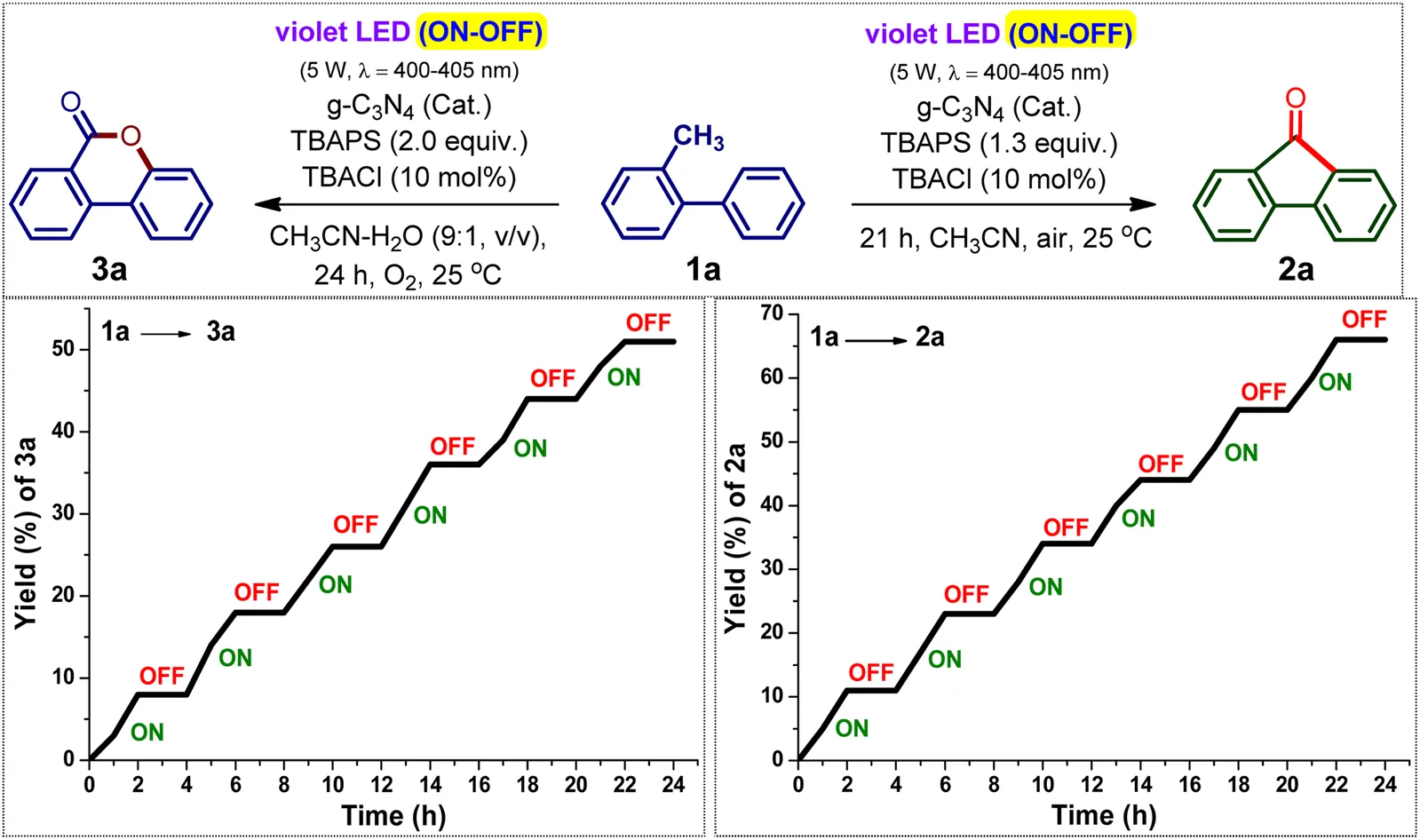
Light on-off experiments for the synthesis of 2a and 3a from 1a under the optimized reaction conditions.

Next, to probe the role of light in the reaction, a light on/off experiment was performed under the optimized reaction conditions (Fig. 3). Upon irradiation, the reaction proceeded smoothly; however, when the light source was switched off, the reaction ceased immediately, with no further product formation detected by TLC and GC. Re-irradiation promptly restored the reaction, with a reaction rate comparable to that observed during the initial irradiation period. These observations demonstrate that continuous light irradiation is essential for efficient conversion and strongly support a photoinitiated radical pathway (Fig. 3). The immediate cessation of product formation upon removal of the light source also argues against a thermally initiated or self-sustaining radical chain process.^3,26^

**Scheme 2:**
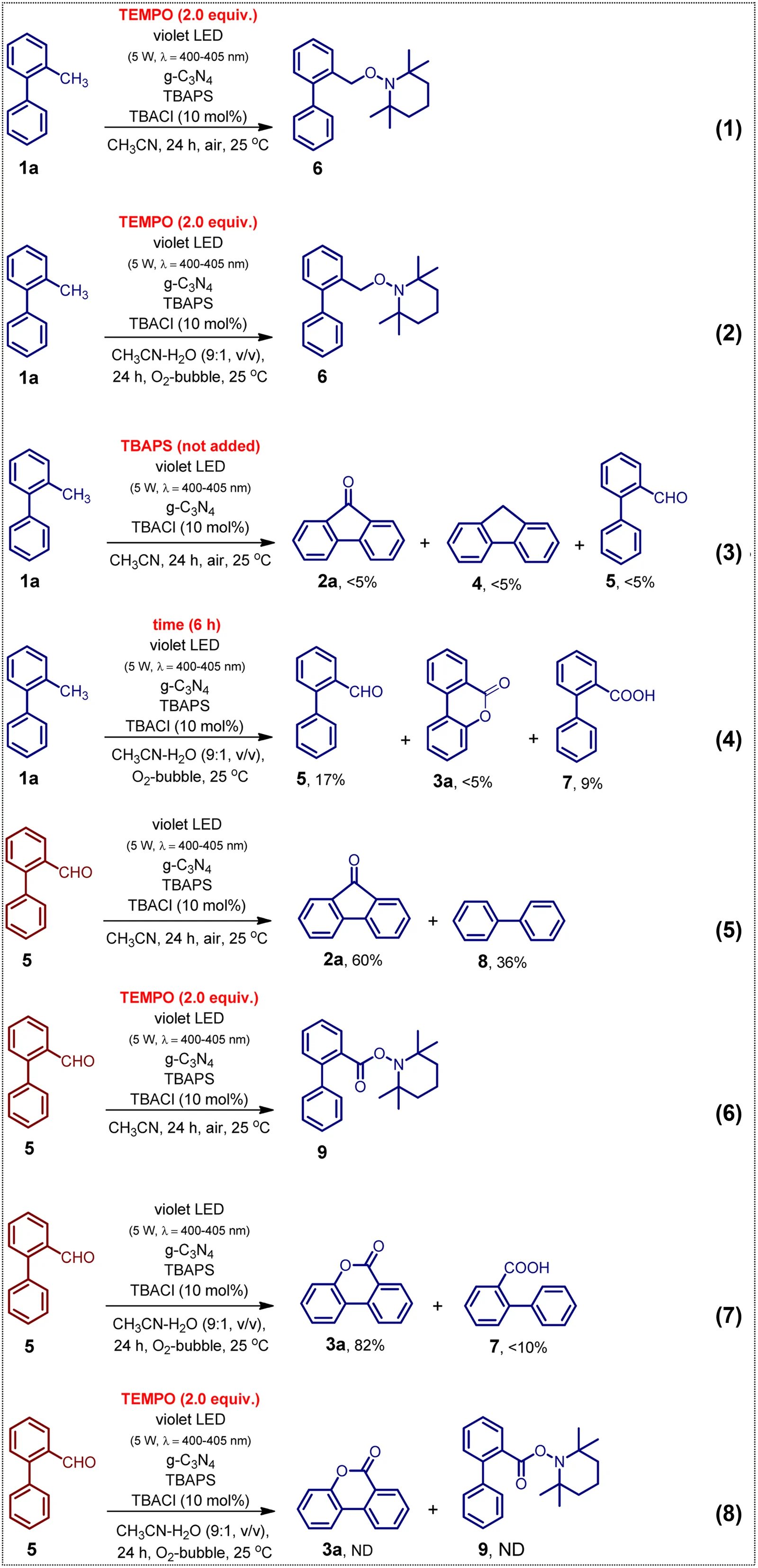
Control experiments.

**Scheme 3:**
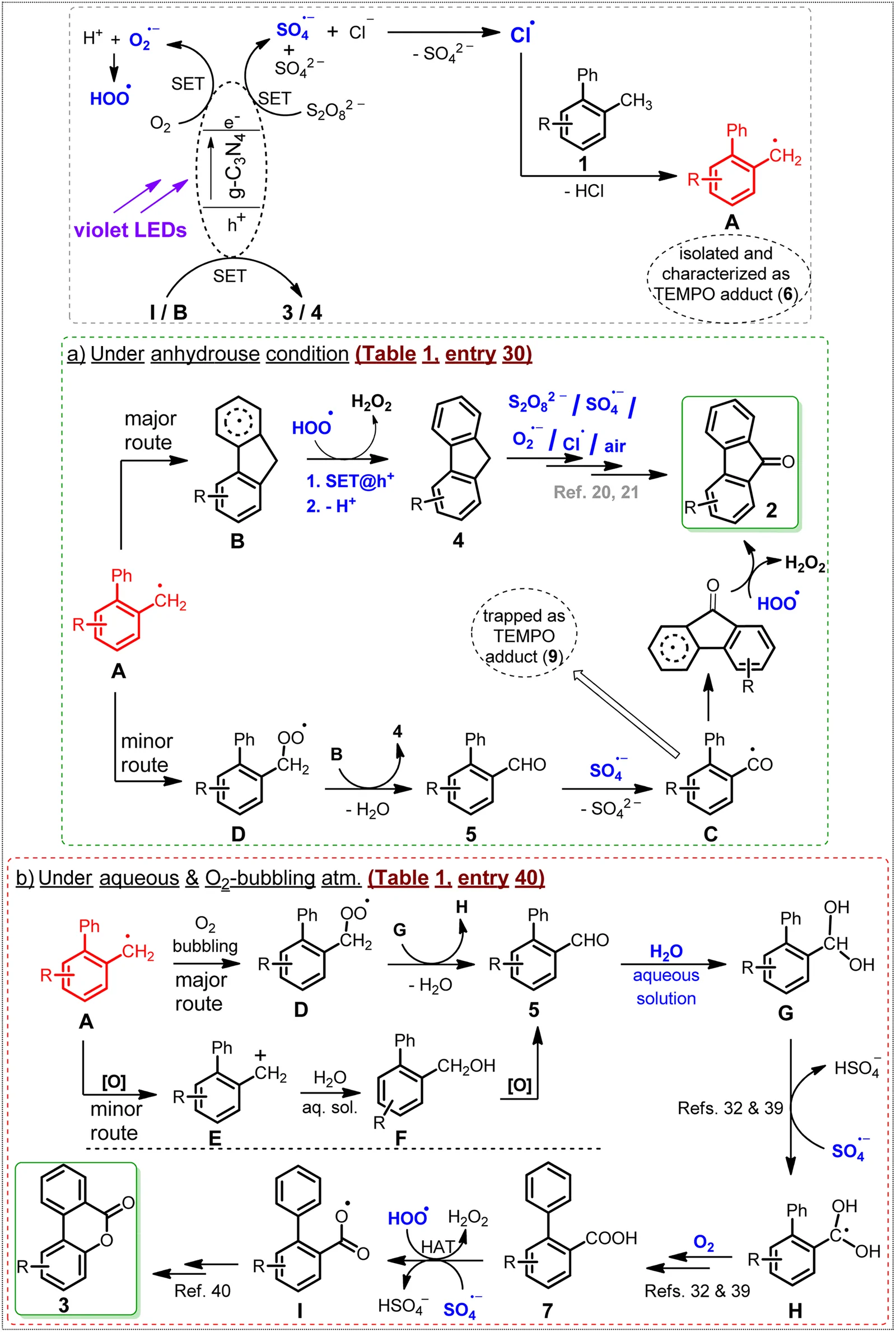
A plausible mechanism for the solvent-controlled divergent synthesis of fluorenones (2) and benzo[*c*]coumarins (3) from 2-methyl-1,1′-biaryls (1).

A number of control experiments were then carried out in order to gain insight into the reaction mechanism (Scheme 2, eqn (1)–(8)). In the absence of visible-light, no product was formed (Table 1, entry 7), confirming that irradiation is necessary. Similarly, the lack of g-C_3_N_4_ resulted 2a in trace yield, indicating its crucial role (Table 1, entry 32). When the reaction was conducted in a nitrogen atmosphere, 2a was not detected; instead, 9*H*-fluorene (4) was obtained in 90% yield (Table 1, entry 33), highlighting the need of oxygen in the reaction. In this section, for clarity, we repeat the key differences between the two optimized conditions (Table 1): entry 30 employs dry CH_3_CN, ambient air, and TBAPS (1.3 equiv.), whereas entry 40 uses a CH_3_CN–water (9 : 1, v/v) mixture, continuous O_2_-bubbling, and TBAPS (2.0 equiv.). Under both conditions (entries 30 and 40), addition of TEMPO (2.0 equiv.) completely suppressed product formation, and the TEMPO-trapped adduct 6 was obtained quantitatively (eqn (1) and (2), Scheme 2), confirming that the reaction is initiated by formation of 2-phenylbenzyl radical intermediates (A, Scheme 3). Omitting TBAPS under the conditions of entry 30 severely inhibited the reaction, giving only trace amounts (<5%) of 2a, 4, and 5, underscoring its essential role (eqn (3), Scheme 2). Shortening the reaction time to 6 h under the conditions of entry 40 afforded a mixture of aldehyde 5 (17%) and carboxylic acid 7 (9%), along with trace lactone 3a (<5%, eqn (4), Scheme 2). When aldehyde 5 was subjected to the conditions of entry 30, 2a (60%) and biphenyl (8, 36%) were formed (eqn (5), Scheme 2); however, addition of TEMPO suppressed these products and yielded the benzoyl–TEMPO adduct 9 (eqn (6), Scheme 2), supporting the formation of a free benzoyl radical *via* formyl C–H abstraction. Notably, under the optimized condition (Table 1, entry 30), precursor 1a afforded 2a in 92% yield and biphenyl 8 was not detected, indicating that aldehyde 5 is not a major intermediate in the synthesis of fluorenones (2) from 2-methyl-1,1′-biaryls (1). Instead, 2a is primarily formed through intramolecular cyclization of 2-phenylbenzyl radicals (A) into 9*H*-fluorene (4), followed by rapid oxidation to fluorenone (2a). When aldehyde 5 was treated under the CH_3_CN–water (9 : 1, v/v) medium with continuous O_2_-bubbling conditions (entry 40), lactone 3a was selectively obtained in 82% yield (eqn (7), Scheme 2). Interestingly, in these aqueous, oxygen-rich conditions, TEMPO totally inhibited the formation of lactone 3a, while the benzoyl-TEMPO adduct 9 was not observed (eqn (8), Scheme 2). These results indicate that both pathways—formation of fluorenones (2) and benzo[*c*]coumarins (3)—proceed through radical intermediates and share a common 2-phenylbenzyl radical (A), but the presence of water, higher TBAPS loading, and increased oxygen concentration significantly alter the reaction outcome. Under aqueous, oxygen-rich conditions, the 2-phenylbenzyl radical (A) is readily converted into aldehyde 5, which likely undergoes rapid hydration to form a gem-diol intermediate (G, Scheme 3). This intermediate is then oxidized to carboxylic acid 7, followed by intramolecular cyclization to afford benzo[*c*]coumarins (3). Of course, the observed selectivity switch cannot be attributed simply to solvent polarity; rather, it is likely governed by the combined effects of dissolved oxygen concentration, photocatalyst charge separation, interfacial water–g-C_3_N_4_ interactions, and the relative propensity of the initially generated reactive intermediates toward radical trapping *versus* intramolecular cyclization.

On the basis of control experiments (Scheme 2) and literature reports,^29–31,34^ a plausible mechanism for the solvent-controlled divergent synthesis of fluorenones (2) and benzo[*c*]coumarins (3) from 2-methyl-1,1′-biaryls (1) is proposed in Scheme 3. Upon visible-light irradiation, the g-C_3_N_4_ photocatalyst is excited to generate electron–hole pairs. Single-electron transfer (SET) between conduction-band photoelectrons and O_2_ or S_2_O_8_^2−^ produces the superoxide radical anion (O_2_˙^−^) and the sulfate radical anion (SO_4_˙^−^), respectively. The highly reactive chlorine radical (Cl˙) is subsequently formed *via* the reaction of chloride ions with SO_4_˙^−^.^35^ Hydrogen atom abstraction from the benzylic position of 2-methyl-1,1′-biaryls (1) by Cl˙ then generates intermediate A.^36^ It is worthy to mention that the direct oxidation of Cl^−^ by the photogenerated valence-band holes of g-C_3_N_4_ photocatalyst is thermodynamically unfavorable. The valence-band edge of g-C_3_N_4_ photocatalyst is approximately +1.4 to +1.6 V *vs.* NHE, whereas oxidation of Cl^−^ to a chlorine radical requires a substantially more positive potential (Cl˙/Cl^−^, *ca.* +2.4 V *vs.* NHE).^28–31^

Under anhydrous conditions and an ambient air atmosphere (Table 1, entry 30), intermediate A undergoes 5-*exo*-trig cyclization to afford intermediate B.^19–21^ Subsequent SET with the photogenerated hole (h^+^) and deprotonation of B produce fluorene derivatives 4, which is further oxidized by reactive oxygen, sulfate and/or chlorine species to furnish the desired fluorenones (2).^20,21^ Of course, even under limited oxygen supply, product 2 may still be formed in some quantity from aldehyde 5*via* SO_4_˙^−^-assisted formation of benzoyl radical species C, followed by intramolecular cyclization and oxidative rearomatization (Scheme 3).^29–31,36^

In contrast, under aqueous conditions (CH_3_CN–H_2_O, 9 : 1, v/v, solvent system) with an oxygen-bubbling atmosphere (Table 1, entry 40), intermediate A preferentially reacts with oxygen to generate peroxy radical D, which subsequently collapses to give [1,1′-biaryl]-2-carbaldehydes 5.^37^ Alternatively, aldehyde 5 may also form *via* direct oxidation of A to cationic intermediate E, followed by nucleophilic water addition and subsequent classical oxidation steps (Scheme 3).^38^ In the presence of water, aldehyde 5 undergoes hydration to generate gem-diol G, which is further oxidized by SO_4_˙^−^ to intermediate H and then to [1,1′-biaryl]-2-carboxylic acid 7.^32,39^ The *in situ* generated 7 subsequently reacts with HOO˙ or SO_4_˙^−^ to form benzoyloxy radical I.^14,15^ Intramolecular radical addition, followed by hydrogen atom transfer with HOO˙/SO_4_˙^−^ or SET with the photogenerated hole (h^+^) and deprotonation, ultimately affords product 3 (Scheme 3).^40^

## 3 Conclusion

We have developed a solvent-regulated, metal-free g-C_3_N_4_ visible-light photoredox methodology for the selective synthesis of two distinct heterocyclic frameworks: fluorenones and benzo[*c*]coumarins, using a common 2-methyl-1,1′-biaryl precursor. Though both products originate from a shared 2-phenylbenzyl radical intermediate, small variations in the reaction conditions have a substantial effect on the reaction pathway and product distribution. In an oxygen-rich aqueous solution, the 2-phenylbenzyl radical is efficiently oxidized to the corresponding carboxylic acid intermediates, which subsequently undergo cyclization to furnish benzo[*c*]coumarins. In contrast, under anhydrous conditions, the same radical intermediate preferentially cyclizes to form fluorene derivatives, which are further oxidized to produce fluorenones. This study unequivocally shows that product selectivity from a common intermediate can be efficiently controlled by precise modification of reaction parameters. We are currently expanding this methodology to other benzylic systems and exploring new solvent-triggered reaction pathways. We believe that this approach will be widely used in synthetic chemistry, green chemistry, pharmaceutical chemistry, photocatalysis, and other fields.

## Author contributions

All of the authors contributed to the writing of the manuscript. The final version of the work has received the approval from all authors. Pooja's responsibilities include experiment design, execution, methodology, data analysis, interpretation, writing – original draft and review. On the other hand, conceptualization, validation, and formal analysis are among Meena's tasks. Palani Natarajan is responsible for financial support, writing and supervision.

## Conflicts of interest

The authors declare that they have no conflicts of interest.

## Supplementary Material

RA-016-D6RA06292J-s001

## Data Availability

The data supporting this article have been included as part of the supplementary information (SI). Supplementary information: general aspects, procedure, experimental characterization data for products, images of irradiation source and copy of NMR spectra. See DOI: https://doi.org/10.1039/d6ra06292j.
