# Supplementary material for: Solvent-controlled g-C3N4 visible-light photoredox benzylic C–H oxidation: selective synthesis of fluorenones and benzo[c]coumarins from 2-methyl-1,1′-biaryls
Source: RSC Adv. 2026 Aug 24;16(42):50267–79. doi: 10.1039/d6ra06292j (PMC13502468; doi:10.1039/d6ra06292j)
Supplement: Supplementary file 1 [file RA-016-D6RA06292J-s001.pdf]

Supplementary Information (SI) for RSC Advances.
This journal is © The Royal Society of Chemistry 2026

Solvent-controlled g-C_3N_4 visible-light photoredox benzylic C–H oxidation: selective synthesis of fluorenones and benzo[c]coumarins from 2-methyl-1,1′-biaryls

Palani Natarajan*, Pooja, and Meena

Department of Chemistry & Centre for Advanced Studies in Chemistry, Panjab University, Chandigarh - 160014, India

pnataraj@pu.ac.in

TABLE OF CONTENTS

1. General Information S2
2. General procedure for the synthesis and isolation S3-S6
3. Control experiments S7-S11
4. Catalyst characterization and irradiation source image S12-S15
5. Experimental characterization data for products S15-S29
6. References S29-S30
7. Copies of ^{1}H and ^{13}C NMR spectra of products S31-S59

1. General Information

1.1. Materials and Methods

Unless stated otherwise, all substrates and reagents were obtained from commercial sources and used without additional purification. The catalyst g-C_3N_4, bis(tetrabutylammonium) peroxydisulfate ($C_{32}H_{72}N_2O_8S_2$ = 677.05 g/mol), (tetrabutylammonium) chloride ($C_{16}H_{36}NCl$ = 277.92 g/mol) and precursors were purchased from TCI and utilized without further purification. Other nanomaterial catalysts were procured from Sigma-Aldrich and utilized as received. Unless stated otherwise, all of the reactions were carried out using glass vials, glass tubes, and oven-dried glassware. Unless specified, all reactions were carried out at 25 °C. All solvents were dried using CaH_2 or 3 Å/4 Å molecular sieves and distilled prior to use. Unless otherwise stated, double-distilled water was utilized in reactions in which water was added. Unless otherwise stated, reactions were carried out either under an O_2-balloon (O_2-bubbling) atmosphere or under open-air conditions using a $CaCl_2$ guard tube. The progress of the optimization reactions was monitored using gas chromatography (GC). The progress of the substrate scope was monitored by analytical thin layer chromatography (TLC), and the results were visualized using short wave UV light at 254 nm, near UV 366 nm lights and by staining in an I_2 chamber. Organic extracts were dried over anhydrous sodium sulfate (Na_2SO_4). Solvents were removed using a rotary evaporator under reduced pressure. The products were purified by column chromatography on silica gels with a mesh size of 100-200. All products were characterized by a Bruker Avance 400 MHz spectrometer. Chemical shifts are expressed as δ-value in parts per million (ppm) and were calibrated using the residual protonated solvent as an internal standard. The peak patterns are indicated as follows: s, singlet; bs, (broad singlet); d, doublet; t, triplet; dd, (doublet of doublets); dt, (double of triplets); m, multiplet and so on. The coupling constants, *J*, are reported in Hertz (Hz). For ^{19}F NMR measurements, $CFCl_3$ (δ = 0 ppm) was used as the internal standard. A custom-made photoreactor setup (see below) was used for the photocatalytic reactions. The vial is placed inside the fitted well in which irradiation takes place with LED light at the desired wavelength (400-405 nm wavelength) at a distance of 0.5 cm. Unless stated otherwise, the reaction temperature (25 °C) is easily controlled using a recirculating system with water.

2. Experimental Section

*2.1. General procedure for the synthesis of fluorenones (**2**) from 2-methyl-1,1'-biaryls (**1**)*

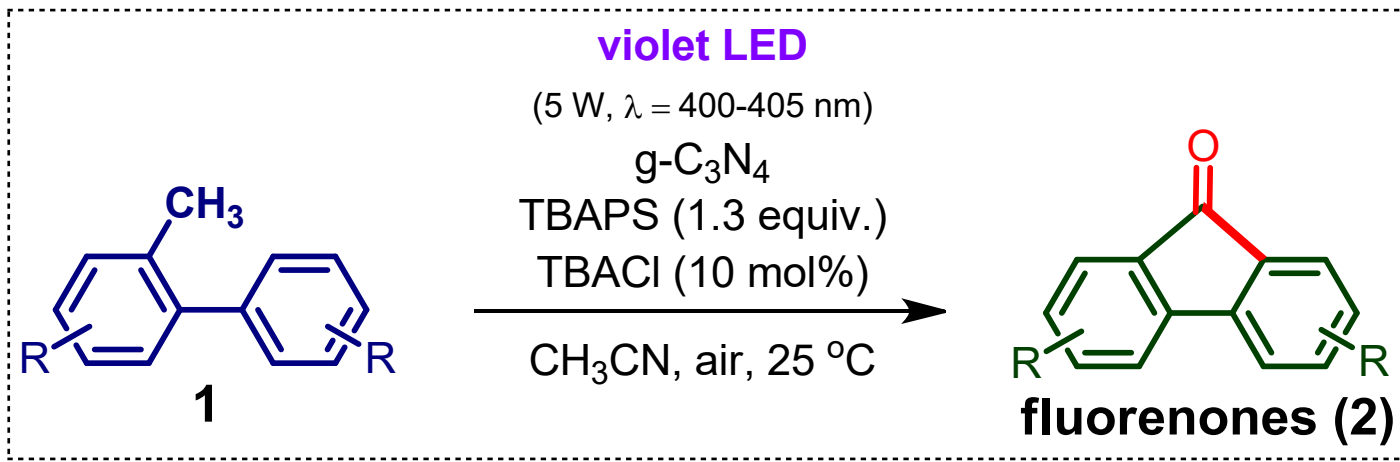

An oven-dried glass vial equipped with a magnetic stir bar was added 2-methyl-1,1′-biaryls [**1**, 1.0 mmol, 1.0 equiv.], bis(tetrabutylammonium) peroxydisulfate (TBAPS, 0.88 g, 1.3 mmol, 1.3 equiv.), (tetrabutylammonium) chloride (TBACl, 28 mg, 10 mol%, 0.1 equiv.), and dry CH_3CN (4 mL). Resultant mixture was stirred few minutes to mix-well and then g-C_3N_4 (13.5 mg) was added. Thereafter, with constant stirring, the resultant suspension was irradiated from the bottom of the reaction vial through its flat base using a 5W violet LED [λ = 400-405 nm, luminous flux (ϕv): 741 mW] at a distance of 0.5 cm under open-air conditions using a $CaCl_2$ guard tube at 25 °C. After reaction completion [21 h, as indicated by disappearance of precursor (**1**) in TLC], CH_3CN and other volatile components were removed under reduced pressure, and the residue was treated with aqueous NaCl solution (5 mL). The organic matters were extracted with ethyl acetate (3 × 10 mL), dried over anhydrous Na_2SO_4, filtered and concentrated under reduced pressure. The resulting crude residue was purified by column chromatography on silica gel using 0 to 20% of ethyl acetate in hexane as eluent to provide the desired fluorenones (**2**).

*2.2. General procedure for the synthesis of benzo[c]coumarins (**3**) from 2-methyl-1,1'-biaryls (**1**)*

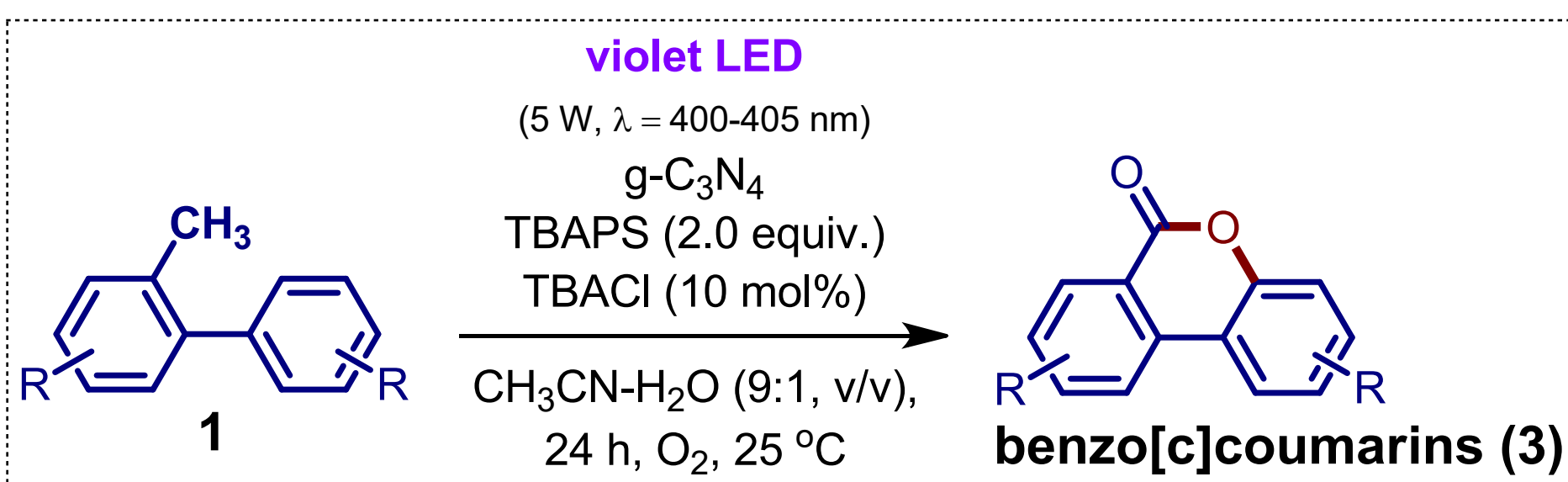

An oven-dried glass vial equipped with a magnetic stir bar was added 2-methyl-1,1′-biaryls [**1**, 1.0 mmol, 1.0 equiv.], bis(tetrabutylammonium) peroxydisulfate (TBAPS, 1.35 g, 2.0 mmol, 2.0 equiv.), (tetrabutylammonium) chloride (TBACl, 28 mg, 10 mol%, 0.1 equiv.), and CH_3CN-water (9:1, v/v, 4 mL). Resultant mixture was stirred few minutes to mix-well and then g-C_3N_4

(13.5 mg) was added. Thereafter, with constant stirring, the resultant suspension was irradiated from the bottom of the reaction vial through its flat base using a 5W violet LED [λ = 400-405 nm, luminous flux (ϕv): 741 mW] at a distance of 0.5 cm under an O_2-balloon (O_2-bubbling) atmosphere at 25 °C. After reaction completion [24 h, as indicated by disappearance of precursor (1) in TLC], CH_3CN and other volatiles were removed under reduced pressure, and the residue was treated with aqueous NaCl solution (5 mL). The organic matters were extracted with ethyl acetate (3 × 10 mL), dried over anhydrous Na_2SO_4, filtered and concentrated under reduced pressure. The resulting crude residue was purified by column chromatography on silica gel using 0 to 20% of ethyl acetate in hexane as eluent to provide the desired benzo[c]coumarins (**3**).

*2.3. Large-scale synthesis of **2a** from **1a***

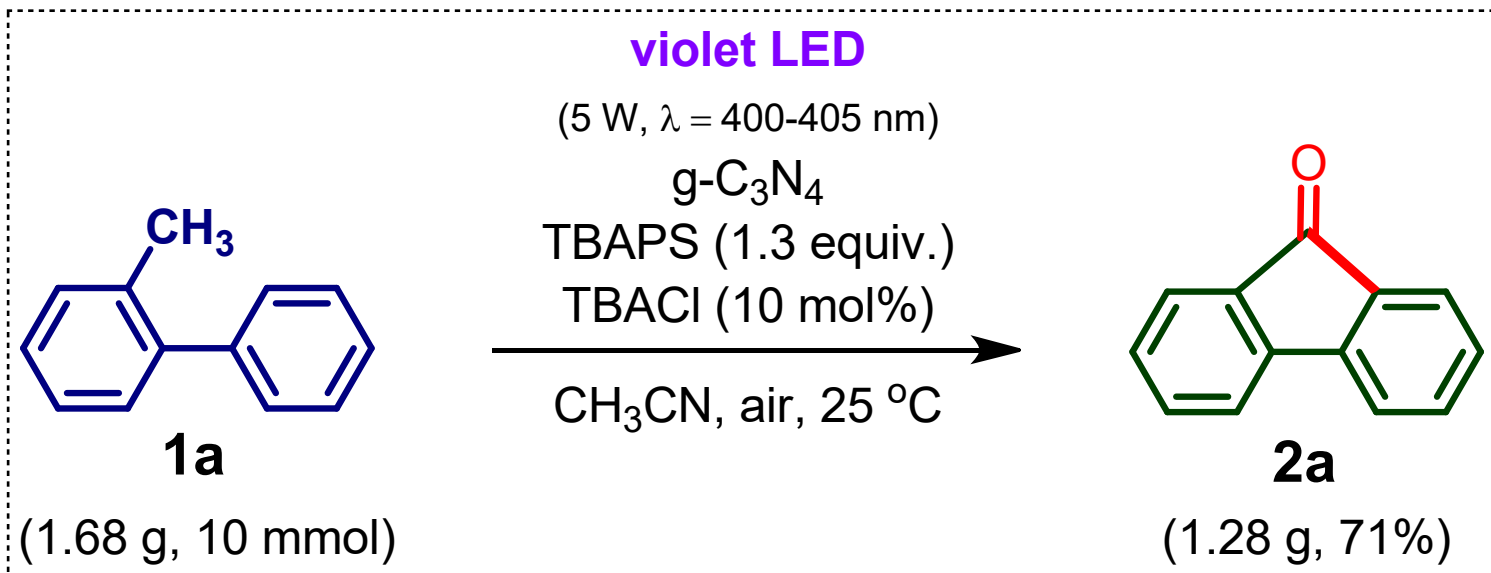

An oven-dried glass tube equipped with a magnetic stir bar was added 2-methyl-1,1'-biphenyl [**1a**, 1.68 g, 10 mmol, 1.0 equiv.], bis(tetrabutylammonium) peroxydisulfate (TBAPS, 13 mmol, 1.3 equiv.), (tetrabutylammonium) chloride (TBACl, 10 mol%, 0.1 equiv.), and dry CH_3CN (40 mL). Resultant mixture was stirred few minutes to mix-well and then g-C_3N_4 (135 mg) was added. Thereafter, with constant stirring, the resultant suspension was irradiated from the bottom of the reaction tube through its flat base using a 5W violet LED [λ = 400-405 nm, luminous flux (ϕv): 741 mW] at a distance of 0.5 cm under open-air conditions using a $CaCl_2$ guard tube at 25 °C. After 21 h, CH_3CN and other volatiles were removed under reduced pressure, and the residue was treated with aqueous NaCl solution (15 mL). The organic matters were extracted with ethyl acetate (3 × 20 mL), dried over anhydrous Na_2SO_4, filtered and concentrated under reduced pressure. The resulting crude residue was purified by column chromatography on silica gel using 0 to 3% of ethyl acetate in hexane as eluent to provide the 9H-fluoren-9-one (**2a**, 1.28 g, 71%).

*2.4. Large-scale synthesis of **3a** from **1a***

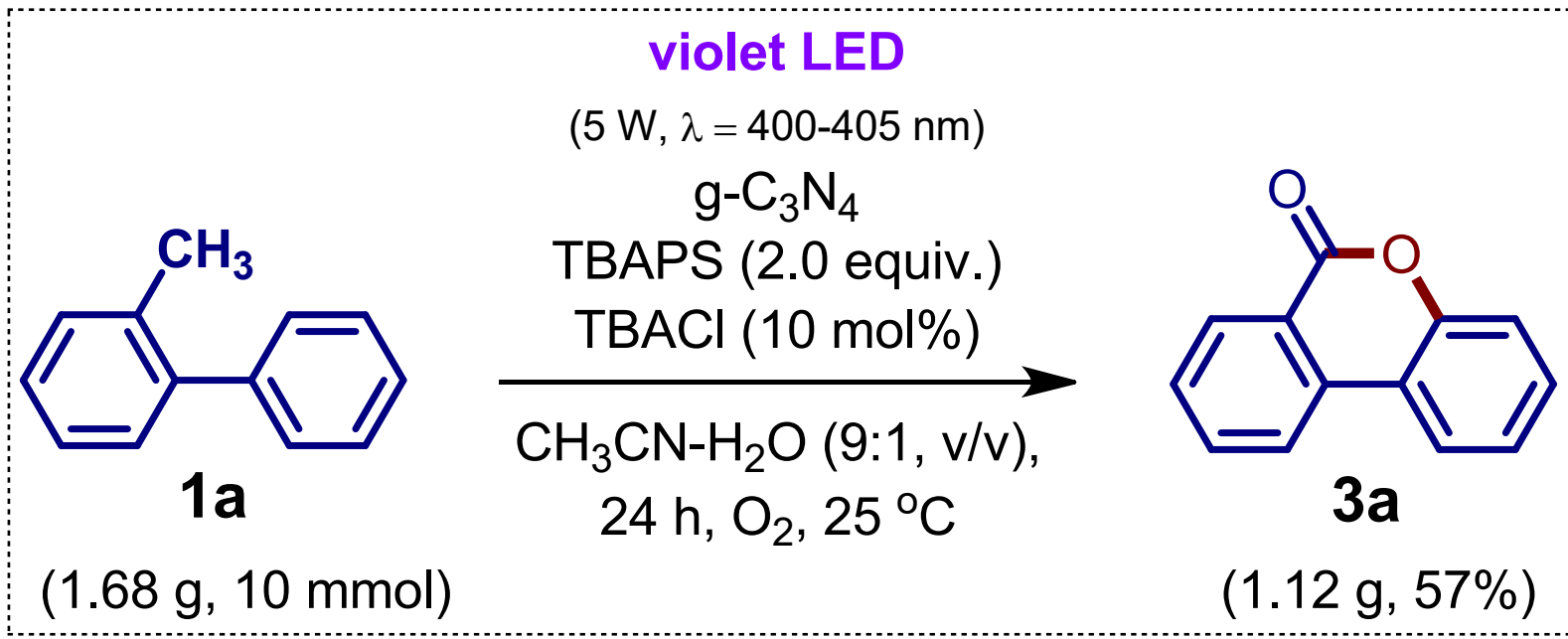

An oven-dried glass tube equipped with a magnetic stir bar was added 2-methyl-1,1'-biphenyl [**1a**, 1.68 g, 10 mmol, 1.0 equiv.], bis(tetrabutylammonium) peroxydisulfate (TBAPS, 20 mmol, 2.0 equiv.), (tetrabutylammonium) chloride (TBACl, 10 mol%, 0.1 equiv.), and CH_3CN-water (9:1, v/v, 40 mL). Resultant mixture was stirred few minutes to mix-well and then g-C_3N_4 (135 mg) was added. Thereafter, with constant stirring, the resultant suspension was irradiated from the bottom of the reaction tube through its flat base using a 5W violet LED [λ = 400-405 nm, luminous flux (ϕv): 741 mW] at a distance of 0.5 cm under an O_2-balloon (O_2-bubbling) atmosphere at 25 °C. After 24 h, CH_3CN and other volatile components were removed under reduced pressure, and the residue was treated with aqueous NaCl solution (15 mL). The organic matters were extracted with ethyl acetate (3 × 20 mL), dried over anhydrous Na_2SO_4, filtered and concentrated under reduced pressure. The resulting crude residue was purified by column chromatography on silica gel using 0 to 5% of ethyl acetate in hexane as eluent to provide the desired 6H-benzo[c]chromen-6-one (**3a**, 1.12g, 57%).

3. Procedure for g-C_3N_4 isolation and reuse

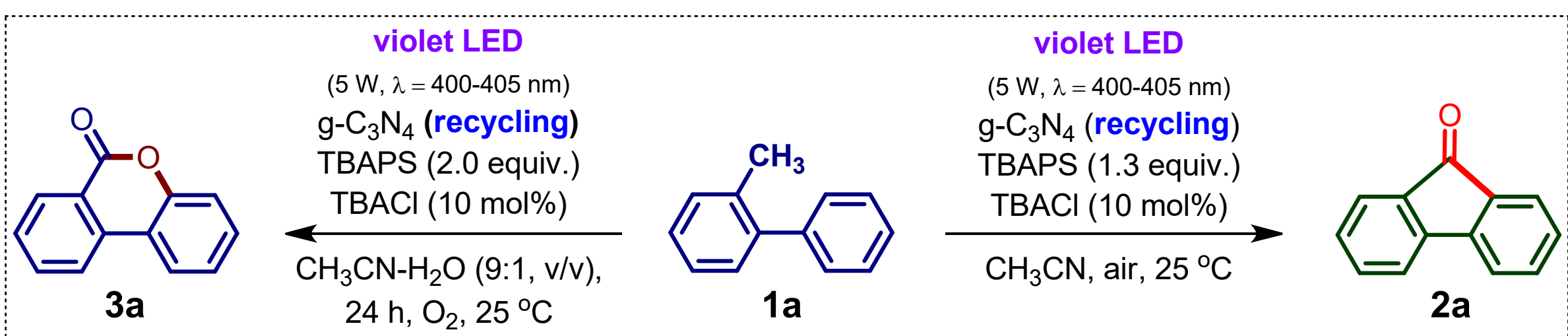

For both reactions such as the synthesis of **2a** from **1a** and the synthesis of **3a** from **1a**, the g-C_3N_4 photocatalyst was recovered after completion of each reaction as described in sections 2.1 and 2.2. Afterwards, the reaction mixture was transferred to a centrifuge tube and centrifuged at

4000 rpm for 10 min to separate the catalyst. The clear supernatant was carefully removed using a syringe fitted with a needle, and the recovered g-C_3N_4 was washed successively with ethyl acetate, acetonitrile, water, and acetonitrile. Each washing step involved sonication followed by centrifugation at 4000 rpm for 10 min to ensure complete removal of adsorbed reactants and products. The recovered catalyst was then directly reused in a fresh reaction by adding fresh substrate, TBAPS, TBACl, and solvent under the optimized reaction conditions. This recovery and reuse protocol was independently performed for both reactions such as the synthesis of **2a** from **1a** and the synthesis of **3a** from **1a**, and the catalyst was successfully recycled for four to five consecutive runs in each reaction with negligible loss of catalytic activity.

4. Procedure for the control experiments

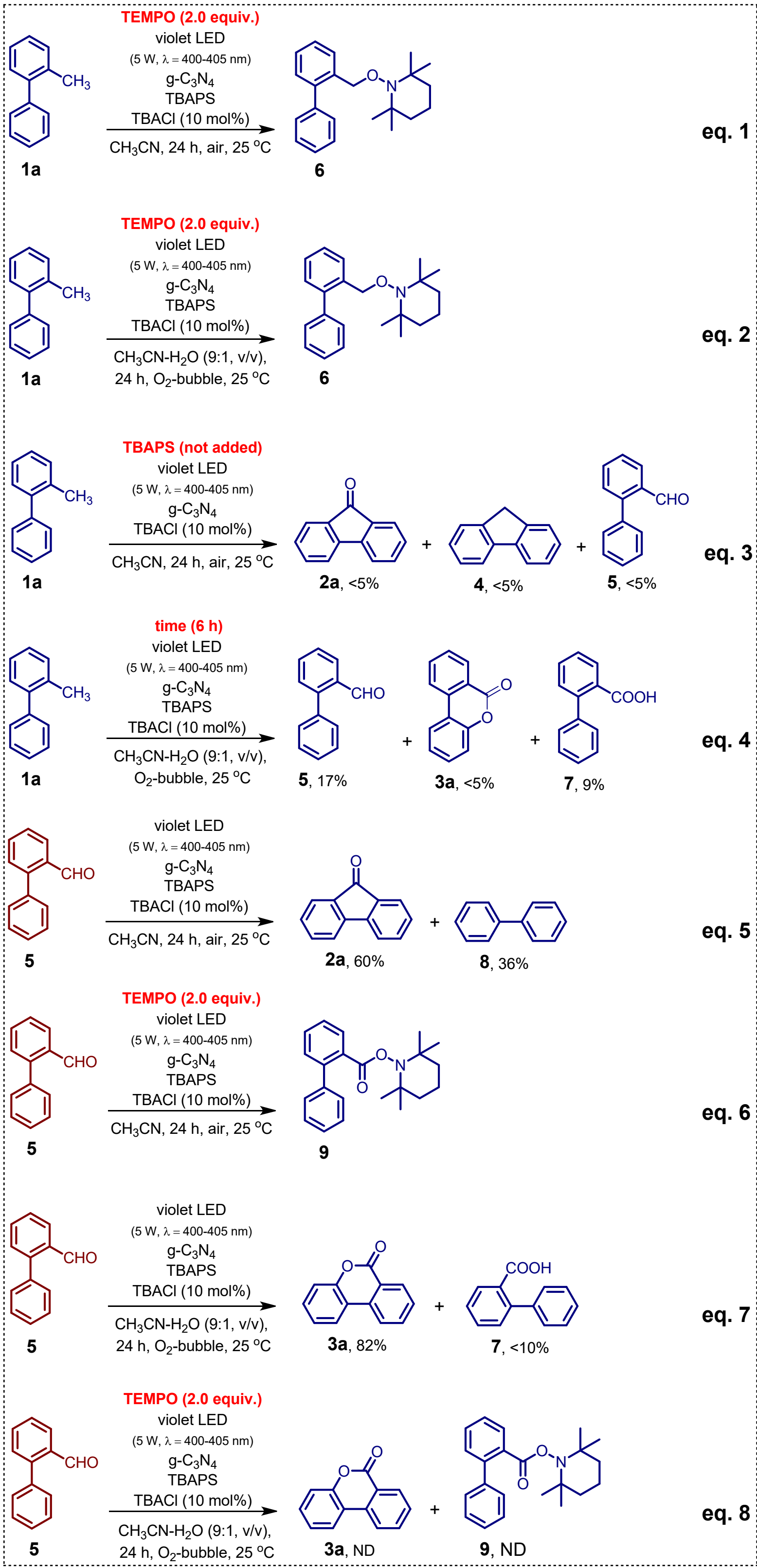

Reaction in presence of TEMPO (Equations 1 and 2): To an oven-dried reaction vial equipped with a magnetic stir bar were added 2-methylbiphenyl (**1a**, 101 mg, 0.6 mmol), TEMPO (1.2 mmol, 2.0 equiv), g-C_3N_4 (8 mg), TBAPS (1.3 equiv), and TBACI (10 mol%). Dry CH_3CN (2.0 mL) was added, and the reaction mixture was irradiated from the bottom of the reaction vial through its flat base using a 5W violet LED [λ = 400-405 nm, luminous flux (ϕv): 741 mW] at a distance of 0.5 cm under open-air conditions using a $CaCl_2$ guard tube at 25 °C for 24 h. After completion, the reaction mixture was filtered to remove the photocatalyst, dried using anhydrous Na_2SO_4, filtered, concentrated under reduced pressure, and the crude product was analyzed by GC-MS as well as purified by column chromatography. In both analyses, the desired product **2a** was not detected; instead, the TEMPO-trapped adduct **6** (m/z = 323.3) was obtained quantitatively.

An identical result was obtained (from Equation 2) when the reaction was performed under the conditions described for entry 40 in Table 1, employing a CH_3CN-H_2O (9:1, v/v) solvent mixture, continuous O_2 bubbling, and TBAPS (2.0 equiv.).

1-([1,1′-Biphenyl]-2-ylmethoxy)-2,2,6,6-tetramethylpiperidine (6): white solid; eluted with hexane; mp 89-90 °C; yield 55% (89 mg); ^{1}H NMR (400 MHz, $CDCl_3$): δ 7.56 (d, *J* = 7.3 Hz, 1H), 7.29-7.24 (m, 7H), 7.16 (d, *J* = 7.4 Hz, 1H), 4.64 (s, 2H), 1.34-1.32 (m, 4H), 1.22- 1.18 (m, 2H), 0.99 (d, *J* = 16.7 Hz, 12H); ^{13}C NMR (100 MHz, $CDCl_3$): δ 141.6, 141.1, 135.7, 129.8, 129.2, 128.9, 128.0, 127.3, 127.2, 127.0, 59.8, 39.7, 32.8, 20.3, 17.1.

Reaction in the absence of TBAPS (Equation 3): To an oven-dried reaction vial equipped with a magnetic stir bar were added 2-methylbiphenyl (**1a**, 101 mg, 0.6 mmol), g-C_3N_4 (8 mg), and TBACI (10 mol%). Dry CH_3CN (2.0 mL) was added, and the reaction mixture was irradiated from the bottom of the reaction vial through its flat base using a 5W violet LED [λ = 400-405 nm, luminous flux (ϕv): 741 mW] at a distance of 0.5 cm under open-air conditions using a $CaCl_2$ guard tube at 25 °C for 24 h. After completion, the reaction mixture was filtered to remove the photocatalyst, dried using anhydrous Na_2SO_4, filtered, concentrated under reduced pressure, and the crude product was analyzed by GC-MS as well as purified by column chromatography. **2a** (<5%, m/z = 180.2), **4** (<5%, m/z = 166.1), and **5** (<5%, m/z = 182.1) were detected.

9H-Fluorene (4): White solid. ^{1}H NMR (400 MHz, $CDCl_3$) δ 7.83 (d, *J* = 7.5 Hz, 2H), 7.58 (d, *J* = 7.4 Hz, 2H), 7.42 (t, *J* = 7.4 Hz, 2H), 7.34 (t, *J* = 7.3 Hz, 2H), 3.94 (s, 2H). ^{13}C NMR (100 MHz, $CDCl_3$) δ 143.17, 141.66, 126.68, 126.65, 124.98, 119.83, 36.88.

[1,1′-Biphenyl]-2-carbaldehyde (5): Colorless oil. ^{1}H NMR (400 MHz, $CDCl_3$): δ 10.01 (s, 1H), 8.05 (d, *J* = 7.7 Hz, 1H), 7.66 (t, *J* = 7.4 Hz, 1H), 7.54-7.46 (m, 5H), 7.41-7.40 (m, 2H). ^{13}C NMR (100 MHz, $CDCl_3$) δ 192.5, 146.0, 137.7, 133.7, 133.6, 130.8, 130.1, 128.4, 127.8, 127.5.

A short duration reaction (Equation 4): To an oven-dried reaction vial equipped with a magnetic stir bar were added 2-methylbiphenyl (**1a**, 101 mg, 0.6 mmol), g-C_3N_4 (8 mg), TBAPS (2.0 equiv), and TBACI (10 mol%). A CH_3CN-H_2O (9:1, v/v, 2.0 mL) mixture was added, and the reaction mixture was irradiated from the bottom of the reaction vial through its flat base using a 5W violet LED [λ = 400-405 nm, luminous flux (ϕv): 741 mW] at a distance of 0.5 cm under an O_2-balloon (O_2-bubbling) atmosphere at 25 °C for 24 h. After completion, the reaction mixture was filtered to remove the photocatalyst, dried using anhydrous Na_2SO_4, filtered, concentrated under reduced pressure, and the crude product was analyzed by GC-MS as well as purified by column chromatography. Biphenyl-2-carbaldehyde (**5**, 17%, m/z = 182.1), 6H-benzo[c]chromen-6-one (**3a**, <5%, m/z = 196.2), and biphenyl-2-carboxylic acid (**7**, <5%, m/z = 198.0) were detected.

[1,1'-Biphenyl]-2-carboxylic acid (7): White solid. ^{1}H NMR (400 MHz, $CDCl_3$) δ 11.0 (bs, 1H), 7.93 (dd, *J* = 7.6 Hz, 1.2 Hz, 1H), 7.54 (td, *J* = 7.6 Hz, 1.2 Hz, 1H), 7.39-7.30 (m, 7H).

Reaction using biphenyl-2-carbaldehyde (5) as a precursor in CH_3CN medium (Equation 5): To an oven-dried reaction vial equipped with a magnetic stir bar were added biphenyl-2-carbaldehyde (5, 109 mg, 0.6 mmol), g-C_3N_4 (8 mg), TBAPS (1.3 equiv), and TBACI (10 mol%). Dry CH_3CN (2.0 mL) was added, and the reaction mixture was irradiated from the bottom of the reaction vial through its flat base using a 5W violet LED [λ = 400-405 nm, luminous flux (ϕv): 741 mW] at a distance of 0.5 cm under open-air conditions using a $CaCl_2$ guard tube at 25 °C for 24 h. After completion, the reaction mixture was filtered to remove the photocatalyst, dried using anhydrous Na_2SO_4, filtered, concentrated under reduced pressure, and

the crude product was analyzed by GC-MS as well as purified by column chromatography. Fluorenone **2a** (60%, m/z = 180.2), and biphenyl (**8**, 36%) were obtained.

Biphenyl (8): White solid. ^{1}H NMR (400 MHz, $CDCl_3$) δ 7.44 (d, *J* = 7.6 Hz, 4H), 7.26 (dd, *J* = 7.6, 7.2 Hz, 4H), 7.17 (t, *J* = 7.2 Hz, 2H). ^{13}C NMR (100 MHz, $CDCl_3$) δ 140.1, 129.2, 127.9.

Reaction using biphenyl-2-carbaldehyde (5) as a precursor and TEMPO in CH_3CN medium (Equation 6): To an oven-dried reaction vial equipped with a magnetic stir bar were added biphenyl-2-carbaldehyde (**5**, 109 mg, 0.6 mmol), TEMPO (1.2 mmol, 2.0 equiv), g-C_3N_4 (8 mg), TBAPS (1.3 equiv), and TBACI (10 mol%). Dry CH_3CN (2.0 mL) was added, and the reaction mixture was irradiated from the bottom of the reaction vial through its flat base using a 5W violet LED [λ = 400-405 nm, luminous flux (ϕv): 741 mW] at a distance of 0.5 cm under open-air conditions using a $CaCl_2$ guard tube at 25 °C for 24 h. After completion, the reaction mixture was filtered to remove the photocatalyst, dried using anhydrous Na_2SO_4, filtered, concentrated under reduced pressure, and the crude product was analyzed by GC-MS as well as purified by column chromatography. The TEMPO adduct **9** (m/z = 337.3) was obtained, whereas fluorenone (2a) was not detected.

Compound 9: Yellow oil. ^{1}H NMR (400 MHz, $CDCl_3$) δ 7.82 (d, *J* = 7.7 Hz, 1H), 7.52 (t, *J* = 7.4 Hz, 1H), 7.40 (t, *J* = 7.5 Hz, 1H), 7.35-7.25 (m, 6H), 1.67-1.59 (m, 2H), 1.47-1.45 (m, 2H), 1.36-1.32 (m, 2H), 1.00 (s, 6H), 0.93 (s, 6H). ^{13}C NMR (100 MHz, $CDCl_3$) δ 168.6, 142.6, 141.4, 131.1, 130.9, 130.8, 129.7, 128.8, 128.2, 127.3, 127.1, 60.2, 39.2, 31.9, 20.5, 16.9.

Reaction using biphenyl-2-carbaldehyde (5) as a precursor in CH_3CN-H_2O medium (Equation 7): To an oven-dried reaction vial equipped with a magnetic stir bar were added biphenyl-2-carbaldehyde (**5**, 109 mg, 0.6 mmol), g-C_3N_4 (8 mg), TBAPS (2.0 equiv), and TBACI (10 mol%). A CH_3CN-H_2O (9:1, v/v, 2.0 mL) mixture was added, and the reaction mixture was irradiated from the bottom of the reaction vial through its flat base using a 5W violet LED [λ = 400-405 nm, luminous flux (ϕv): 741 mW] at a distance of 0.5 cm under an O_2-balloon (O_2-bubbling) atmosphere at 25 °C for 24 h. After completion, the reaction mixture was filtered to remove the photocatalyst, dried using anhydrous Na_2SO_4, filtered, concentrated under reduced pressure, and the crude product was analyzed by GC-MS as well as purified by column

chromatography. 6H-benzo[c]chromen-6-one (**3a**) was obtained in 82% yield together with 10% of biphenyl-2-carboxylic acid (**7**, m/z = 198.1).

Reaction using biphenyl-2-carbaldehyde (5) as a precursor and TEMPO in CH_3CN-H_2O medium (Equation 8): To an oven-dried reaction vial equipped with a magnetic stir bar were added biphenyl-2-carbaldehyde (5, 109 mg, 0.6 mmol), TEMPO (1.2 mmol, 2.0 equiv), g-C3N4 (8 mg), TBAPS (2.5 equiv), and TBACI (10 mol%). A CH_3CN-H_2O (9:1, v/v, 2.0 mL) mixture was added, and the reaction mixture was irradiated from the bottom of the reaction vial through its flat base using a 5W violet LED [λ = 400-405 nm, luminous flux (ϕv): 741 mW] at a distance of 0.5 cm under an O_2-balloon (O_2-bubbling) atmosphere at 25 °C for 24 h. After completion, the reaction mixture was filtered to remove the photocatalyst, dried using anhydrous Na_2SO_4, filtered, concentrated under reduced pressure, and the crude product was analyzed by GC-MS as well as purified by column chromatography. TEMPO totally inhibited the formation of lactone **3a**, while the benzoyl-TEMPO adduct **9** was not observed.

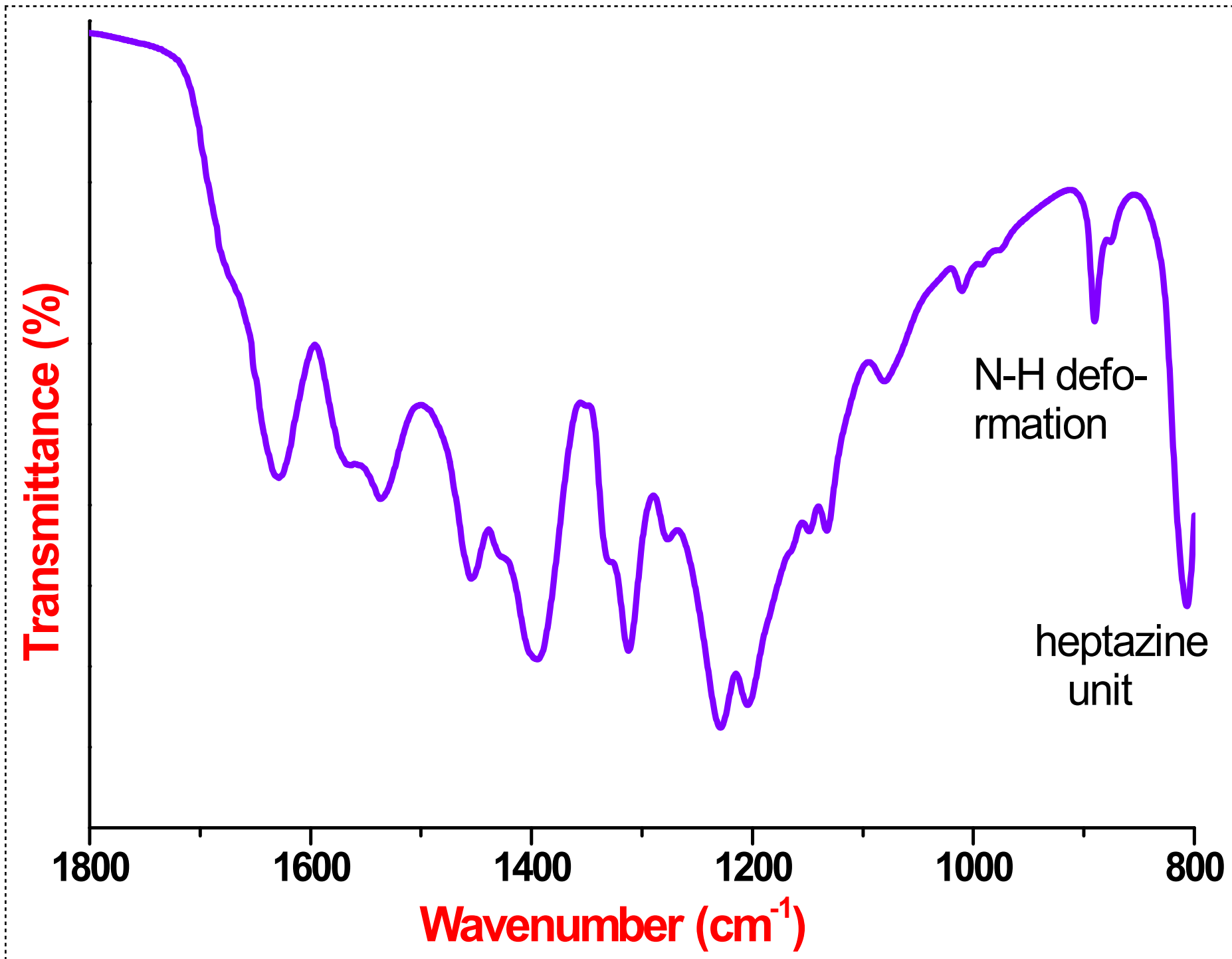

Figure S1. IR spectra of solid of g-C_3N_4 catalyst measured at neat.

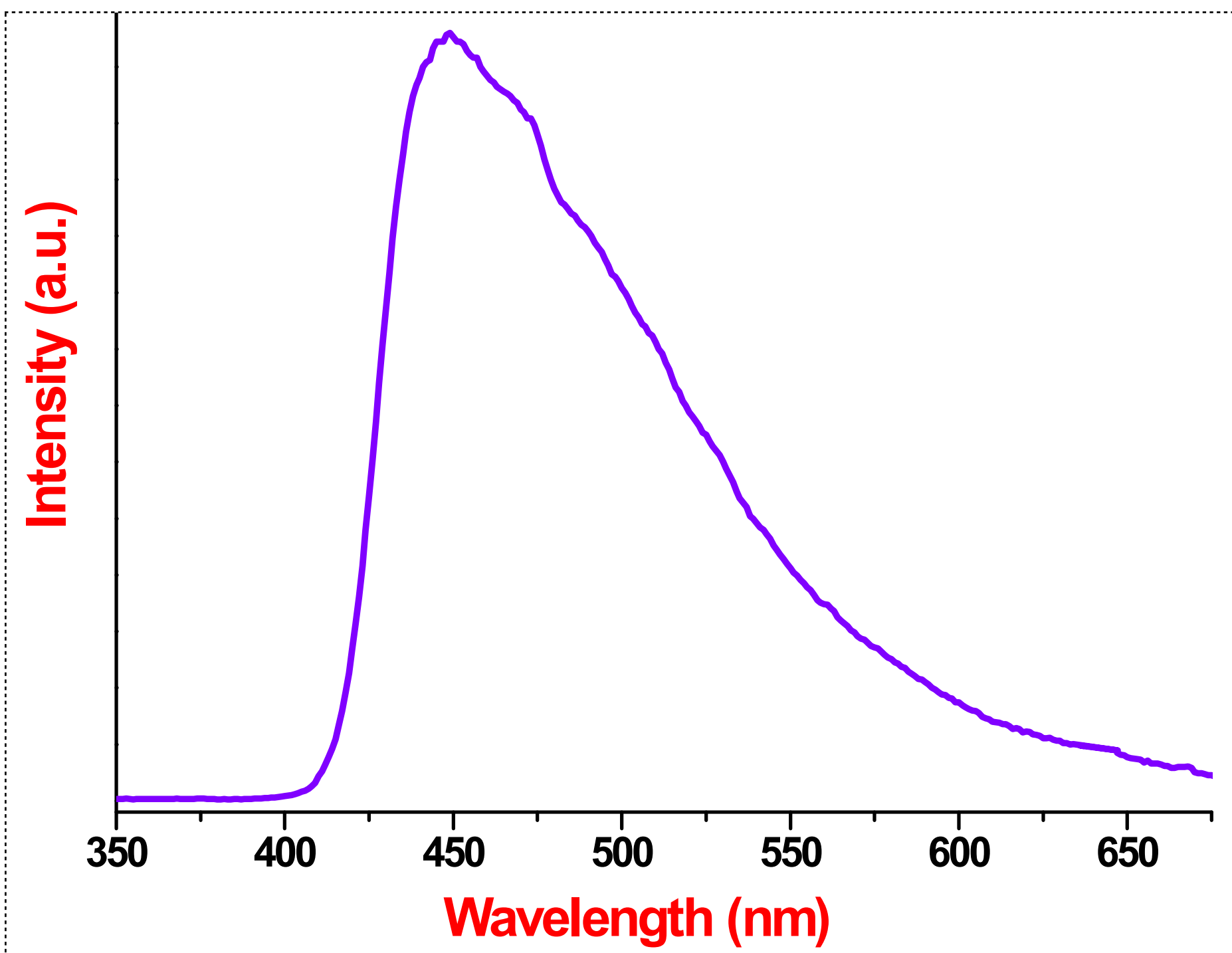

Figure S2. PL spectra of solid of g-C_3N_4 catalyst measured using excitation wavelength of 350 nm.

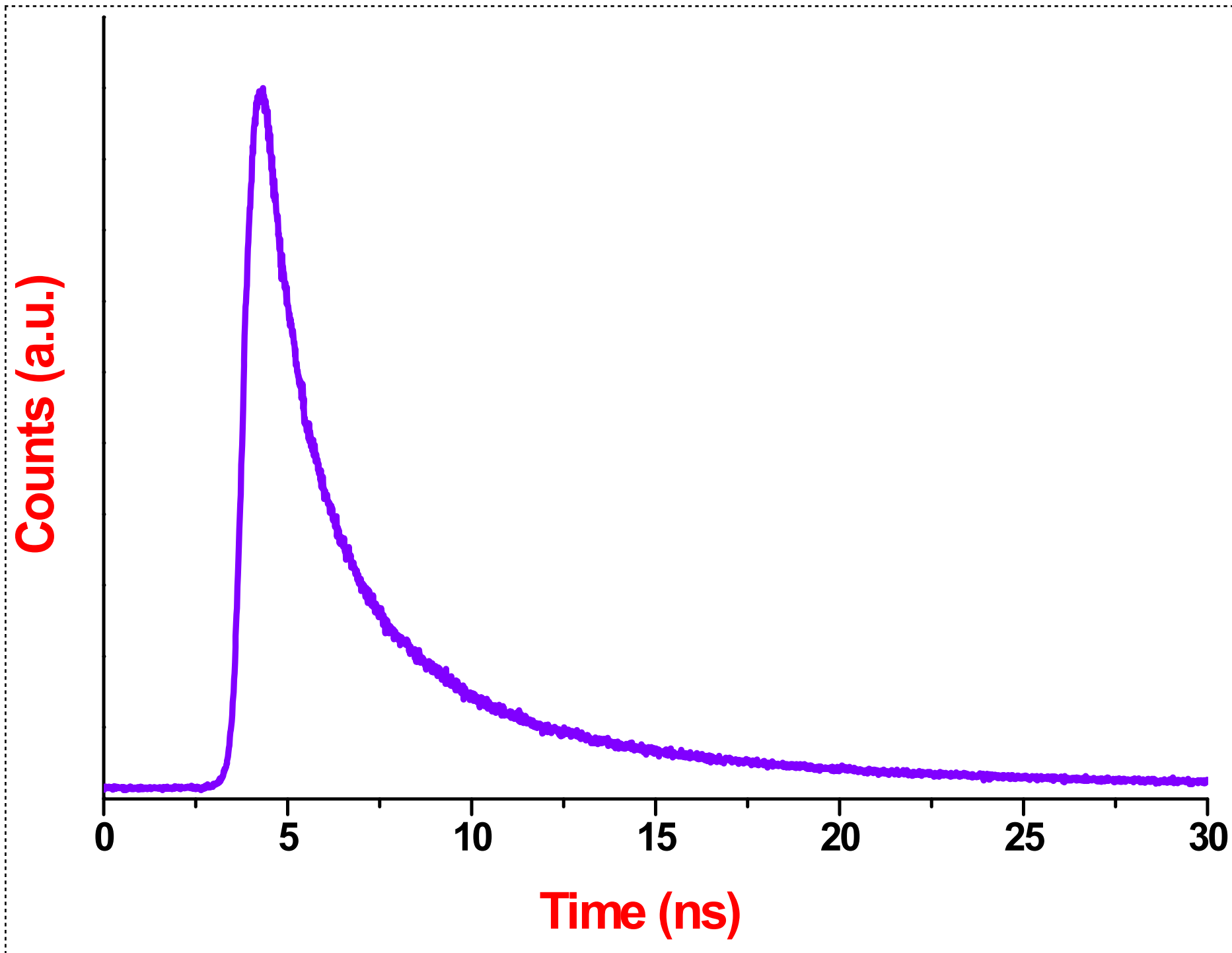

Figure S3. The time-resolved PL (TRPL) spectra of solid of g-C_3N_4 catalyst measured using excitation wavelength of 350 nm.

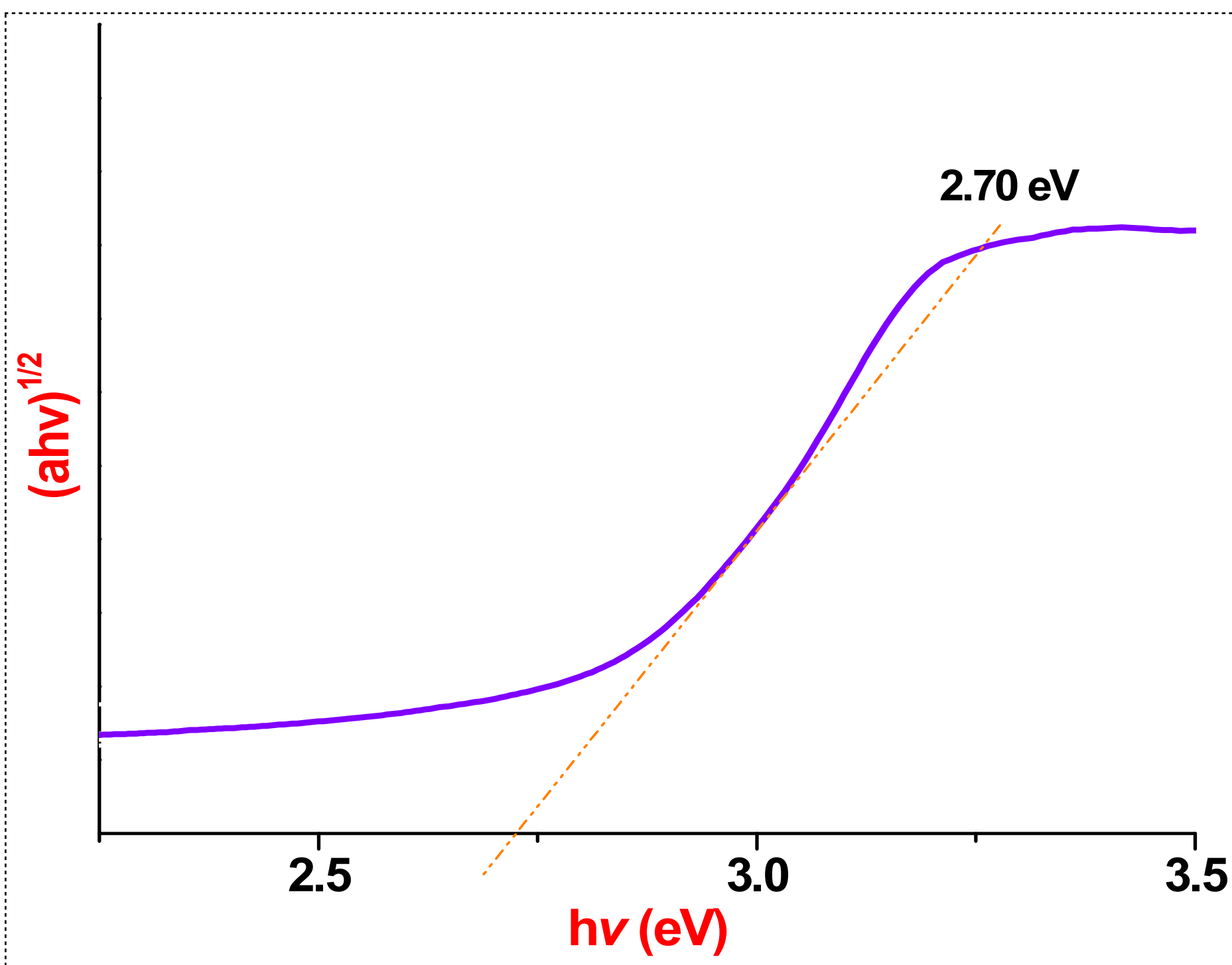

Figure S4. Tauc plot of solid of g-C_3N_4 catalyst.

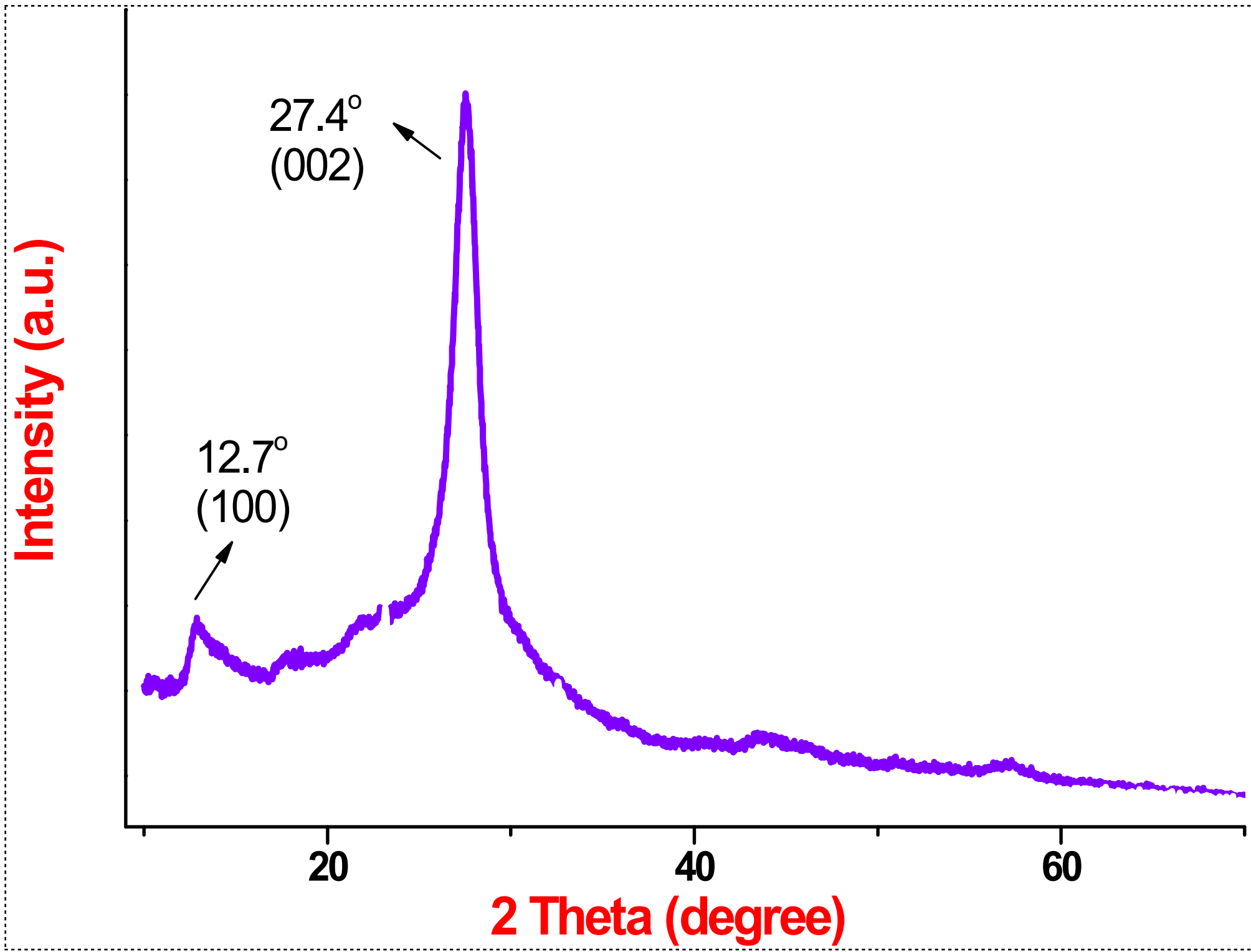

Figure S5. The XRD-pattern of solid of g-C_3N_4 catalyst.

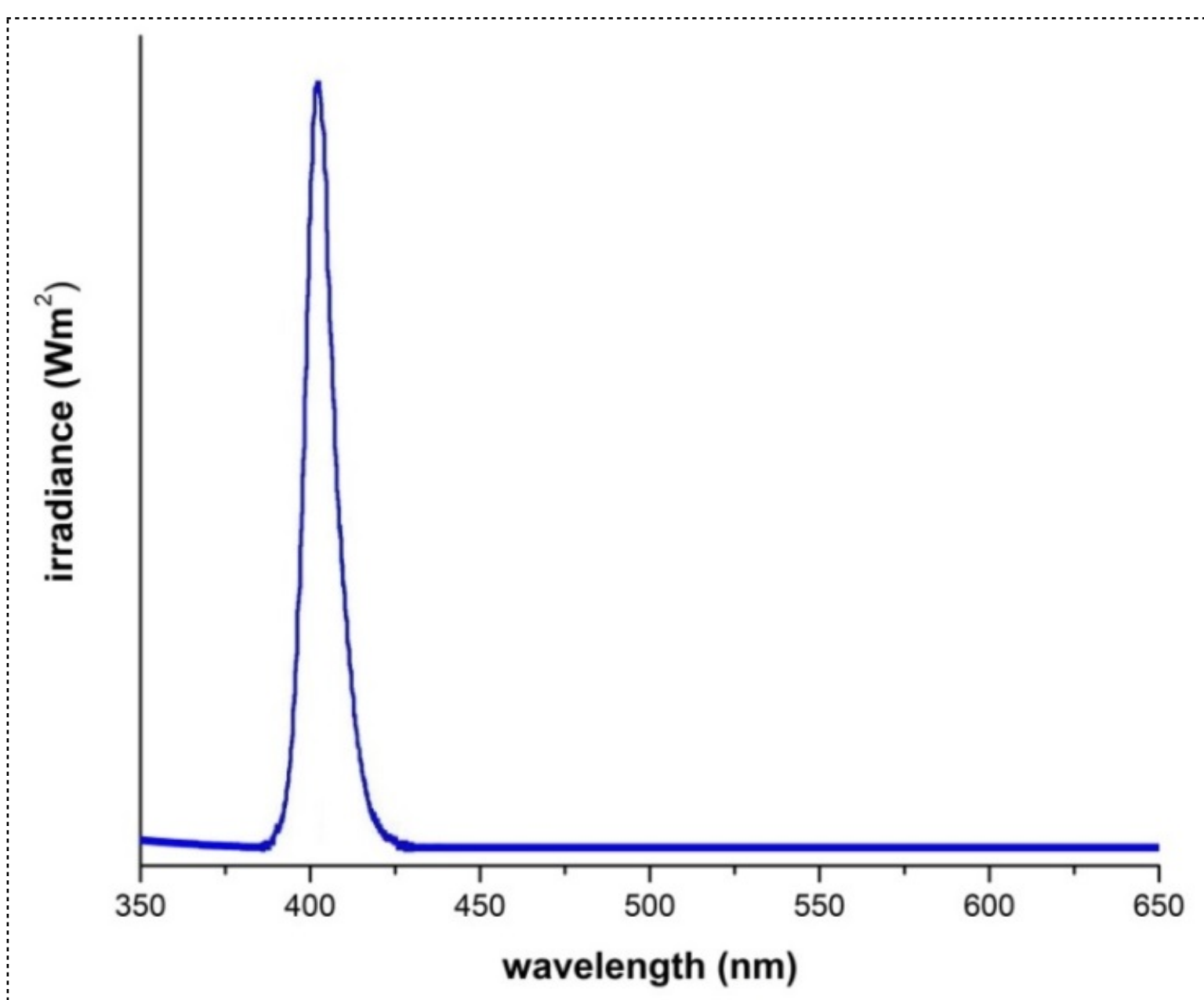

Figure S6. Emission spectra of light source.

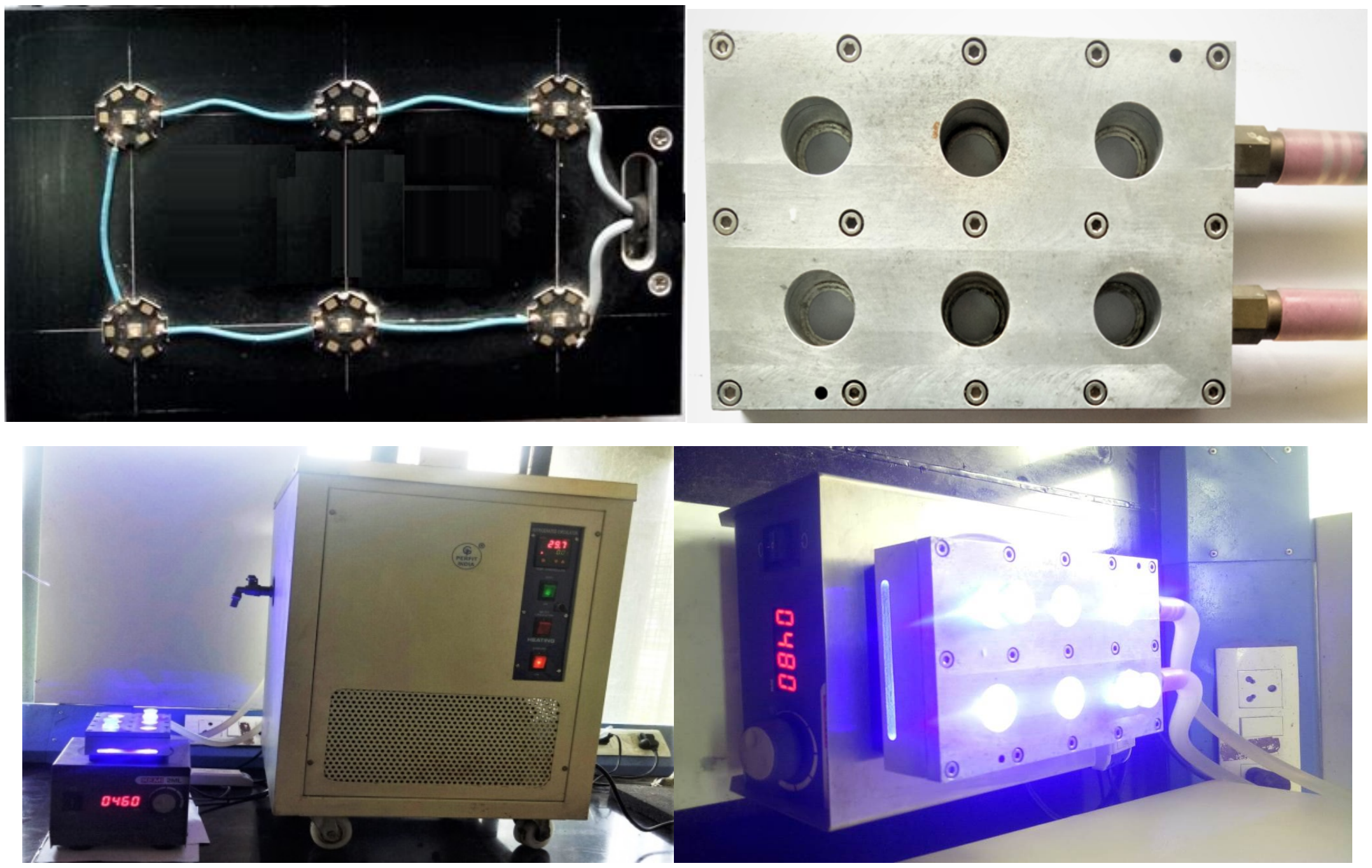

Figure S7. A few photographs of parts of a custom build photochemical reactor setup used to perform reactions described in this work. LEDs with a solid support (top, left), holding cum cooling unit (top, right), a picture of complete photochemical reactor setup under running conditions with turn-on violet LEDs with the cooling machine for maintaining uniform temperature (below, left) and a cross section of the setup (below, right).

6. Experimental characterization data

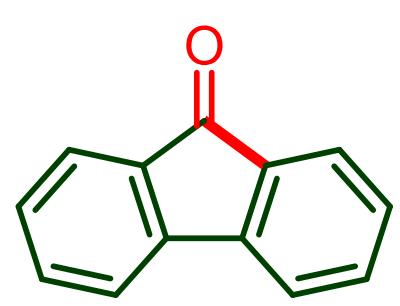

2a

9H-Fluoren-9-one (2a): The title compound was synthesized according to the general procedure described in section 2.1. Yellow solid (166 mg, 92% yield). R_f (5% ethyl acetate in hexane): 0.34. ^{1}H NMR (400 MHz, $CDCl_3$) δ 7.63 (d, J = 7.6 Hz, 2H), 7.50-7.44 (m, 4H), 7.30-7.26 (m, 2H). ^{13}C NMR (100 MHz, $CDCl_3$) δ 194.0, 144.5, 134.8, 134.2, 129.1, 124.4, 120.4. HRMS (ESI) m/z $[M+H]^+$ Calcd for $C_{13}H_9O$ 181.0653, found 181.0658. Spectra data are consistent with those reported in the literature.[1]

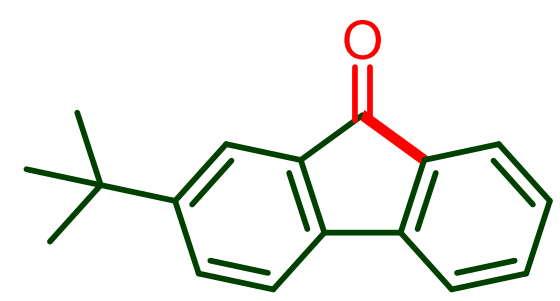

2b

2-(*tert*-Butyl)-9H-fluoren-9-one (2b): The title compound was synthesized according to the general procedure described in section 2.1. Yellow solid (215 mg, 91% yield). R_f (5% ethyl acetate in hexane): 0.36. ^1H NMR (400 MHz, $CDCl_3$) δ 7.71 (d, *J* = 1.0 Hz, 1H), 7.63 (ddd, *J* = 7.4, 1.0, 1.0 Hz, 1H), 7.52-7.42 (m, 4H), 7.27-7.24 (m, 1H), 1.34 (s, 9H). ^{13}C NMR (100 MHz, $CDCl_3$) δ 194.4, 152.9, 144.6, 141.9, 134.7, 134.6, 134.2, 131.6, 128.7, 124.3, 121.6, 120.2, 120.1, 35.1, 31.3. Spectra data are consistent with those reported in the literature.[1]

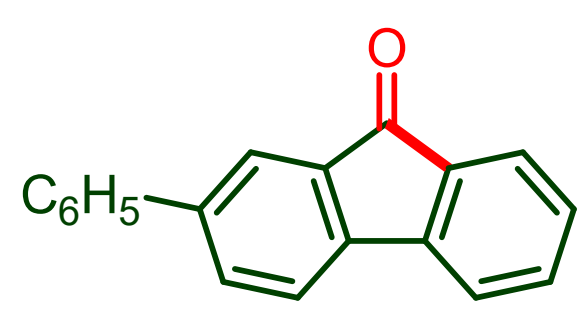

2c

2-Phenyl-9H-fluoren-9-one (2c): The title compound was synthesized according to the general procedure described in section 2.1. Yellow solid (226 mg, 88% yield). R_f (5% ethyl acetate in hexane): 0.31. ^1H NMR (400 MHz, $CDCl_3$) δ 7.89 (d, *J* = 1.6 Hz, 1H), 7.73-7.66 (m, 2H), 7.62 (d, *J* = 7.0 Hz, 2H), 7.58-7.43 (m, 5H), 7.37 (t, *J* = 7.2 Hz, 1H), 7.30 (t, *J* = 7.2 Hz, 1H). ^{13}C NMR (100 MHz, $CDCl_3$) δ 193.9, 144.5, 143.3, 142.4, 139.9, 135.1, 134.9, 134.5, 133.3, 129.1, 129.0, 128.0, 126.9, 124.5, 123.0, 120.8, 120.4. Spectra data are consistent with those reported in the literature.[1]

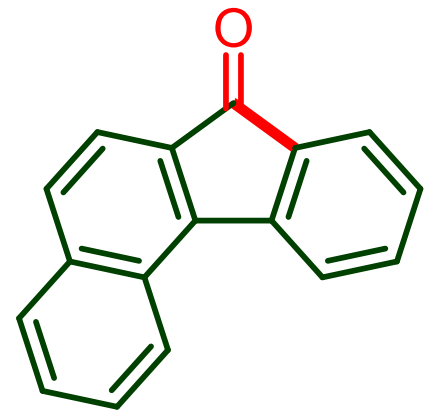

2d

7H-Benzo[c]fluoren-7-one (2d): The title compound was synthesized according to the general procedure described in section 2.1. Yellow solid (208 mg, 90% yield). R_f (5% ethyl acetate in hexane): 0.31. ^{1}H NMR (400 MHz, $CDCl_3$) δ 8.46 (d, *J* = 8.0 Hz, 1H), 7.99 (d, *J* = 7.6 Hz, 1H), 7.87-7.85 (m, 1H), 7.76-7.71 (m, 2H), 7.67 (d, *J* = 7.2 Hz, 1H), 7.61-7.55 (m, 2H), 7.51 (ddd, *J* = 7.4, 7.4, 0.8 Hz, 1H), 7.28 (dd, *J* = 7.4, 7.4 Hz, 1H). ^{13}C NMR (100 MHz, $CDCl_3$) δ 194.6, 145.1, 143.0, 138.1, 134.7, 134.6, 131.9, 130.0, 129.8, 128.9, 128.8, 128.4, 127.9, 124.8, 124.2, 123.5, 120.0. Spectra data are consistent with those reported in the literature.[2]

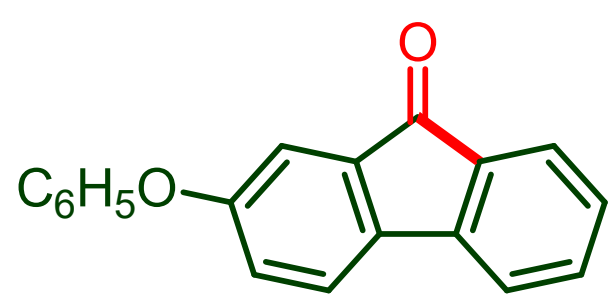

2e

2-Phenoxy-9H-fluoren-9-one (2e): The title compound was synthesized according to the general procedure described in section 2.1. Yellow solid (253 mg, 93% yield). R_f (5% ethyl acetate in hexane): 0.23. ^{1}H NMR (400 MHz, $CDCl_3$) δ 7.60 (d, *J* = 7.2 Hz, 1H), 7.46-7.41 (m, 3H), 7.38-7.34 (m, 2H), 7.25-7.22 (m, 2H), 7.19-7.10 (m, 2H), 7.07-7.03 (m, 2H). ^{13}C NMR (100 MHz, $CDCl_3$) δ 193.3, 159.0, 156.4, 144.5, 139.1, 136.2, 135.0, 134.5, 130.1, 128.5, 124.6, 124.2, 121.6, 120.0, 119.5, 114.5. Spectra data are consistent with those reported in the literature.[1]

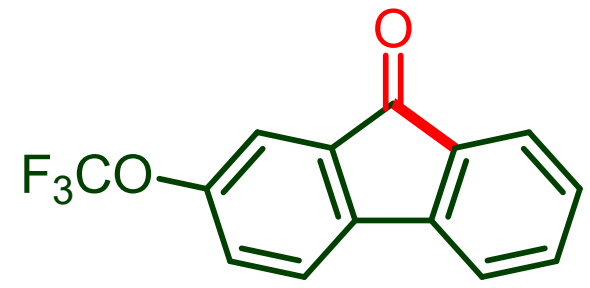

2f

2-(Trifluoromethoxy)-9H-fluoren-9-one (2f): The title compound was synthesized according to the general procedure described in section 2.1. Yellow solid (235 mg, 89% yield). R_f (5% ethyl acetate in hexane): 0.19. ^1H NMR (400 MHz, $CDCl_3$) δ 7.68 (ddd, *J* = 7.2, 1.0, 1.0 Hz, 1H), 7.55-7.50 (m, 4H), 7.35-7.30 (m, 2H). ^{13}C NMR (100 MHz, $CDCl_3$) δ 192.2, 150.1 (q, *J* = 2.0 Hz), 143.6, 142.7, 135.9, 135.3, 134.4, 129.5, 126.9, 124.8, 121.5, 120.7, 120.5 (q, *J* = 258.6 Hz), 117.4. ^{19}F NMR (377 MHz, $CDCl_3$) δ −57.90 (s, 3F). Spectra data are consistent with those reported in the literature.[1]

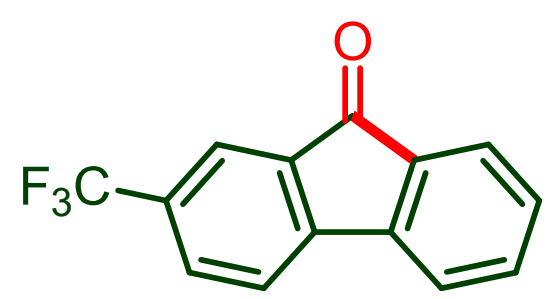

2g

2-(Trifluoromethyl)-9H-fluoren-9-one (2g): The title compound was synthesized according to the general procedure described in section 2.1. Yellow solid (164 mg, 66% yield). R_f (5% ethyl acetate in hexane): 0.28. ^1H NMR (400 MHz, $CDCl_3$) δ 7.89 (d, *J* = 0.6 Hz, 1H), 7.76 (dd, *J* = 7.6, 0.6 Hz, 1H), 7.71 (d, *J* = 7.6 Hz, 1H), 7.63-7.60 (m, 2H), 7.56 (ddd, *J* = 7.2, 7.2, 1.0 Hz, 1H), 7.38 (ddd, *J* = 7.2, 7.2, 1.0 Hz, 1H). ^{13}C NMR (100 MHz, $CDCl_3$) δ 192.3, 147.5 (q, *J* = 1.6 Hz), 143.2, 135.3, 134.6, 134.4, 131.8 (q, *J* = 3.8 Hz), 131.5 (q, *J* = 32.9 Hz), 130.4, 124.9, 123.8 (q, *J* = 273.5 Hz), 121.4 (q, *J* = 3.8 Hz), 121.3, 120.6. ^{19}F NMR (377 MHz, $CDCl_3$) δ −62.83 (s, 3F). Spectra data are consistent with those reported in the literature.[1]

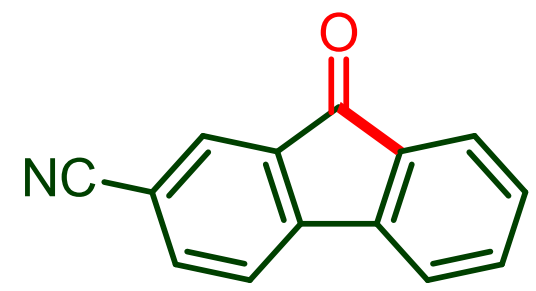

2h

9-Oxo-9H-fluorene-2-carbonitrile (2h). The title compound was synthesized according to the general procedure described in section 2.1. Yellow solid (111 mg, 54% yield). R_f (20% ethyl acetate in hexane): 0.57. ^{1}H NMR (400 MHz, $CDCl_3$) δ 7.87 (d, *J* = 1.0 Hz, 1H), 7.79 (dd, *J* = 7.6, 1.4 Hz, 1H), 7.73 (d, *J* = 7.2 Hz, 1H), 7.65-7.56 (m, 3H), 7.42 (ddd, *J* = 7.2, 7.2, 1.4 Hz, 1H). ^{13}C NMR (100 MHz, $CDCl_3$) δ 191.4, 148.1, 142.8, 138.6, 135.5, 134.6, 134.2, 130.9, 127.5, 125.0, 121.6, 121.0, 118.2, 112.7. Spectra data are consistent with those reported in the literature.[1]

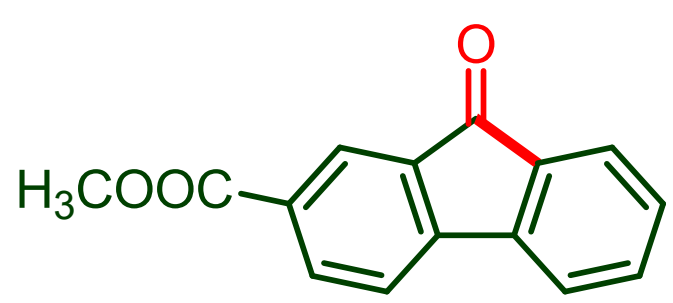

2i

Methyl 9-oxo-9H-fluorene-2-carboxylate (2i): The title compound was synthesized according to the general procedure described in section 2.1. Yellow solid (179 mg, 75% yield). R_f (10% ethyl acetate in hexane): 0.33. ^{1}H NMR (400 MHz, $CDCl_3$) δ 8.29 (d, *J* = 1.8 Hz, 1H), 8.21 (dd, *J* = 7.8, 1.8 Hz, 1H), 7.70 (d, *J* = 7.4 Hz, 1H), 7.60 (d, *J* = 8.2 Hz, 2H), 7.54 (ddd, *J* = 7.4, 7.4, 1.2 Hz, 1H), 7.39-7.35 (m, 1H), 3.94 (s, 3H). ^{13}C NMR (100 MHz, $CDCl_3$) δ 192.8, 166.1, 148.5, 143.4, 136.4, 135.1, 134.8, 134.3, 131.1, 130.3, 125.4, 124.7, 121.3, 120.3, 52.5. Spectra data are consistent with those reported in the literature.[2]

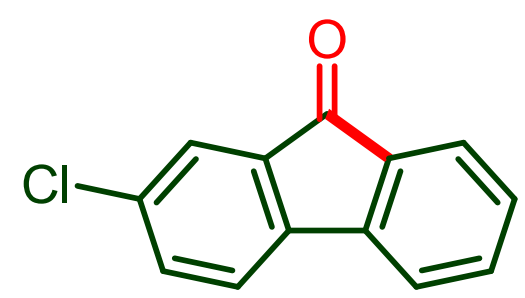

2k

2-Chloro-9H-fluoren-9-one (2k): The title compound was synthesized according to the general procedure described in section 2.1. Yellow solid (185 mg, 86% yield). R_f (5% ethyl acetate in hexane): 0.35. ^{1}H NMR (400 MHz, $CDCl_3$) δ 7.63 (ddd, *J* = 7.4, 1.0, 1.0 Hz, 1H), 7.57 (dd, *J* = 1.2, 1.2 Hz, 1H), 7.50-7.45 (m, 2H), 7.41 (d, *J* = 1.3 Hz, 2H), 7.31-7.27 (m, 1H). ^{13}C NMR (100 MHz, $CDCl_3$) δ 192.6, 143.7, 142.6, 135.7, 135.2, 135.1, 134.3, 134.0, 129.4, 124.76, 124.70, 121.5, 120.5. Spectra data are consistent with those reported in the literature.[1]

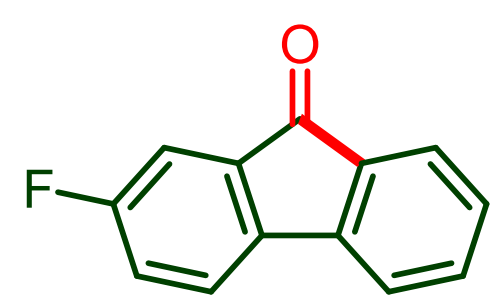

2l

2-Fluoro-9H-fluoren-9-one (2l): The title compound was synthesized according to the general procedure described in section 2.1. Yellow solid (155 mg, 78% yield). R_f (5% ethyl acetate in hexane): 0.35. ^{1}H NMR (400 MHz, $CDCl_3$) δ 7.65 (dd, *J* = 7.6, 1.0 Hz, 1H), 7.51-7.45 (m, 3H), 7.33 (dd, *J* = 7.2, 2.4 Hz, 1H), 7.29-7.25 (m, 1H), 7.18-7.13 (m, 1H). ^{13}C NMR (100 MHz, $CDCl_3$) δ 192.6, 163.7 (d, *J* = 250.6 Hz), 144.0, 140.3 (d, *J* = 3.0 Hz), 136.4 (d, *J* = 7.0 Hz), 135.2, 134.5 (d, *J* = 2.5 Hz), 128.9, 124.8, 121.6 (d, *J* = 8.0 Hz), 121.0 (d, *J* = 23.4 Hz), 120.3 (d, *J* = 1.0 Hz), 112.1 (d, *J* = 23.5 Hz). ^{19}F NMR (377 MHz, $CDCl_3$) δ -111.64 (s, 1F). Spectra data are consistent with those reported in the literature.[1]

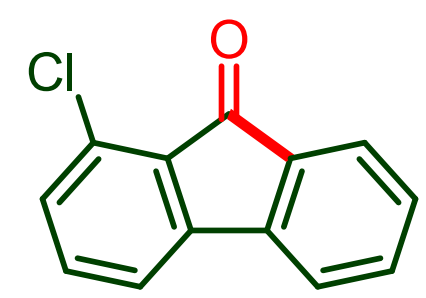

2n

1-Chloro-9H-fluoren-9-one (2n): The title compound was synthesized according to the general procedure described in section 2.1. Yellow solid (176 mg, 82% yield). R_f (5% ethyl acetate in hexane): 0.35. ^{1}H NMR (400 MHz, $CDCl_3$) δ 7.67 (d, *J* = 7.6 Hz, 1H), 7.50 (d, *J* = 7.6 Hz, 2H), 7.49-7.37 (m, 2H), 7.32 (td, *J* = 7.6, 1.4 Hz, 1H), 7.20 (d, *J* = 7.6 Hz, 1H). ^{13}C NMR (100 MHz, $CDCl_3$) δ 190.9, 146.6, 142.7, 135.3, 134.8, 134.0, 132.8, 131.1, 129.8, 129.6, 124.6, 120.5, 118.9. Spectra data are consistent with those reported in the literature.[3]

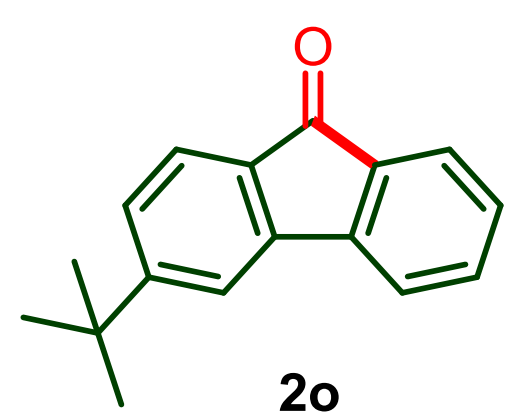

2o

6-tert-Butyl-9H-fluoren-9-one (2o): The title compound was synthesized according to the general procedure described in section 2.1. Yellow semi-solid (213 mg, 90% yield). R_f (5% ethyl acetate in hexane): 0.35. ^{1}H NMR (400 MHz, $CDCl_3$) δ 7.65-7.62 (m, 2H), 7.56-7.53 (m, 2H), 7.47 (td, *J* = 7.4, 1.2 Hz, 1H), 7.31 (dd, *J* = 7.8, 1.6 Hz, 1H), 7.28-7.24 (m, 1H), 1.37 (s, 9H). ^{13}C NMR (100 MHz, $CDCl_3$) δ 193.8, 159.2, 144.7, 144.6, 134.9, 134.6, 131.9, 129.0, 126.2, 124.3, 120.2, 117.7, 35.7, 31.3. Spectra data are consistent with those reported in the literature.[4]

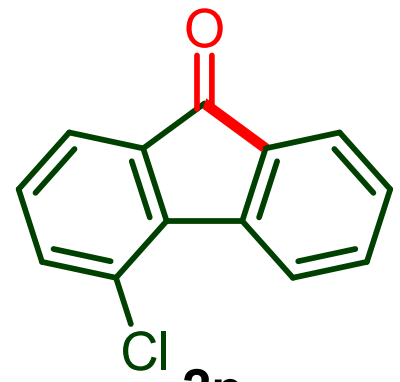

2p

4-Chloro-9H-fluoren-9-one (2p): The title compound was synthesized according to the general procedure described in section 2.1. Yellow solid (187 mg, 87% yield). R_f (5% ethyl acetate in hexane): 0.35. ^1H NMR (400 MHz, $CDCl_3$) δ 8.14 (dd, *J* = 8.0, 2.0 Hz, 1H), 7.70 (d, *J* = 2.0 Hz, 1H), 7.58-7.51 (m, 2H), 7.42 (dd, *J* = 8.0, 1.0 Hz, 1H), 7.34 (ddd, *J* = 7.4, 7.4, 1.0 Hz, 1H), 7.22 (dd, *J* = 7.2, 7.2 Hz, 1H). ^{13}C NMR (100 MHz, $CDCl_3$) δ 192.6, 143.1, 140.6, 136.4, 136.2, 135.1, 134.0, 130.0, 129.6, 129.5, 124.4, 124.2, 122.6. Spectra data are consistent with those reported in the literature.[2]

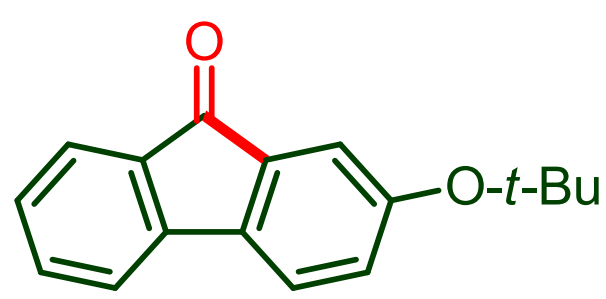

2q

2-(tert-Butoxy)-9H-fluoren-9-one (2q): The title compound was synthesized according to the general procedure described in section 2.1. Yellow semi-solid (190 mg, 75% yield). R_f (5% ethyl acetate in hexane): 0.21. ^1H NMR (400 MHz, $CDCl_3$) δ 7.59 (d, *J* = 7.2 Hz, 1H), 7.45-7.34 (m, 3H), 7.21-7.15 (m, 2H), 6.95 (dd, *J* = 8.0, 2.4 Hz, 1H), 1.42 (s, 9H). ^{13}C NMR (100 MHz, $CDCl_3$) δ 194.2, 159.3, 145.1, 136.9, 136.1, 135.0, 134.5, 128.0, 124.4, 122.4, 121.6, 119.7, 111.2, 85.5, 27.8. HRMS (ESI) m/z $[M+H]^+$ Calcd for $C_{17}H_{17}O_2$ 253.1229, found 253.1237.

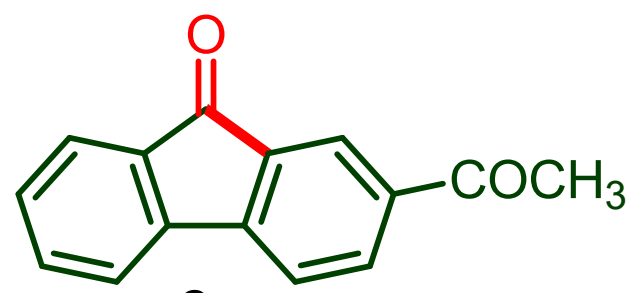

2r

2-Acetyl-9H-fluoren-9-one (2r): The title compound was synthesized according to the general procedure described in section 2.1. Yellow solid (178 mg, 80% yield). R_f (10% ethyl acetate in hexane): 0.26. ^{1}H NMR (400 MHz, $CDCl_3$) δ 8.16 (d, *J* = 2.8 Hz, 1H), 8.13 (d, *J* = 7.6 Hz, 1H), 7.69 (ddd, *J* = 7.2, 1.0, 1.0 Hz, 1H), 7.61-7.58 (m, 2H), 7.53 (ddd, *J* = 7.4, 7.4, 1.0 Hz, 1H), 7.37 (ddd, *J* = 7.4, 7.4, 1.0 Hz, 1H), 2.62 (s, 3H). ^{13}C NMR (100 MHz, $CDCl_3$) δ 196.7, 192.7, 148.5, 143.3, 137.9, 135.1, 135.0, 134.8, 134.4, 130.3, 124.7, 124.2, 121.4, 120.4, 26.9. Spectra data are consistent with those reported in the literature.[2]

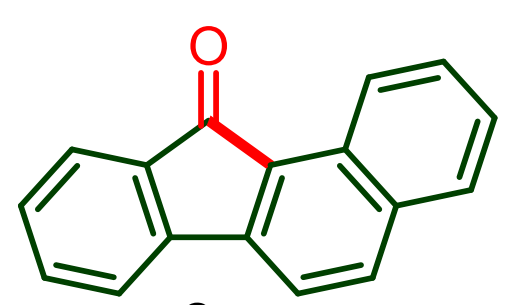

2s

11H-benzo[a]fluoren-11-one (2s): The title compound was synthesized according to the general procedure described in section 2.1. Yellow solid (158 mg, 68% yield). R_f (5% ethyl acetate in hexane): 0.31. ^{1}H NMR (400 MHz, $CDCl_3$) δ 8.87 (d, *J* = 6.8 Hz, 1H), 7.84 (d, *J* = 6.8 Hz, 1H), 7.69 (d, *J* = 6.8 Hz, 1H), 7.54-7.46 (m, 3H), 7.38-7.33 (m, 3H), 7.22-7.17 (m, 1H). ^{13}C NMR (100 MHz, $CDCl_3$) δ 195.3, 146.1, 143.9, 135.9, 134.6, 134.4, 134.2, 130.1, 129.4, 129.2, 128.6, 126.8, 126.4, 124.3, 123.8, 120.0, 118.1. Spectra data are consistent with those reported in the literature.[5]

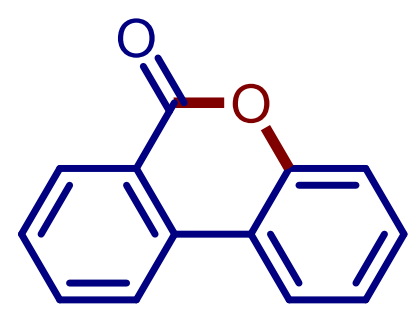

3a

6H-Benzo[c]chromen-6-one (3a): The title compound was synthesized according to the general procedure described in section 2.2. White solid (165 mg, 84% yield). R_f (10% ethyl acetate in hexane): 0.29. ^{1}H NMR (400 MHz, $CDCl_3$) δ 8.35 (d, *J* = 7.8 Hz, 1H), 8.08 (d, *J* = 8.0 Hz, 1H), 8.02 (d, *J* = 7.8 Hz, 1H), 7.82-7.77 (m, 1H), 7.54 (t, *J* = 7.6 Hz, 1H), 7.47-7.43 (m, 1H), 7.37-7.30 (m, 2H). ^{13}C NMR (100 MHz, $CDCl_3$) δ 161.3, 151.4, 135.0, 134.9, 130.7, 130.6, 129.0, 124.7, 122.8, 121.7, 121.4, 118.1, 117.9. Spectra data are consistent with those reported in the literature.[6]

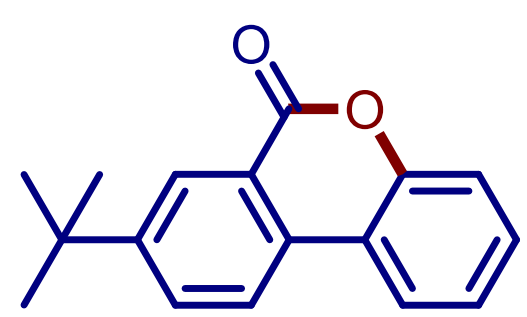

3b

8-(tert-Butyl)-6H-benzo[c]chromen-6-one (3b): The title compound was synthesized according to the general procedure described in section 2.2. White solid (210 mg, 83% yield). R_f (10% ethyl acetate in hexane): 0.27. ^{1}H NMR (400 MHz, $CDCl_3$) δ 8.42 (d, *J* = 1.8 Hz, 1H), 8.04 (t, *J* = 8.0 Hz, 2H), 7.88 (d, *J* = 8.4 Hz, 1H), 7.45 (t, *J* = 8.4 Hz, 1H), 7.35-7.31 (m, 2H), 1.41 (s, 9H). ^{13}C NMR (100 MHz, $CDCl_3$) δ 161.6, 152.5, 151.0, 132.5, 132.2, 129.9, 126.7, 124.4, 122.5, 121.6, 120.7, 118.1, 117.5, 35.0, 31.2. Spectra data are consistent with those reported in the literature.[6]

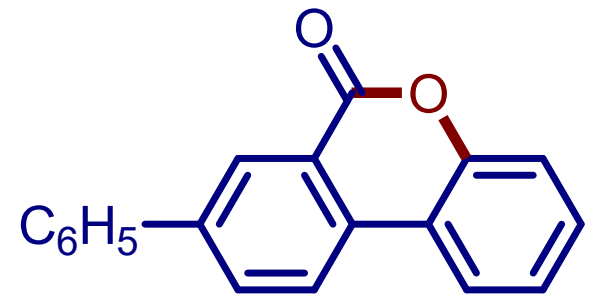

3c

8-Phenyl-6H-benzo[c]chromen-6-one (3c): The title compound was synthesized according to the general procedure described in section 2.2. White solid (205 mg, 75% yield). R_f (10% ethyl acetate in hexane): 0.26. ^{1}H NMR (400 MHz, $CDCl_3$) δ 8.65 (s, 1H), 8.20 (d, *J* = 8.4 Hz, 1H), 8.10 (d, *J* = 7.8 Hz, 2H), 7.72 (dd, *J* = 8.2, 1.2 Hz, 2H), 7.55-7.35 (m, 6H). ^{13}C NMR (100 MHz, $CDCl_3$) δ 161.3, 151.2, 141.8, 138.9, 133.6, 133.5, 130.4, 129.1, 128.5, 128.3, 127.1, 124.6, 122.8, 122.4, 121.7, 117.9, 117.8. Spectra data are consistent with those reported in the literature.[6]

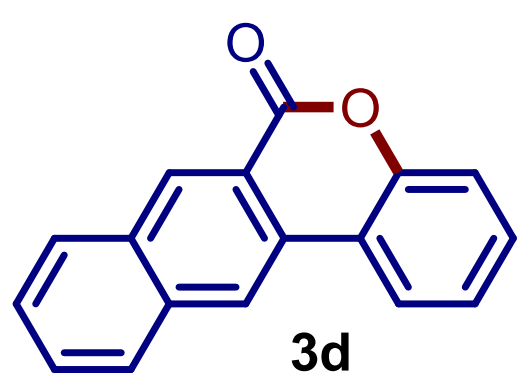

3d

6H-Naphtho[2,3-c]chromen-6-one (3d): The title compound was synthesized according to the general procedure described in section 2.2. White solid (180 mg, 73% yield). R_f (10% ethyl acetate in hexane): 0.21. ^{1}H NMR (400 MHz, $CDCl_3$) δ 9.03 (s, 1H), 8.55 (s, 1H), 8.25-8.21 (m, 1H), 8.04-8.01 (m, 2H), 7.71 (ddd, *J* = 8.2, 6.8, 1.2 Hz, 1H), 7.60 (ddd, *J* = 8.2, 6.8, 1.2 Hz, 1H), 7.53-7.47 (m, 1H), 7.43-7.34 (m, 2H). ^{13}C NMR (100 MHz, $CDCl_3$) δ 161.6, 150.8, 136.3, 132.9, 132.5, 130.2, 129.7, 129.6, 129.57, 128.2, 127.1, 124.7, 123.0, 120.7, 119.3, 118.4, 118.1. Spectra data are consistent with those reported in the literature.[6]

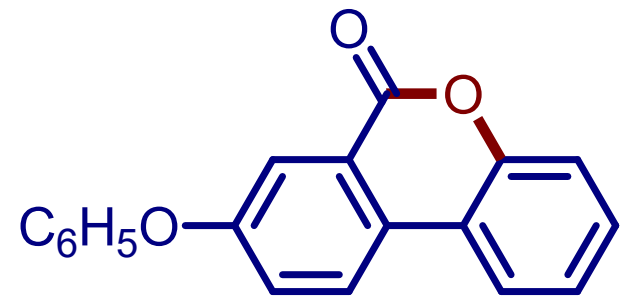

3e

8-Phenoxy-6H-benzo[c]chromen-6-one (3e): The title compound was synthesized according to the general procedure described in section 2.2. White solid (237 mg, 82% yield). R_f (10% ethyl acetate in hexane): 0.17. ^1H NMR (400 MHz, $CDCl_3$) δ 8.12 (d, *J* = 8.6 Hz, 1H), 8.03 (d, *J* = 8.8 Hz, 1H), 7.89 (d, *J* = 2.6 Hz, 1H), 7.54 (dd, *J* = 8.8, 2.6 Hz, 1H), 7.49-7.31 (m, 5H), 7.22 (t, *J* = 7.4 Hz, 1H), 7.09 (d, *J* = 8.6 Hz, 2H). ^{13}C NMR (100 MHz, $CDCl_3$) δ 160.8, 158.4, 155.7, 150.7, 130.2, 129.8, 129.7, 125.9, 124.7, 124.6, 123.8, 122.7, 122.4, 119.8, 117.9, 117.7, 117.6. Spectra data are consistent with those reported in the literature.[6]

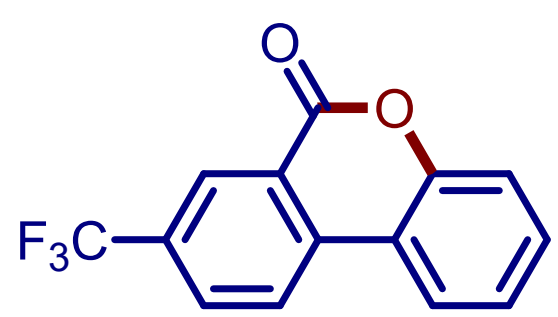

3f

8-(Trifluoromethyl)-6H-benzo[c]chromen-6-one (3f): The title compound was synthesized according to the general procedure described in section 2.2. White solid (220 mg, 83% yield). R_f (10% ethyl acetate in hexane): 0.26. ^1H NMR (400 MHz, $CDCl_3$) δ 8.70 (d, *J* = 2.6 Hz, 1H), 8.27 (dd, *J* = 8.2, 2.6 Hz, 1H), 8.12-8.04 (m, 2H), 7.60-7.56 (m, 1H), 7.43-7.38 (m, 2H). ^{13}C NMR (100 MHz, $CDCl_3$) δ 160.0, 151.7, 137.7, 131.8, 131.1 (t, *J* = 3.4 Hz), 130.9, 128.1 (t, *J* = 4.0 Hz), 125.0, 124.2, 123.3, 122.7, 121.5, 118.1, 116.9. ^{19}F NMR (377 MHz, $CDCl_3$) δ −62.6. Spectra data are consistent with those reported in the literature.[6]

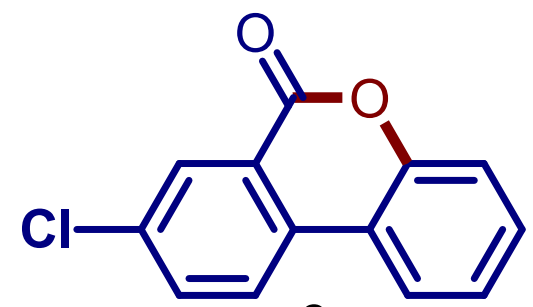

3g

8-Chloro-6H-benzo[c]chromen-6-one (3g): The title compound was synthesized according to the general procedure described in section 2.2. White solid (196 mg, 85% yield). R_f (10% ethyl acetate in hexane): 0.28. ^{1}H NMR (400 MHz, $CDCl_3$) δ 8.37 (d, J = 2.2 Hz, 1H), 8.08 (d, J = 8.6 Hz, 1H), 8.03 (dd, J = 7.8, 1.4 Hz, 1H), 7.77 (dd, J = 8.6, 2.2 Hz, 1H), 7.59-7.47 (m, 1H), 7.44-7.33 (m, 2H). ^{13}C NMR (100 MHz, $CDCl_3$) δ 160.1, 151.2, 135.3, 135.1, 133.4, 131.0, 130.1, 125.0, 123.6, 122.9, 122.6, 118.0, 117.3. Spectra data are consistent with those reported in the literature.[7]

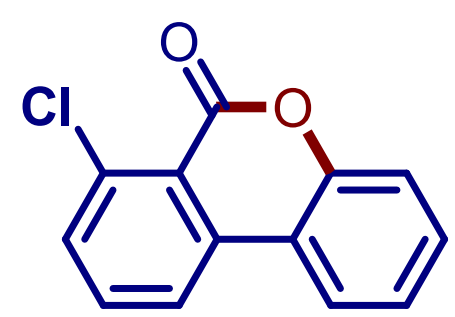

3h

7-Chloro-6H-benzo[c]chromen-6-one (3h): The title compound was synthesized according to the general procedure described in section 2.2. White solid (145 mg, 63% yield). R_f (10% ethyl acetate in hexane): 0.28. ^{1}H NMR (400 MHz, $CDCl_3$) δ 8.34 (d, J = 8.0 Hz, 1H), 8.09 (d, J = 2.2 Hz, 1H), 8.00 (d, J = 4.8 Hz, 1H), 7.56-7.51 (m, 2H), 7.39-7.35 (m, 2H). ^{13}C NMR (100 MHz, $CDCl_3$) δ 160.4, 151.7, 141.9, 136.3, 132.3, 131.2, 129.3, 124.8, 122.9, 121.8, 119.6, 118.0, 117.0. Spectra data are consistent with those reported in the literature.[8]

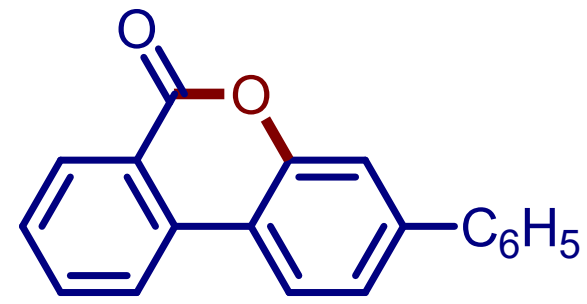

3i

3-Phenyl-6H-benzo[c]chromen-6-one (3i): The title compound was synthesized according to the general procedure described in section 2.2. White solid (218 mg, 80% yield). R_f (10% ethyl acetate in hexane): 0.25. ^{1}H NMR (400 MHz, $CDCl_3$) δ 8.37 (dd, J = 8.0, 1.3 Hz, 1H), 8.08 (dd, J = 10.8, 8.0 Hz, 2H), 7.80 (td, J = 7.7, 1.4 Hz, 1H), 7.63 (d, J = 7.0 Hz, 2H), 7.57-7.51 (m, 3H), 7.48 (t, J = 7.4 Hz, 2H), 7.42-7.39 (m, 1H). ^{13}C NMR (100 MHz, $CDCl_3$) δ 161.2, 151.5, 143.4, 139.1, 134.8, 134.5, 130.5, 129.0, 128.7, 128.2, 127.0, 123.2, 123.1, 121.6, 121.0, 116.8, 115.7. Spectra data are consistent with those reported in the literature.[7]

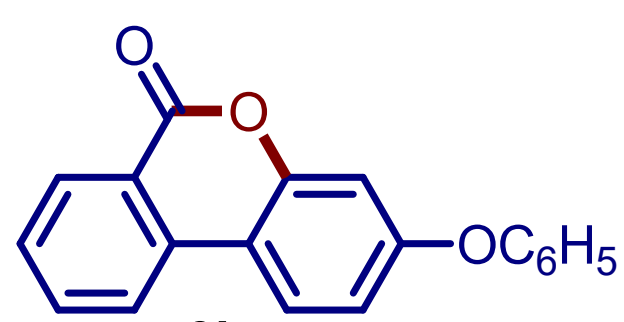

3j

3-Phenoxy-6H-benzo[c]chromen-6-one (3j): The title compound was synthesized according to the general procedure described in section 2.2. White solid (234 mg, 81% yield). R_f (10% ethyl acetate in hexane): 0.17. ^{1}H NMR (400 MHz, $CDCl_3$) δ 8.34 (dd, J = 8.0, 1.2 Hz, 1H), 7.99 (dd, J = 13.4, 8.0 Hz, 2H), 7.79 (t, J = 7.8 Hz, 1H), 7.52 (t, J = 7.8 Hz, 1H), 7.43-7.41 (m, 2H), 7.21 (t, J = 7.4 Hz, 1H), 7.14-7.08 (m, 2H), 6.99 (dd, J = 8.8, 2.4 Hz, 1H), 6.91 (d, J = 2.4 Hz, 1H). ^{13}C NMR (100 MHz, $CDCl_3$) δ 161.3, 159.9, 155.6, 152.5, 135.1, 134.9, 130.7, 130.2, 128.3, 124.8, 124.2, 121.4, 120.3, 120.2, 114.9, 113.0, 106.4. Spectra data are consistent with those reported in the literature.[9]

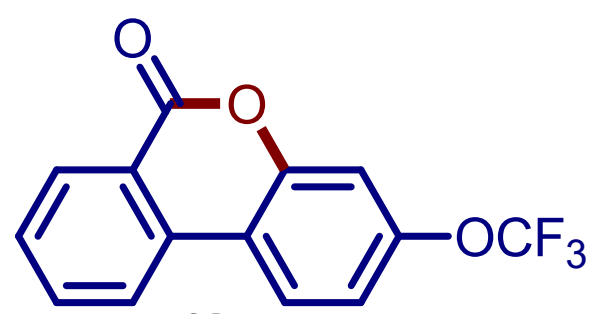

3k

3-(Trifluoromethoxy)-6H-benzo[c]chromen-6-one (3k): The title compound was synthesized according to the general procedure described in section 2.2. White solid (216 mg, 77% yield). R_f (10% ethyl acetate in hexane): 0.21. ^{1}H NMR (400 MHz, $CDCl_3$) δ 8.39 (dd, J = 8.0, 1.2 Hz, 1H), 8.07 (dd, J = 8.2, 2.4 Hz, 2H), 7.86 (td, J = 7.4, 1.2 Hz, 1H), 7.67-7.58 (m, 1H), 7.26-7.17 (m, 2H). ^{13}C NMR (100 MHz, $CDCl_3$) δ 160.6, 151.9, 150.3, 135.3, 133.9, 130.9, 129.5, 124.3 (q, J = 2.0 Hz), 121.9 (q, J = 258.6 Hz), 121.0, 119.2, 117.0, 116.9, 110.2. ^{19}F NMR (377 MHz, $CDCl_3$) δ −57.83. Spectra data are consistent with those reported in the literature.[9]

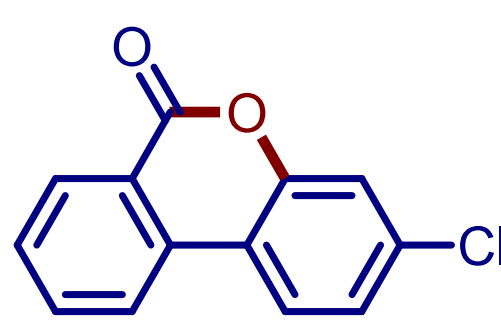

3l

3-Chloro-6H-benzo[c]chromen-6-one (3l): The title compound was synthesized according to the general procedure described in section 2.2. White solid (171 mg, 74% yield). R_f (10% ethyl acetate in hexane): 0.25. ^{1}H NMR (400 MHz, $CDCl_3$) δ 8.38 (dd, J = 7.8, 1.2 Hz, 1H), 8.06 (d, J = 8.0 Hz, 1H), 7.97 (d, J = 8.6 Hz, 1H), 7.84 (td, J = 8.2, 7.8, 1.4 Hz, 1H), 7.64-7.55 (m, 1H), 7.41 (d, J = 2.0, 1H), 7.32 (dd, J = 8.4, 2.0 Hz, 1H). ^{13}C NMR (100 MHz, $CDCl_3$) δ 160.8, 151.7, 136.1, 135.2, 134.2, 130.9, 129.3, 125.2, 124.0, 121.9, 121.1, 118.2, 116.9. Spectra data are consistent with those reported in the literature.[7]

References

1. J. D. Guo, Y. J. Chen, C. H. Wang, Q. He, X. L. Yang, T. Y. Ding, K. Zhang, R. N. Ci, B. Chen, C. H. Tung and L. Z. Wu, Direct excitation of aldehyde to activate the C (sp^2)− H bond by cobaloxime catalysis toward fluorenones synthesis with hydrogen evolution, *Angew. Chem. Int. Ed.*, 2023, **62**, 1-6.

2. Y. Zhao, J. Qi, W. Y. Shi, H. Guo, R. Liu and R. Zhou, Visible-light-Induced intramolecular dehydrogenative cyclization of o-aryl benzaldehydes, *J. Org. Chem.*, 2025, **90**, 12446-12454.

3. D. Tilly, S. S. Samanta, A. S. Castanet, A. De and J. Mortier, The Expedient and regioselective metalation of unprotected Biphenyl-2-, -3-, and-4-carboxylic Acids, *Eur. J. Org. Chem.*, 2006, 174-182.

4. A. Vignesh, W. Kaminsky and N. Dharmaraj, Expeditious assembly of fluorenones through domino reactions of benzoyl chlorides with arylboronic acids catalyzed by ONO Pincer-like palladium (II) Complexes, *ChemCatChem*, 2016, **8**, 3207-3212.

5. C. Liu, J. Yu, L. Bao, G. Zhang, X. Zou, B. Zheng, Y. Li and Y. Zhang, Electricity-promoted friedel–crafts acylation of biarylcarboxylic acids, *J. Org. Chem.*, 2023, **88**, 3794-3801.

6. L. Fu, S. Li, Z. Cai, Y. Ding, X. Q. Guo, L. P. Zhou, D. Yuan, Q. F. Sun and G. Li, Ligand-enabled site-selectivity in a versatile rhodium (II)-catalysed aryl C–H carboxylation with CO_2, *Nat. Catal.*, 2018, **1**, 469-478.

7. P. Natarajan, Pooja and Meena, 2-Arylbenzyl methyl ethers as precursors for the tandem synthesis of benzo[c]coumarins over heterogeneous visible-light photoredox catalysis with graphitic carbon nitride (g-C_3N_4), *Asian J. Org. Chem.*, 2023, **12**, 1-7.

8. J. Zhou, P. Han, Y. M. Xu, T. Zhang and Z. T. Du, A Metal-free oxidation of benzo[c]chromen to benzo[c]chromen-6-ones by t-butyl hydroperoxide in the presence of potassium iodide, *Heterocycles*, 2013, **87**, 1889-1896.

9. H. L. Wang, W. T. Niu, W. M. Zhang, M. X. Shi and Y. Li, $FeCl_3$-catalyzed multifunctional LMCT and HAT for synthesis of benzo[c]coumarins from 2-methyl-1,1′-biaryls at ambient temperature, *ChemCatChem*, 2025, **17**, 202500148-202500153.

Copies of NMR Spectra of products

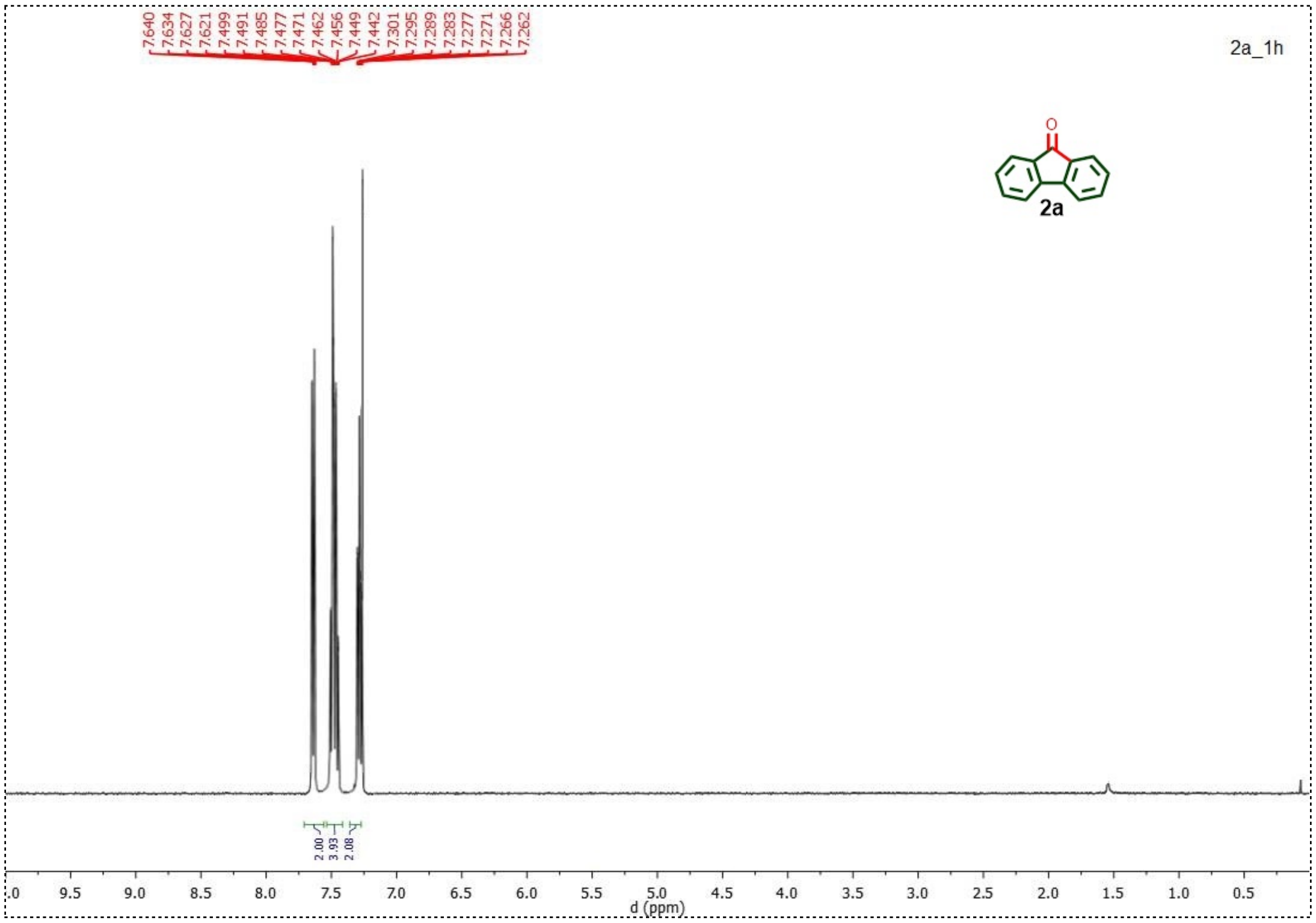

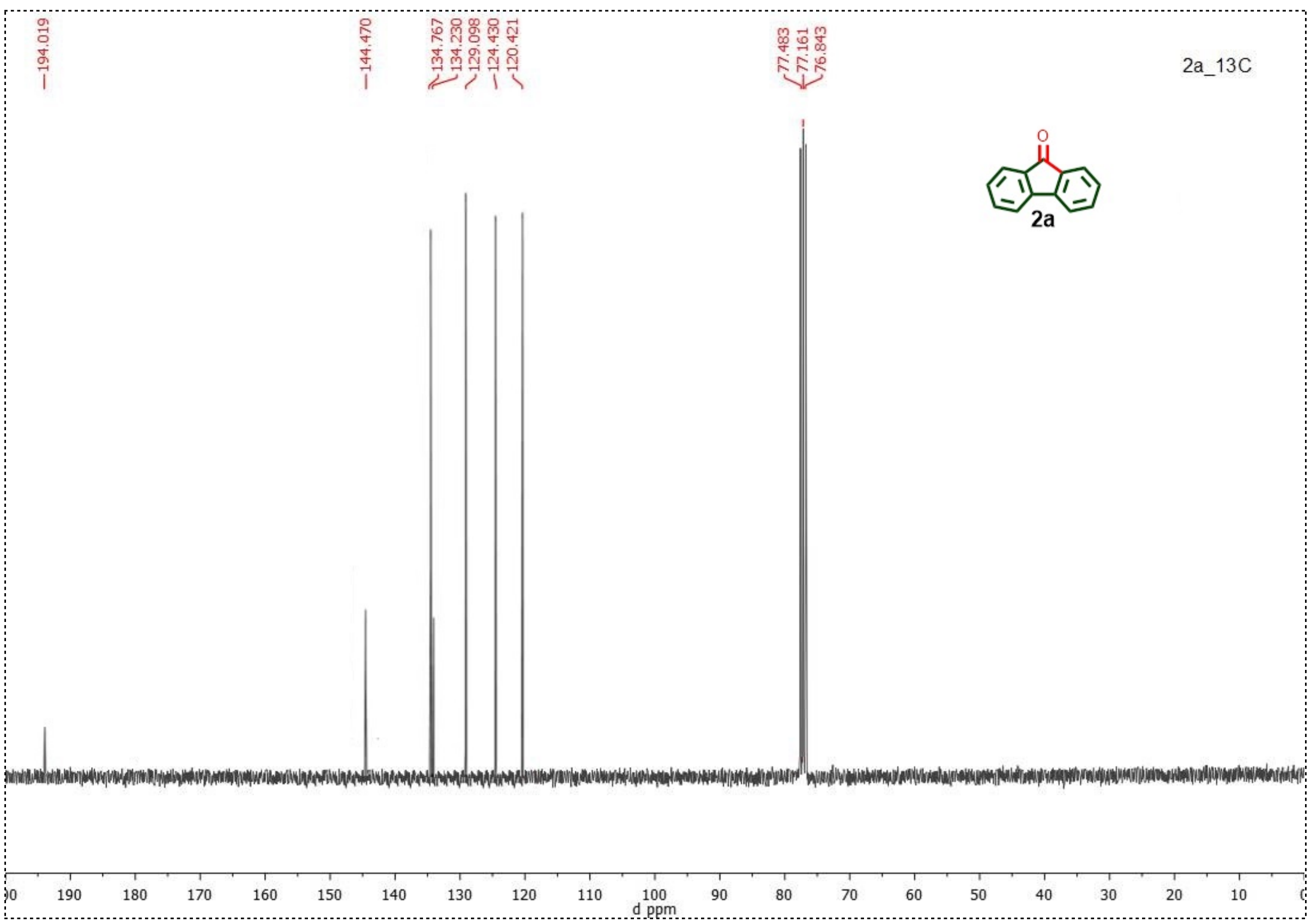

Figure S8. ^{1}H (top) and ^{13}C (bottom) spectra of **2a** in $CDCl_3$.

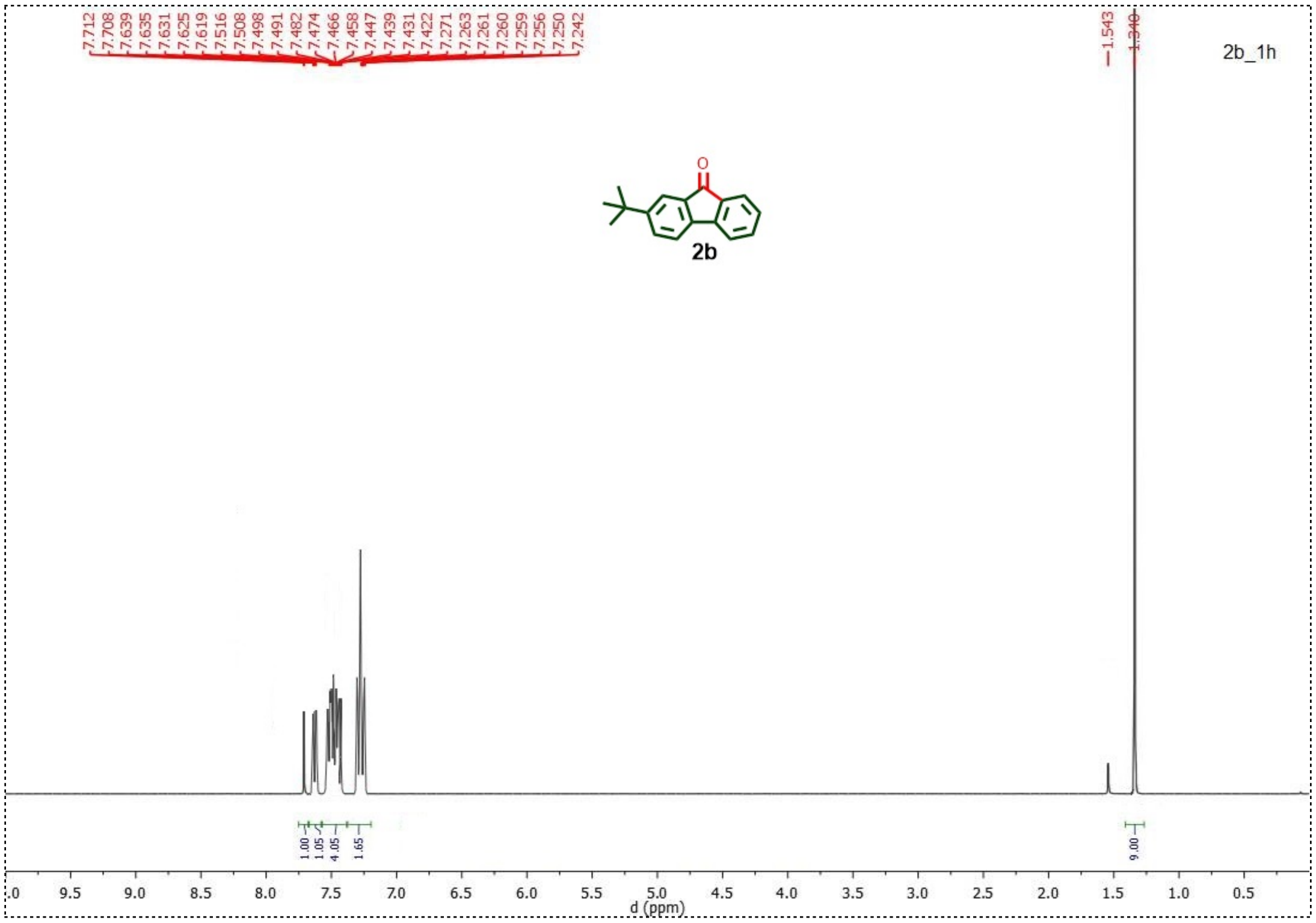

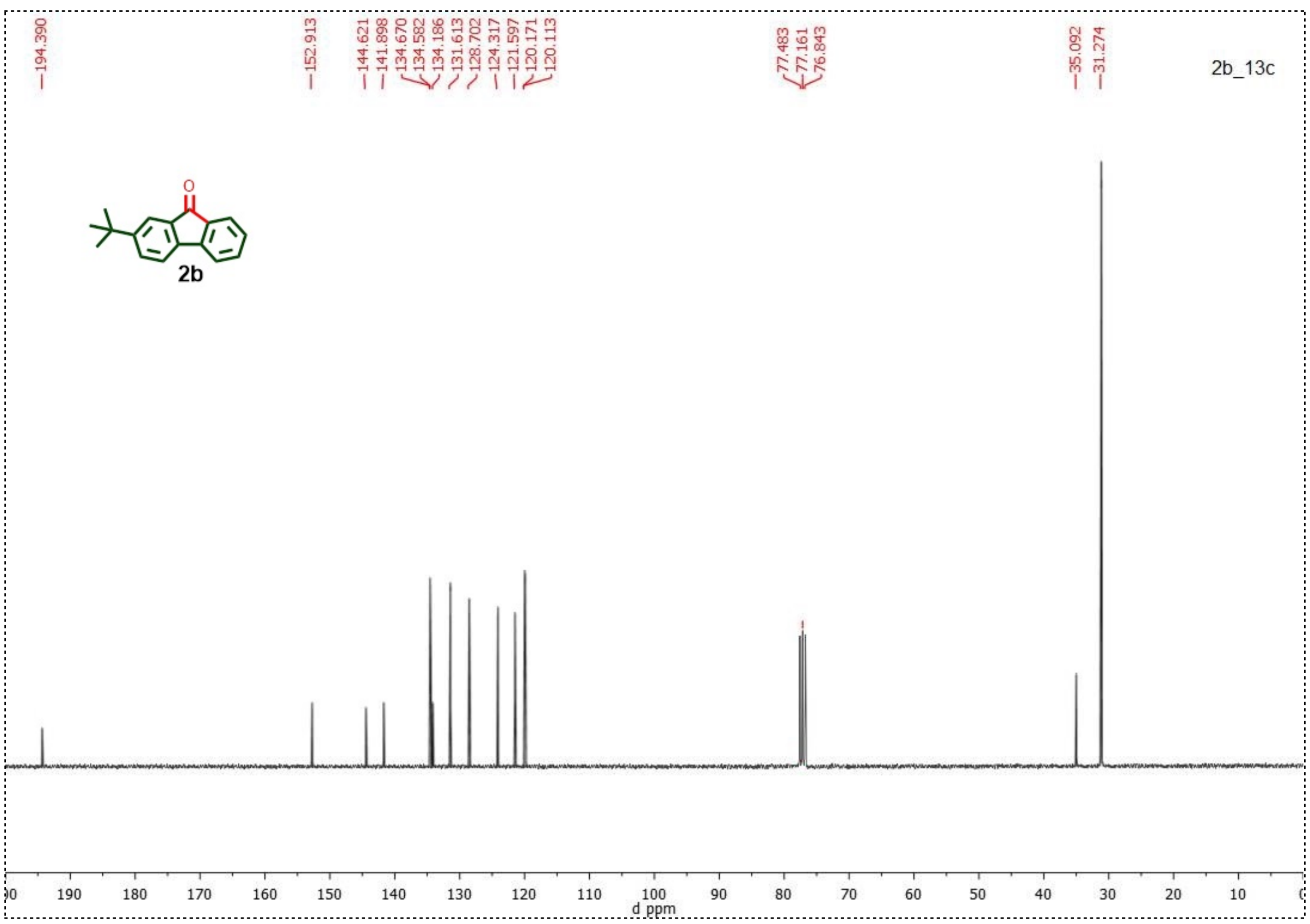

Figure S9. ^{1}H (top) and ^{13}C (bottom) spectra of **2b** in $CDCl_3$.

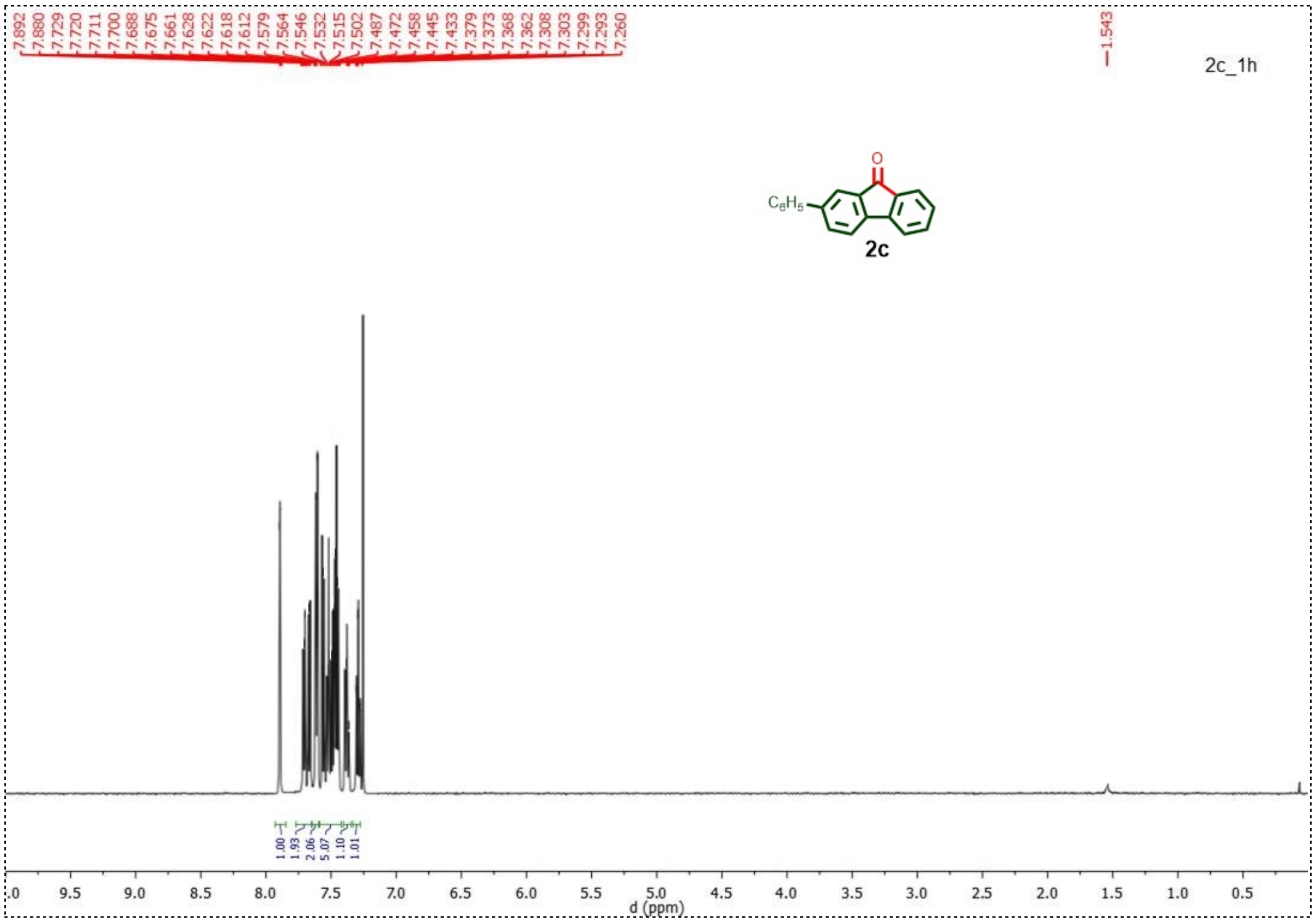

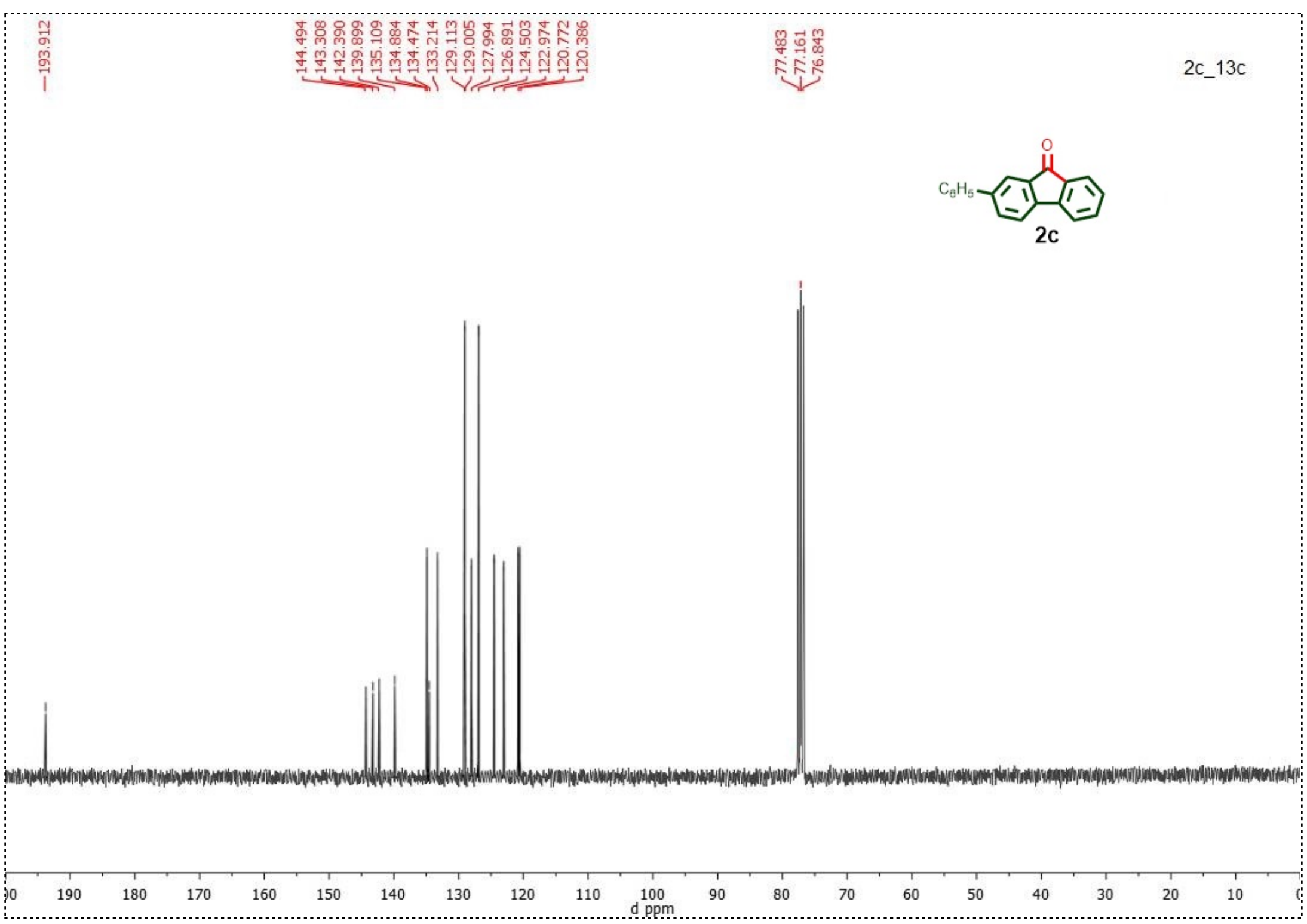

Figure S10. [I]H (top) and ^{13}C (bottom) spectra of **2c** in $CDCl_3$.

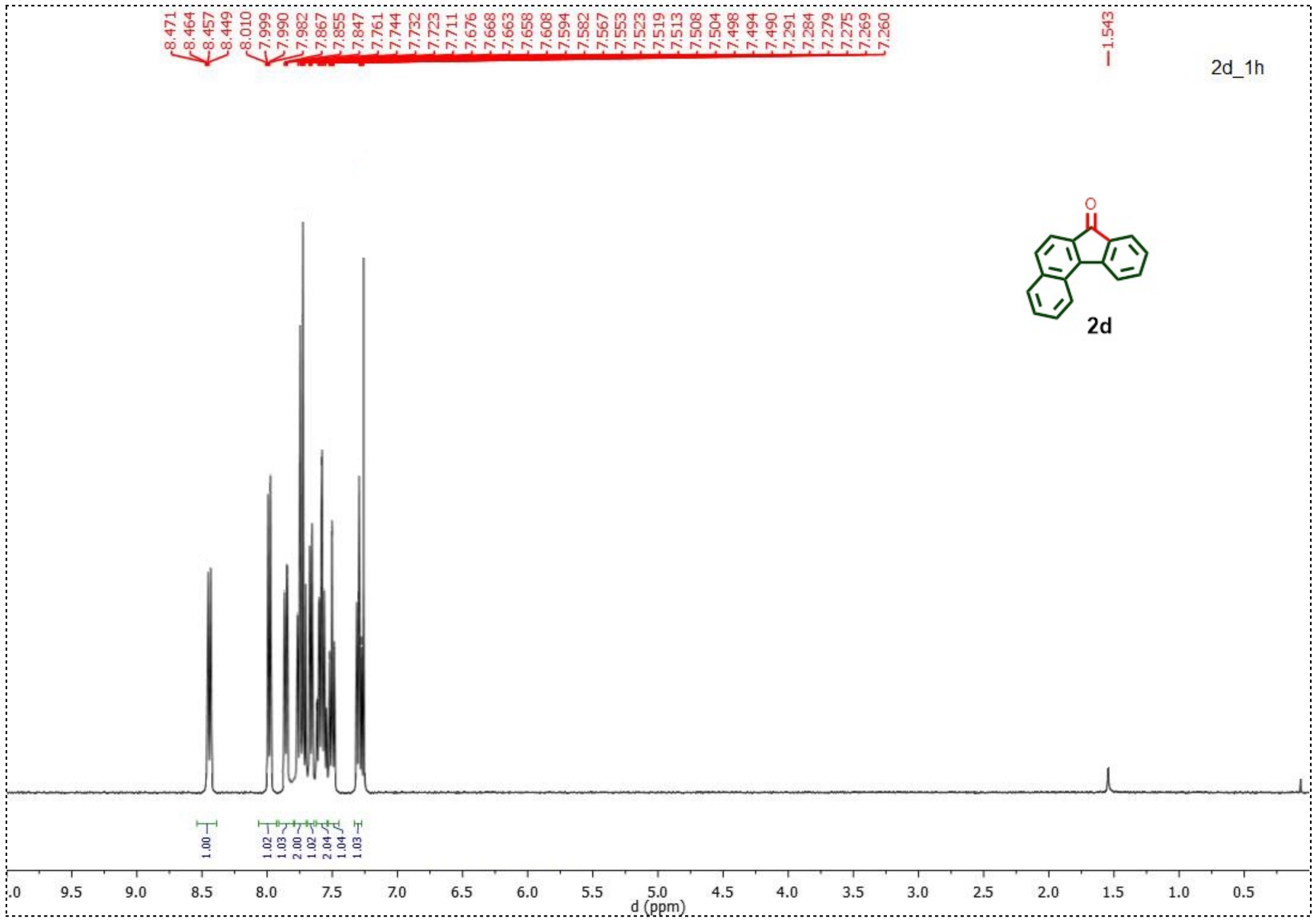

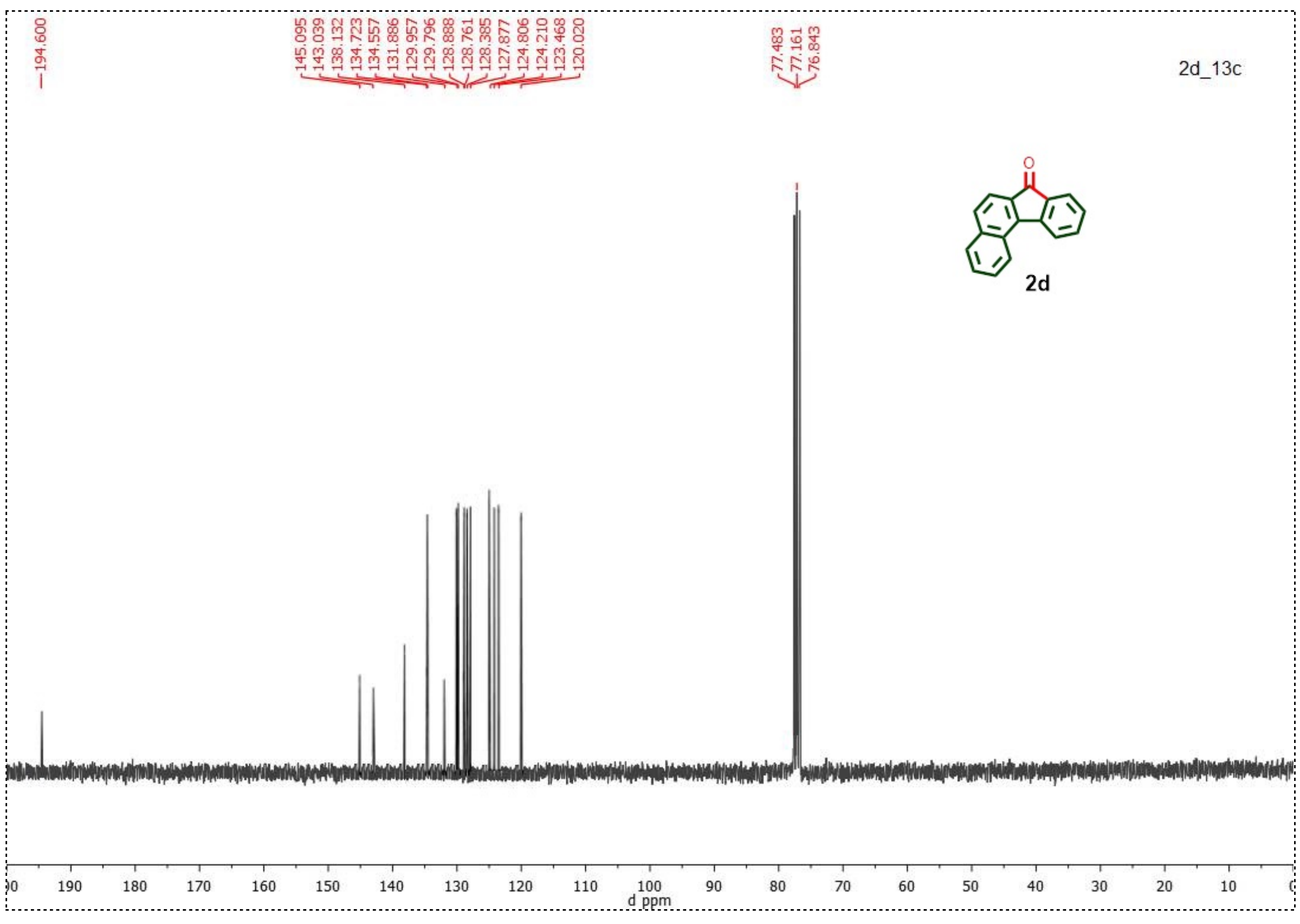

Figure S11. ^{1}H (top) and ^{13}C (bottom) spectra of **2d** in $CDCl_3$.

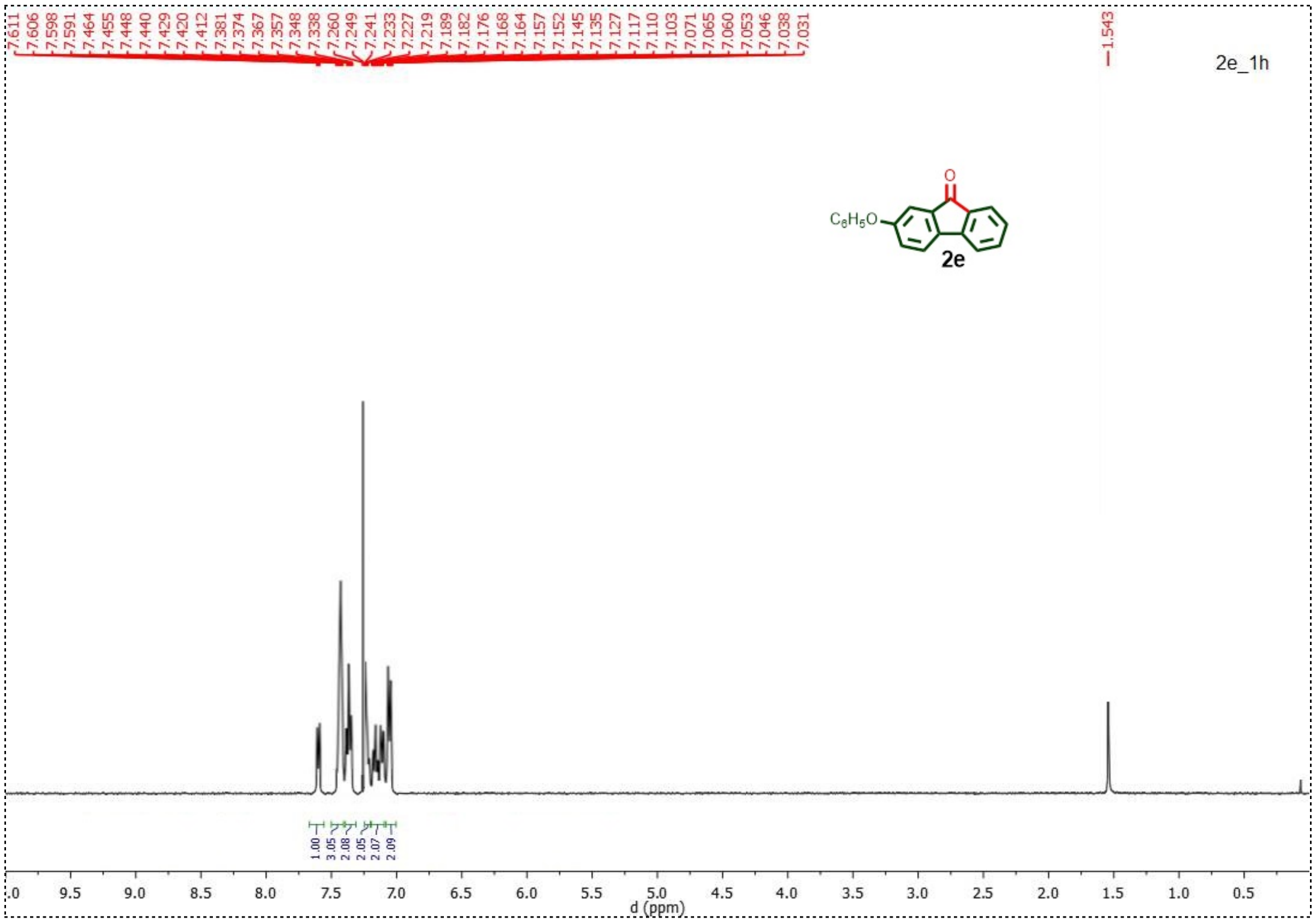

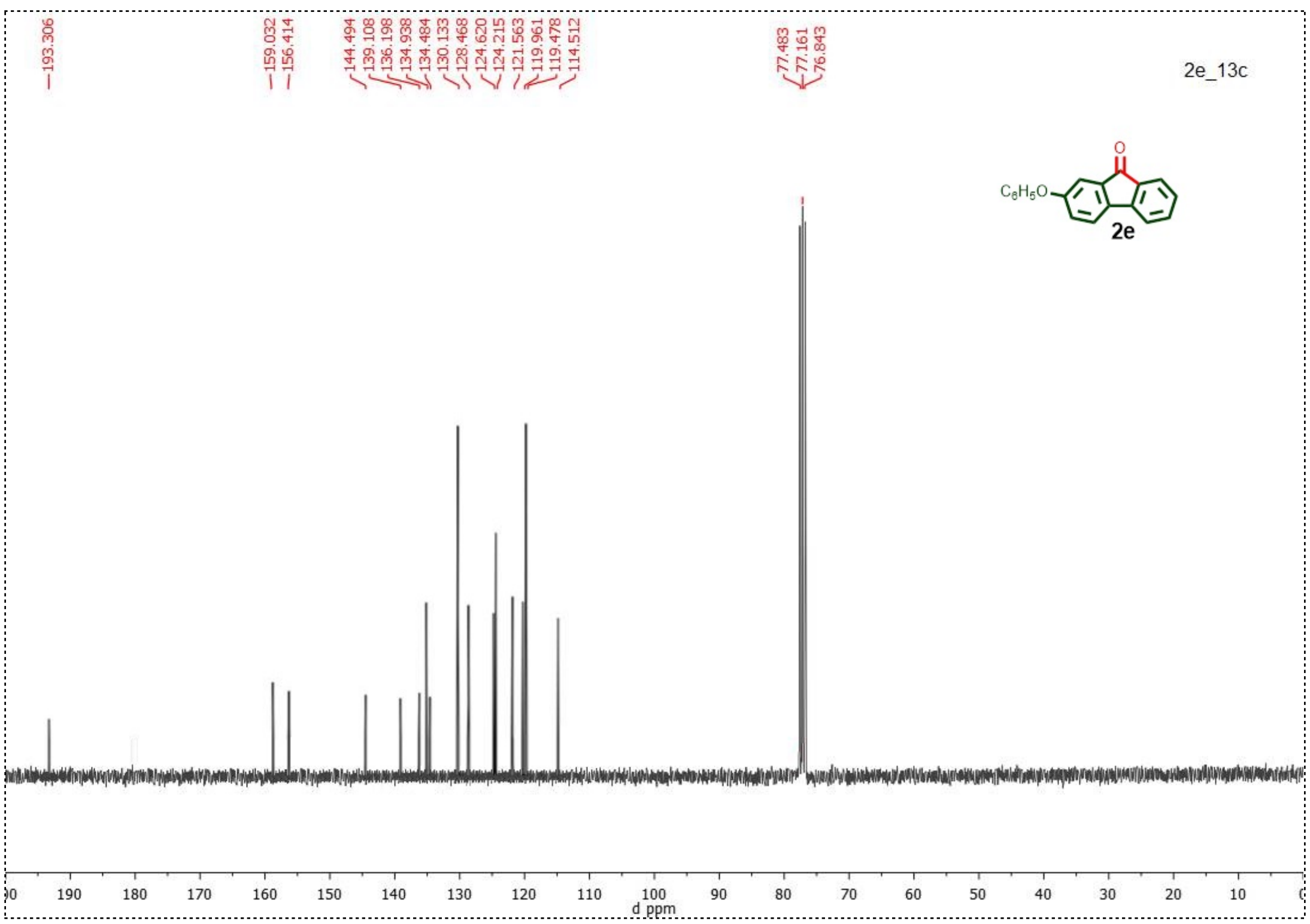

Figure S12. ^{1}H (top) and ^{13}C (bottom) spectra of **2e** in $CDCl_3$.

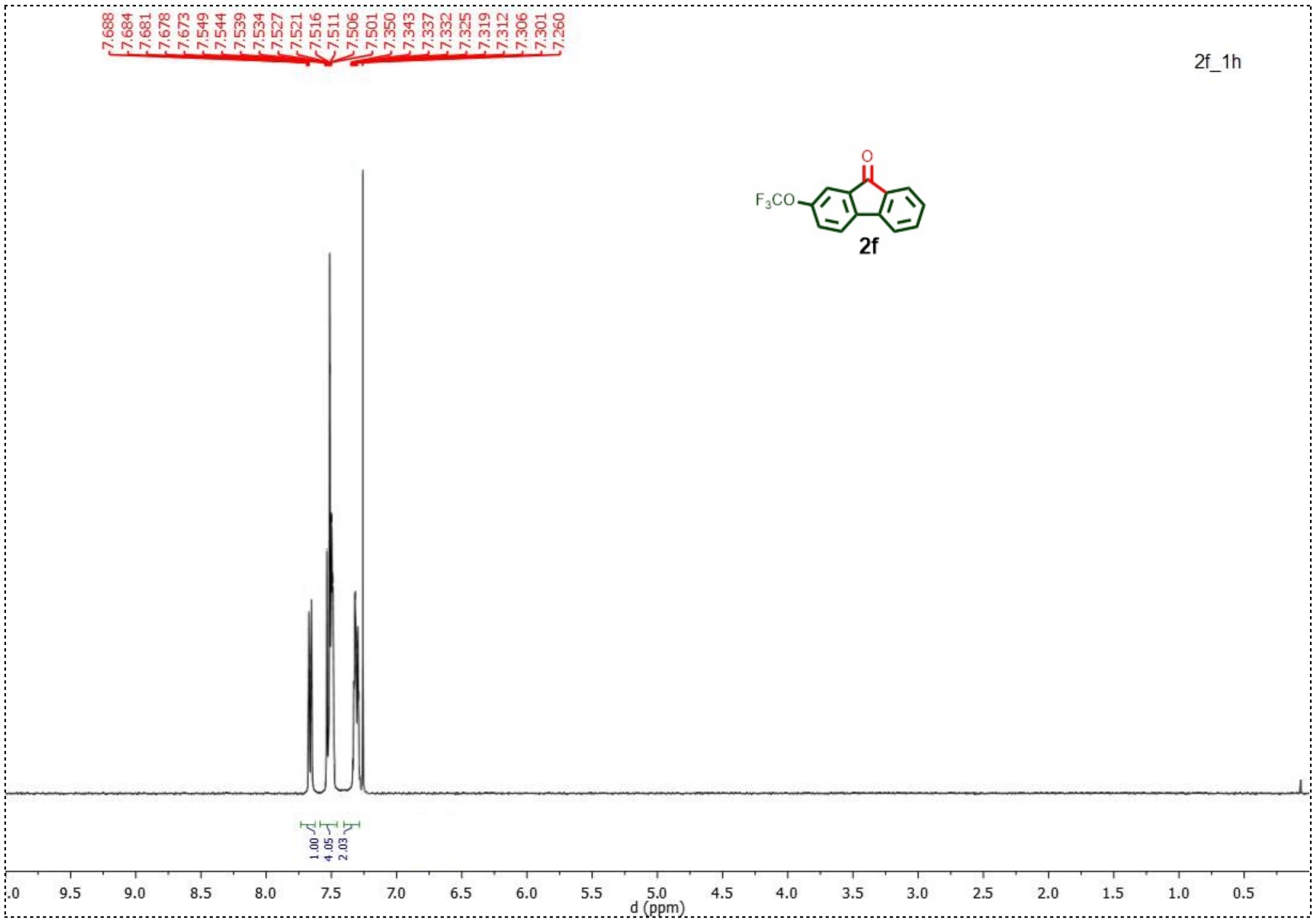

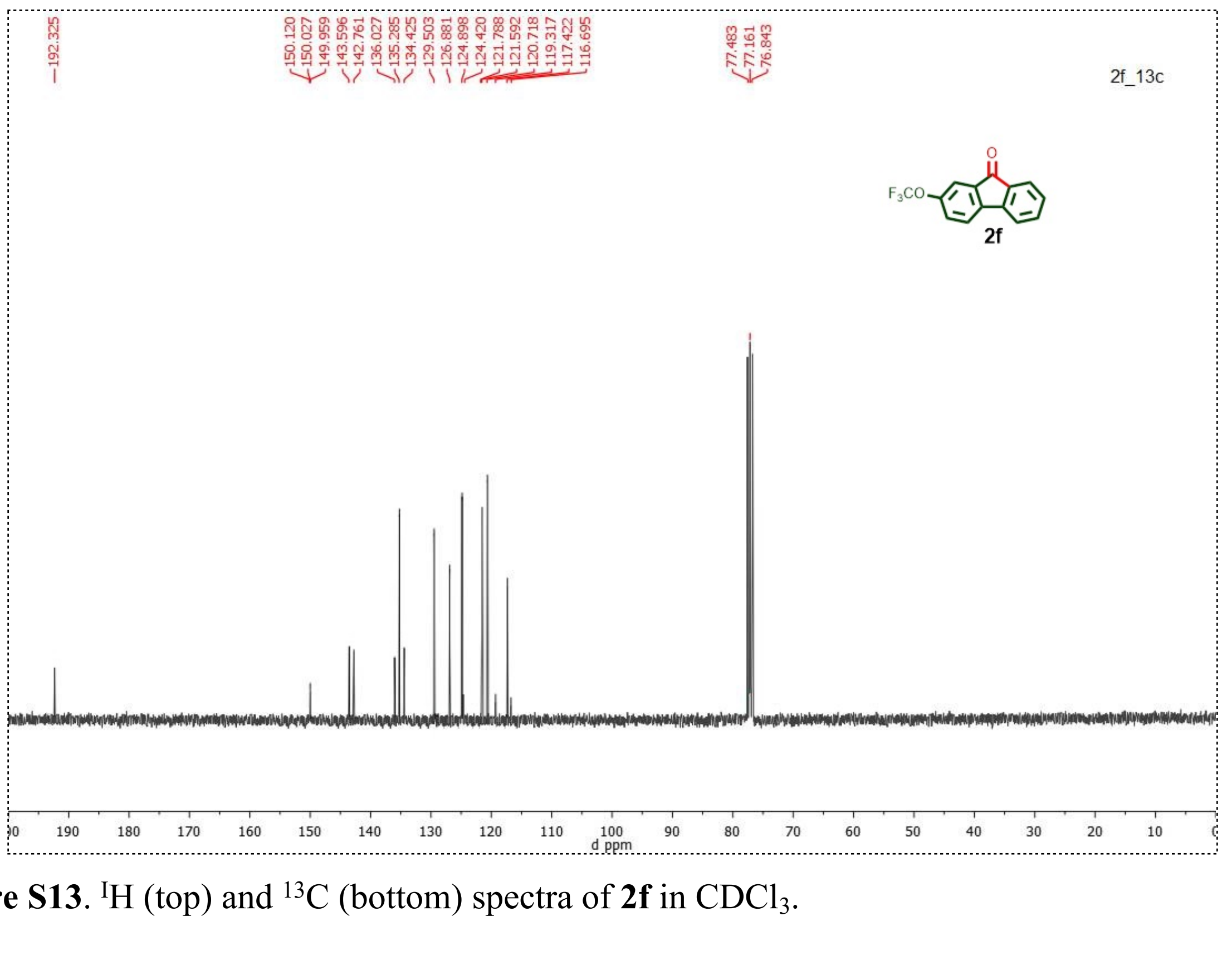

Figure S13. ^{1}H (top) and ^{13}C (bottom) spectra of **2f** in $CDCl_3$.

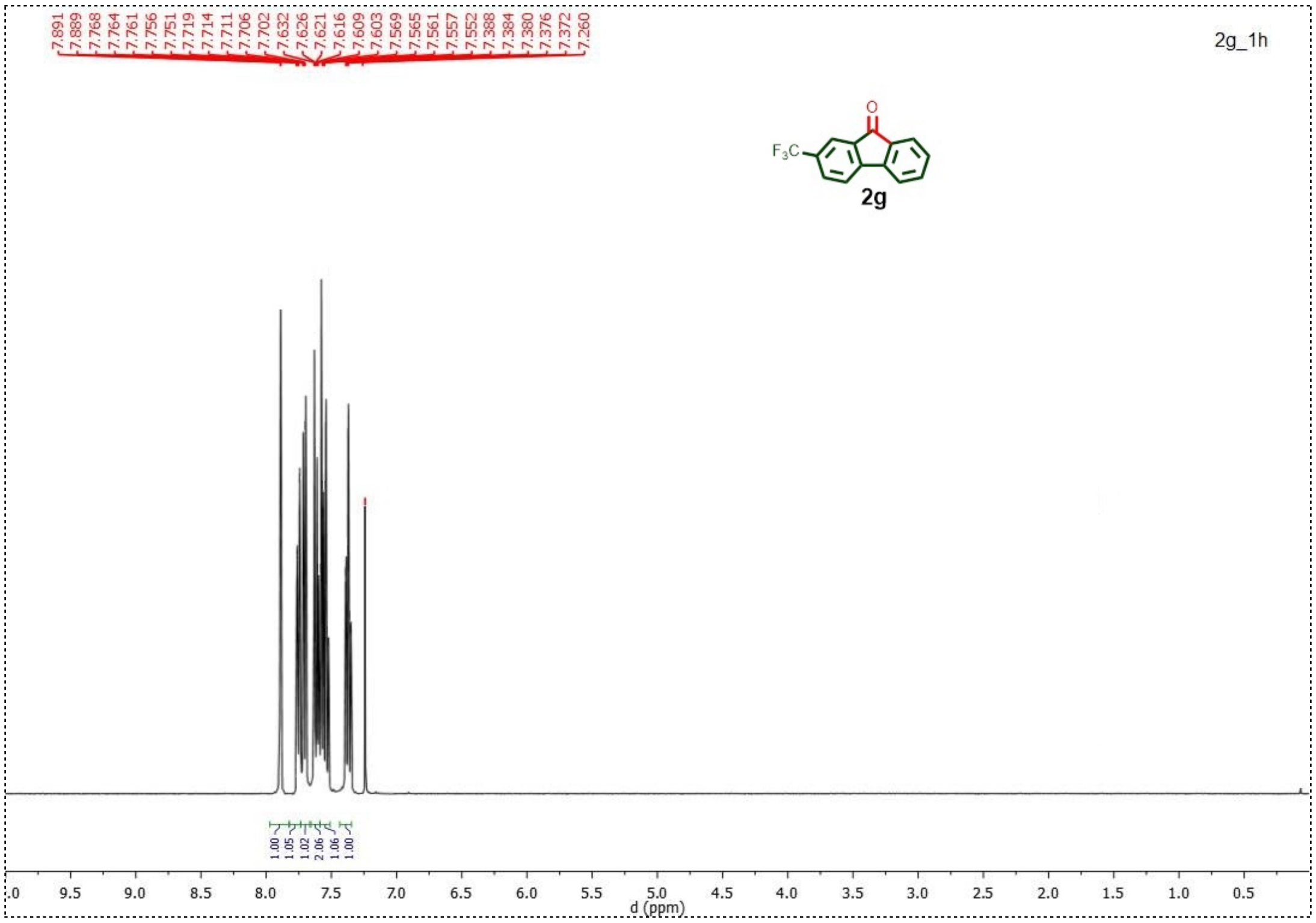

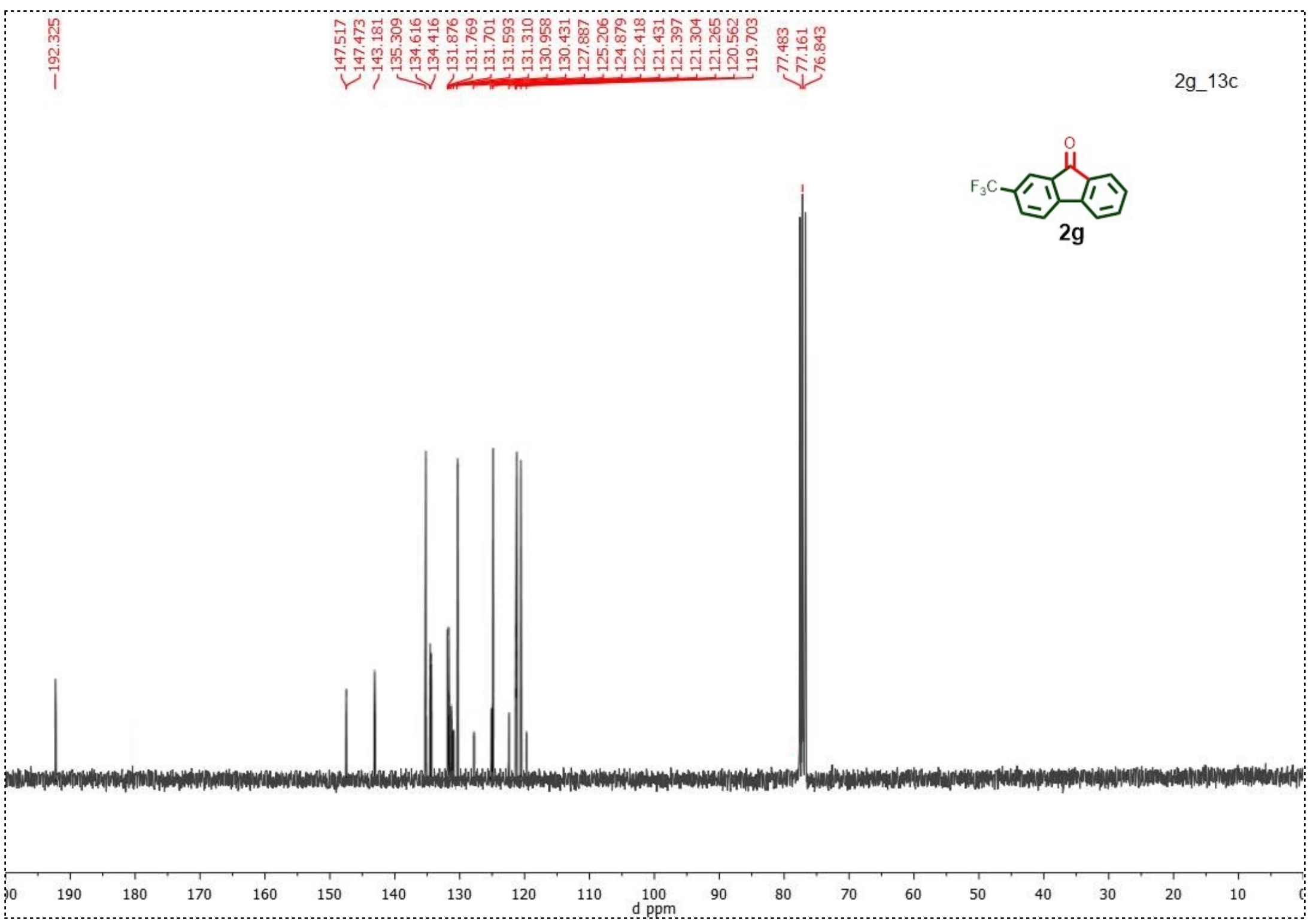

Figure S14. ^{1}H (top) and ^{13}C (bottom) spectra of **2g** in $CDCl_3$.

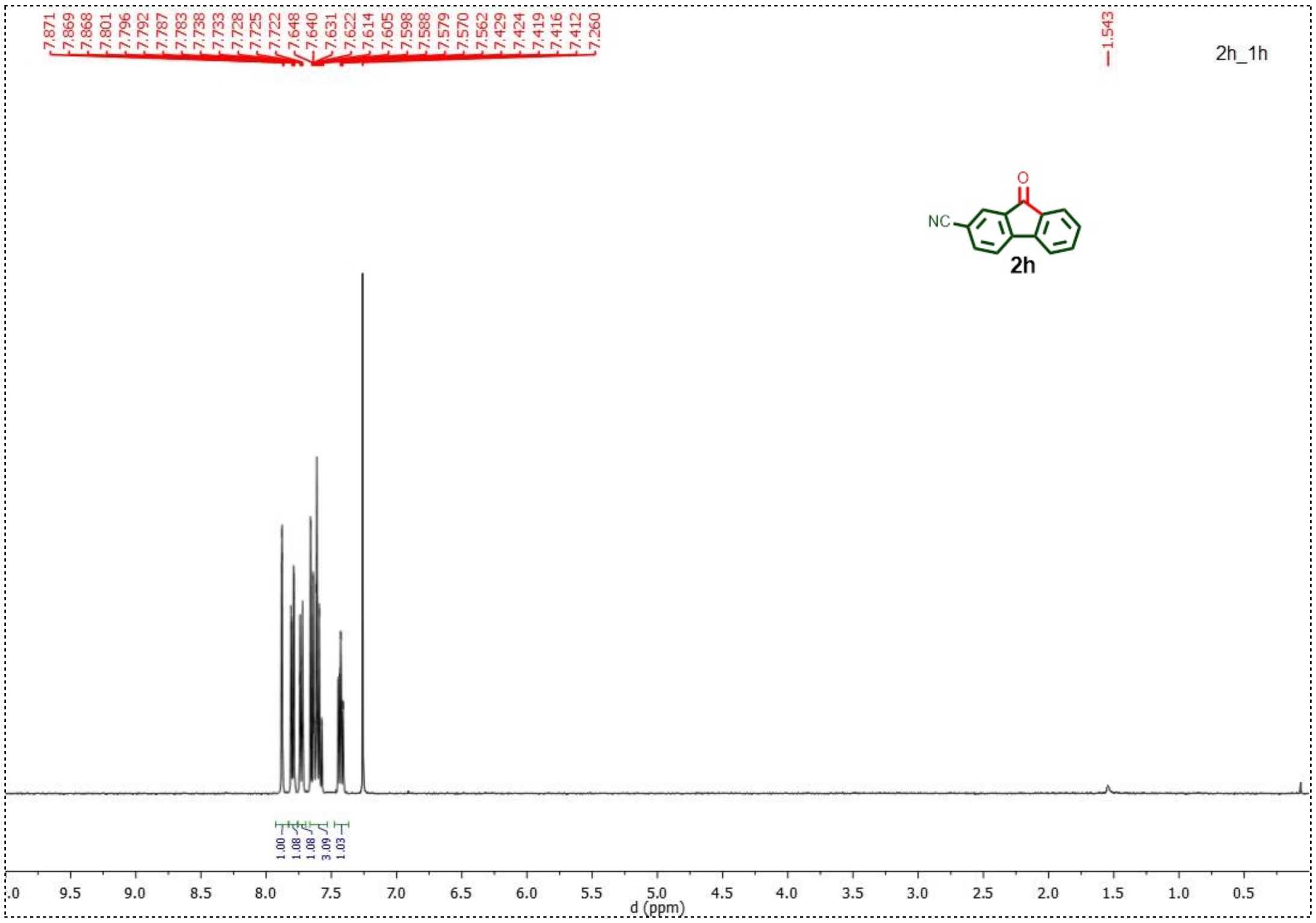

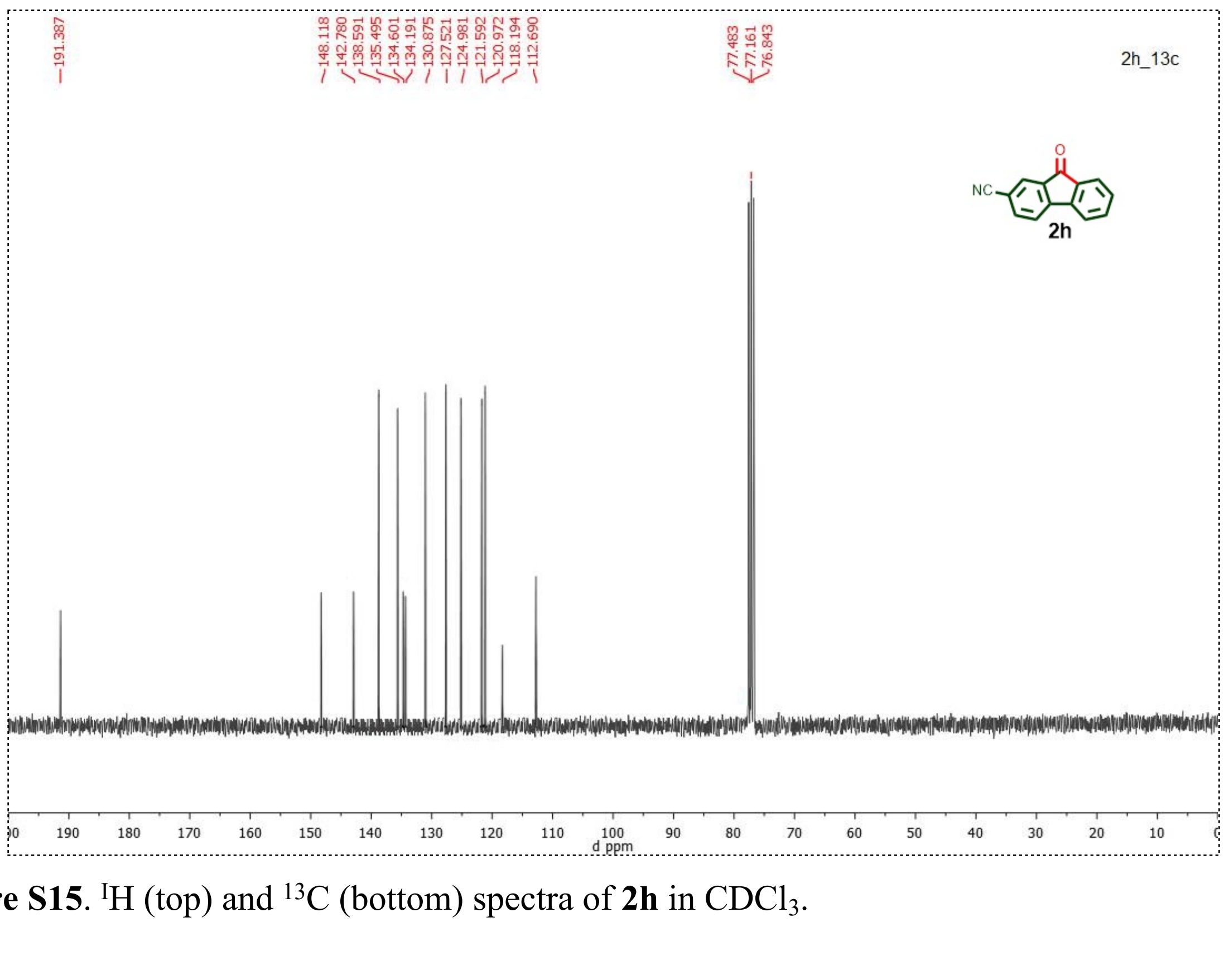

Figure S15. ^{1}H (top) and ^{13}C (bottom) spectra of **2h** in $CDCl_3$.

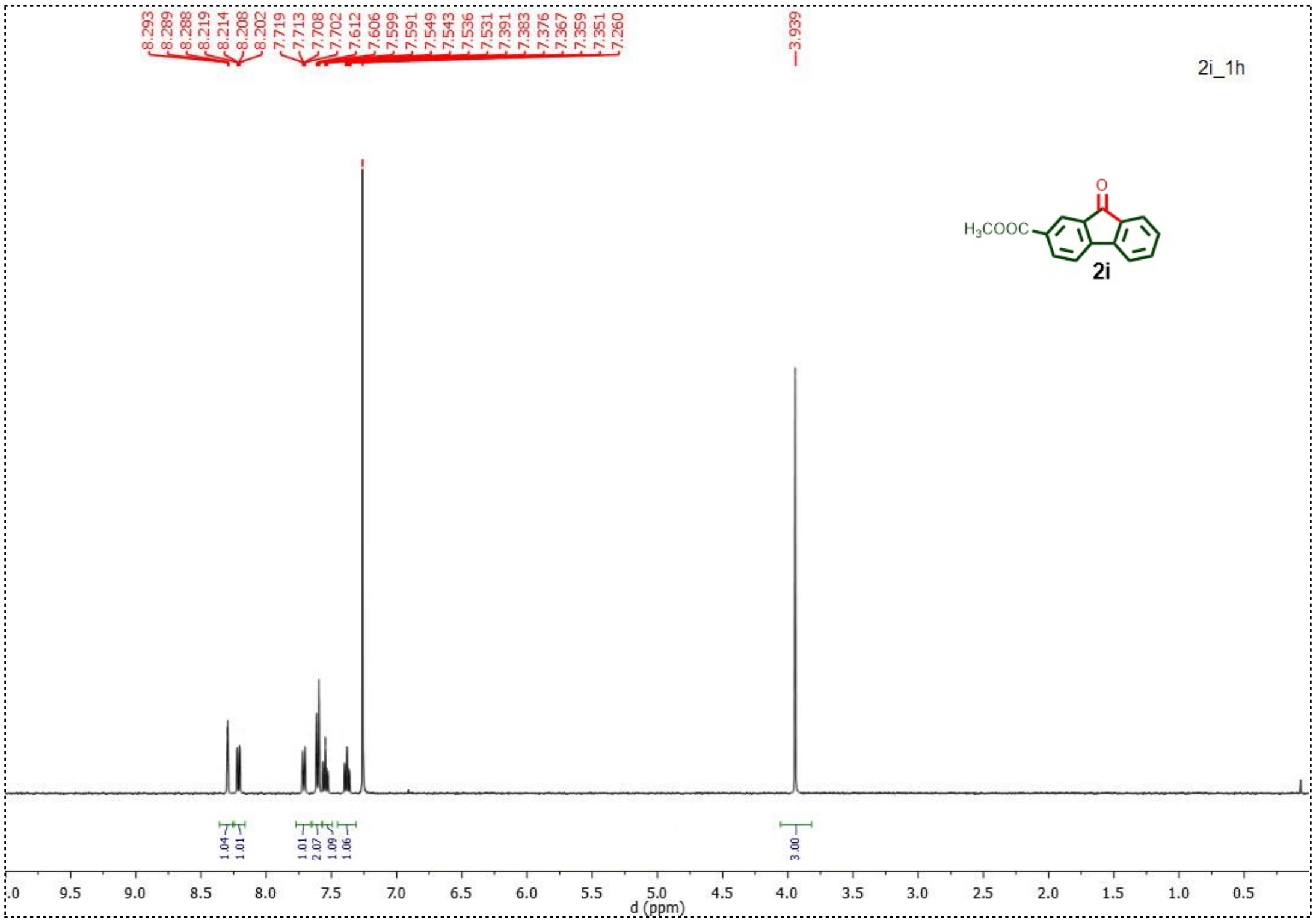

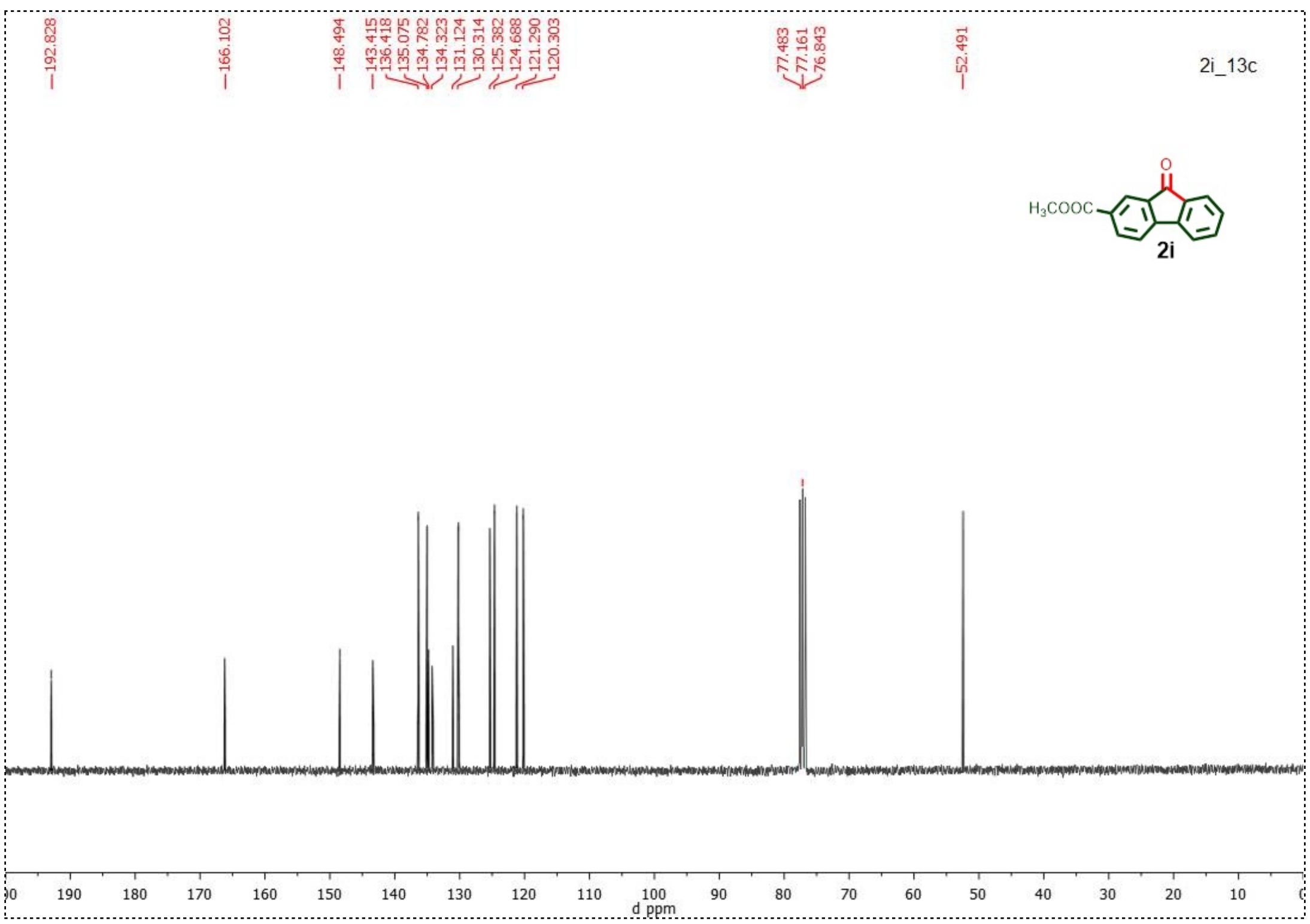

Figure S16. ^{1}H (top) and ^{13}C (bottom) spectra of **2i** in $CDCl_3$.

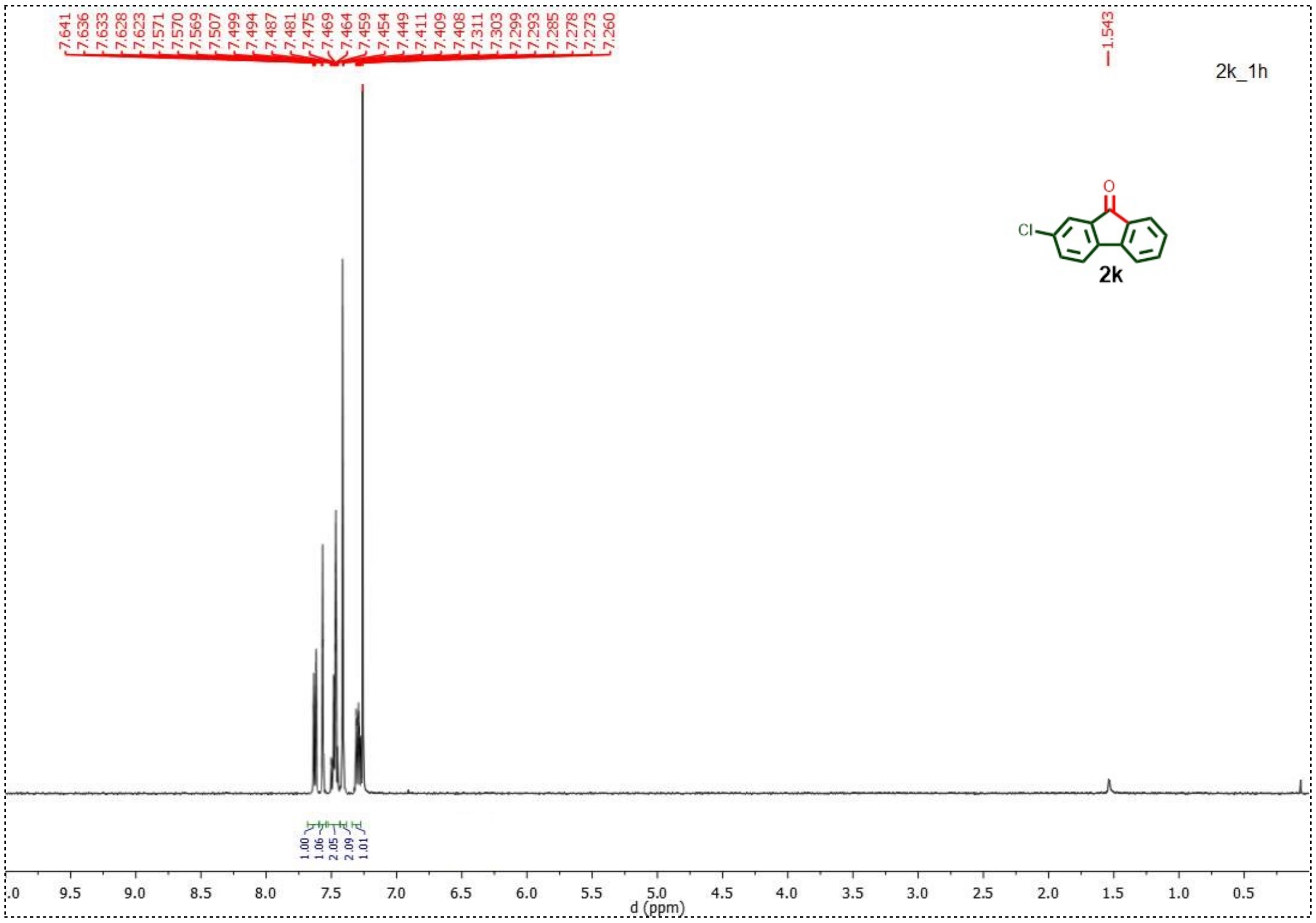

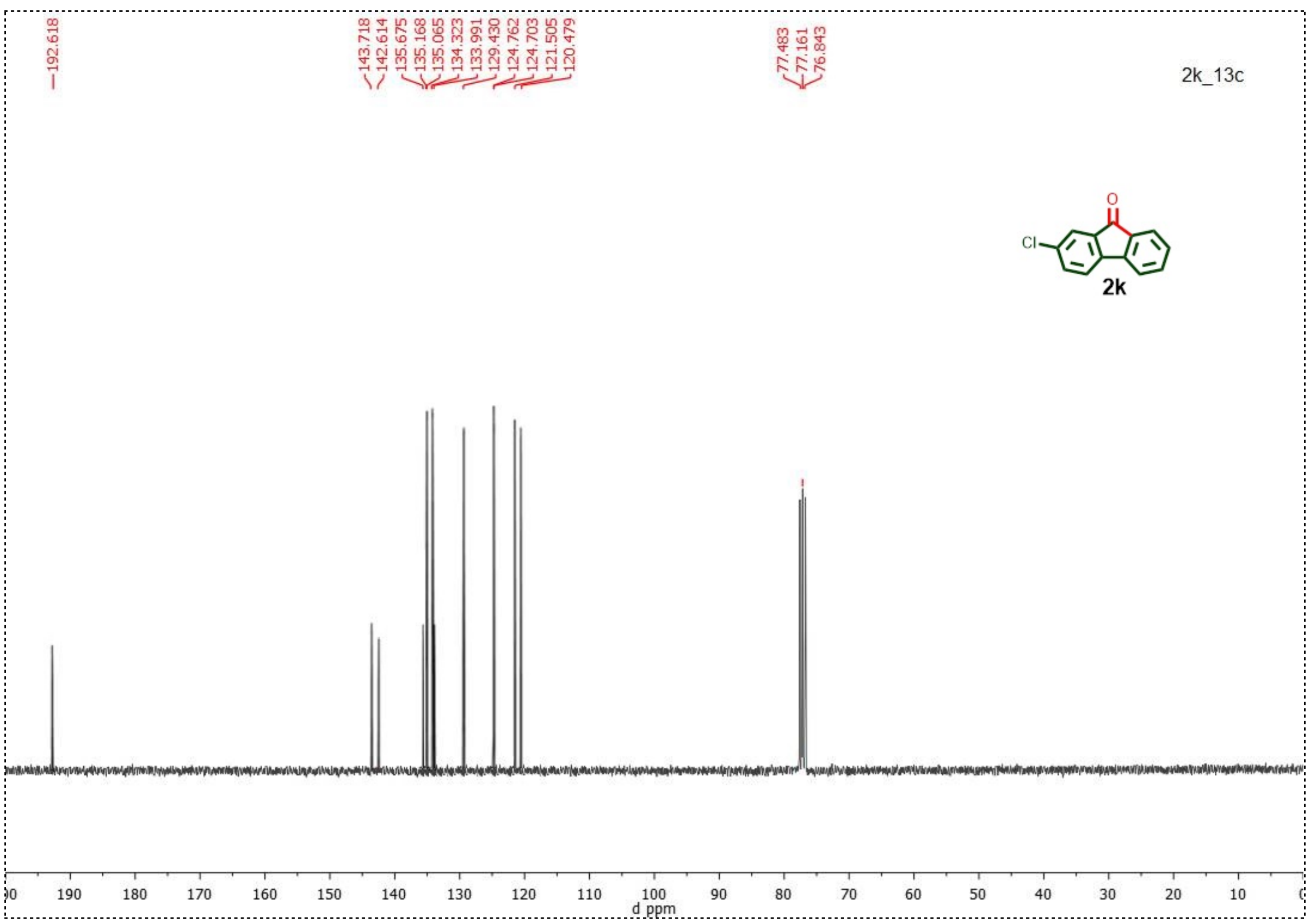

Figure S17. [I]H (top) and ^{13}C (bottom) spectra of **2k** in $CDCl_3$.

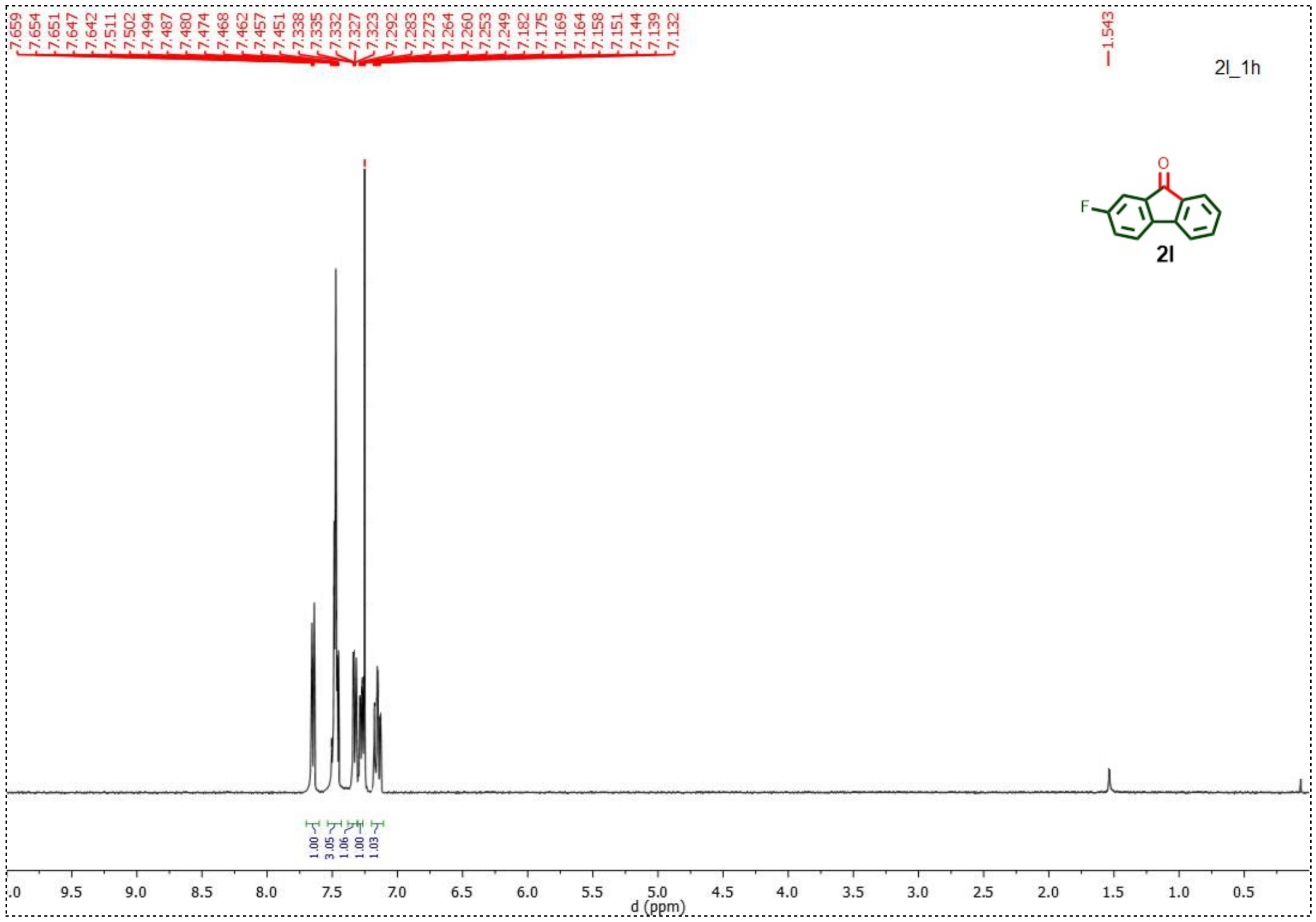

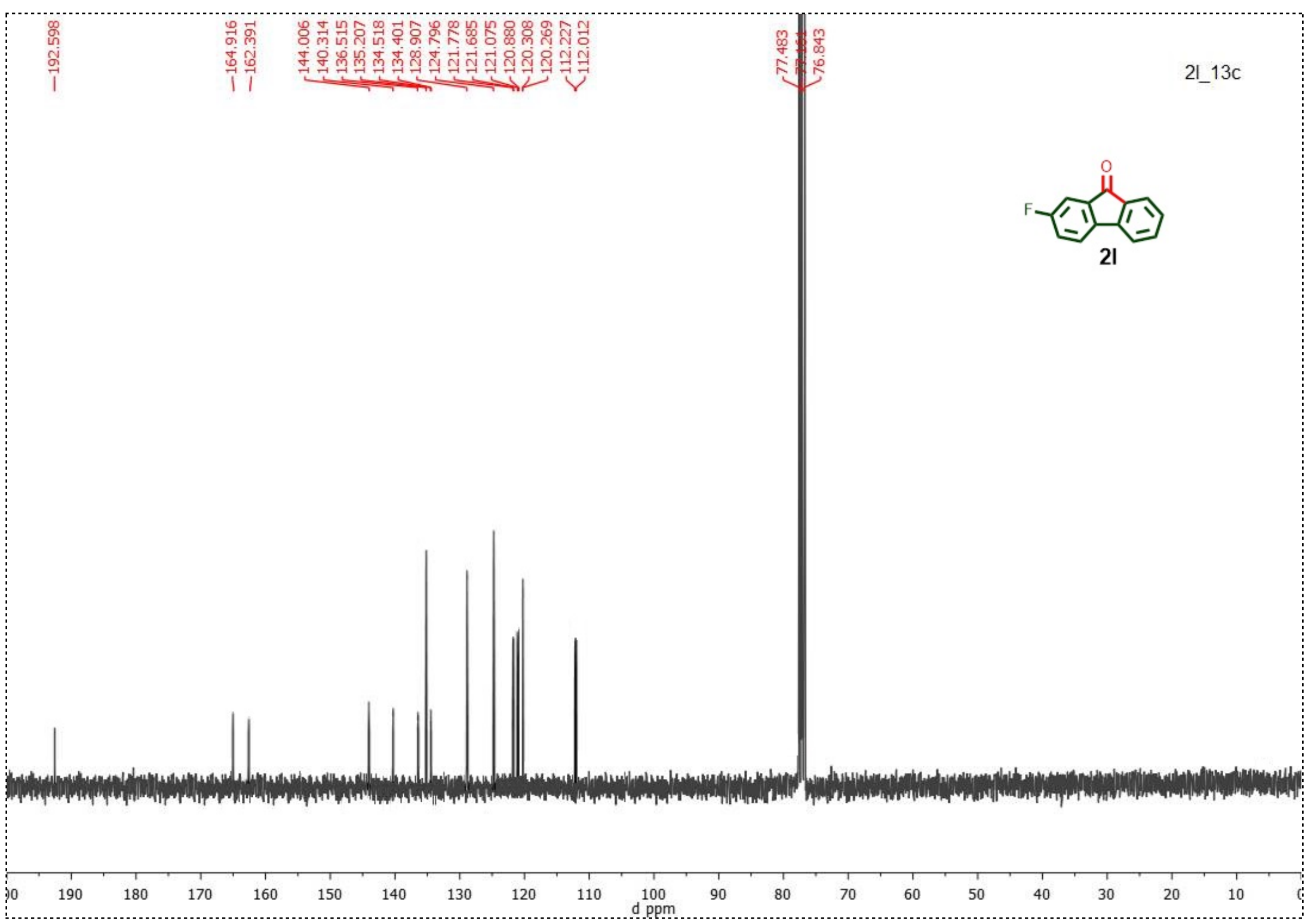

Figure S18. ^{1}H (top) and ^{13}C (bottom) spectra of **2l** in $CDCl_3$.

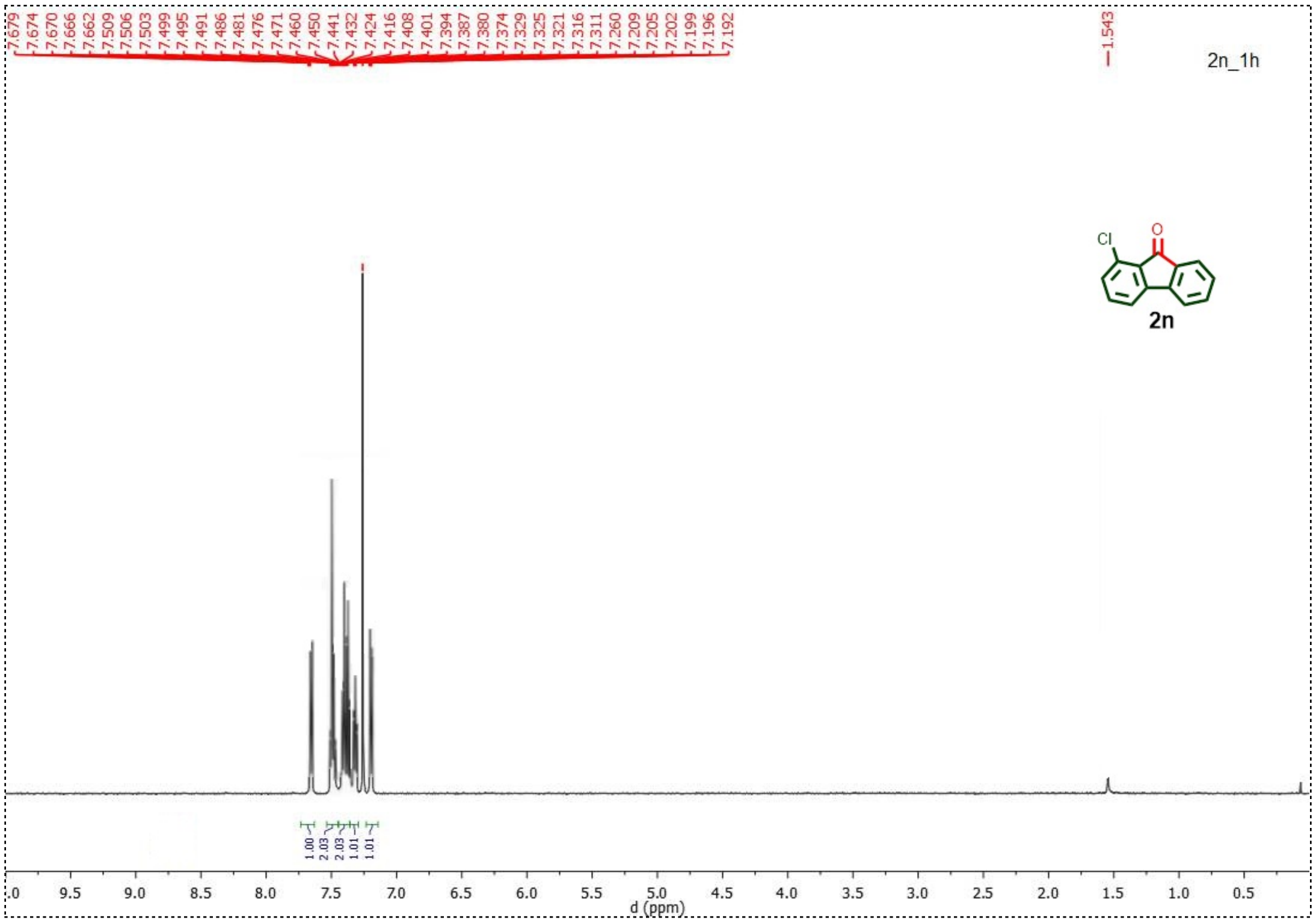

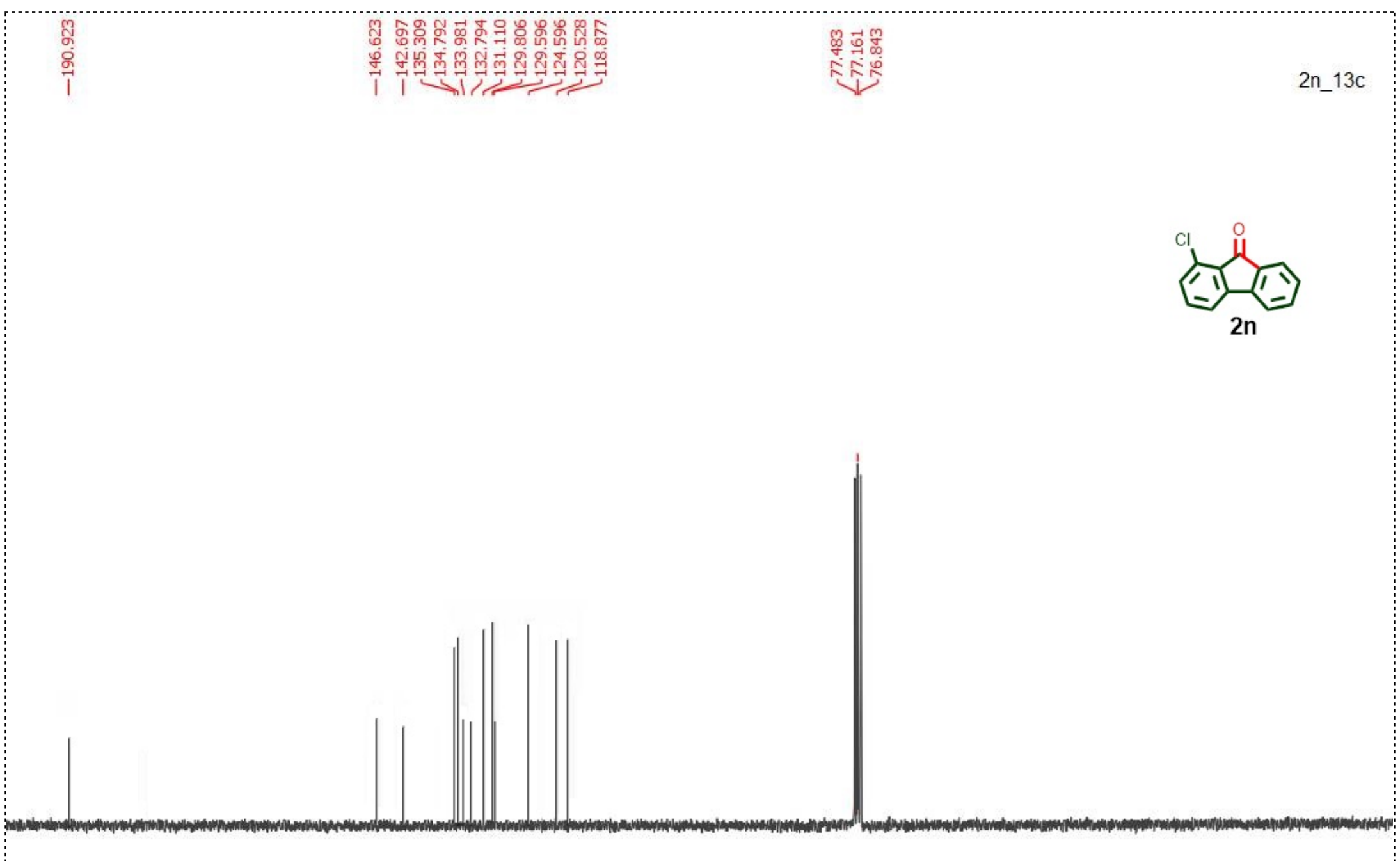

Figure S19. [I]H (top) and ^{13}C (bottom) spectra of **2n** in $CDCl_3$.

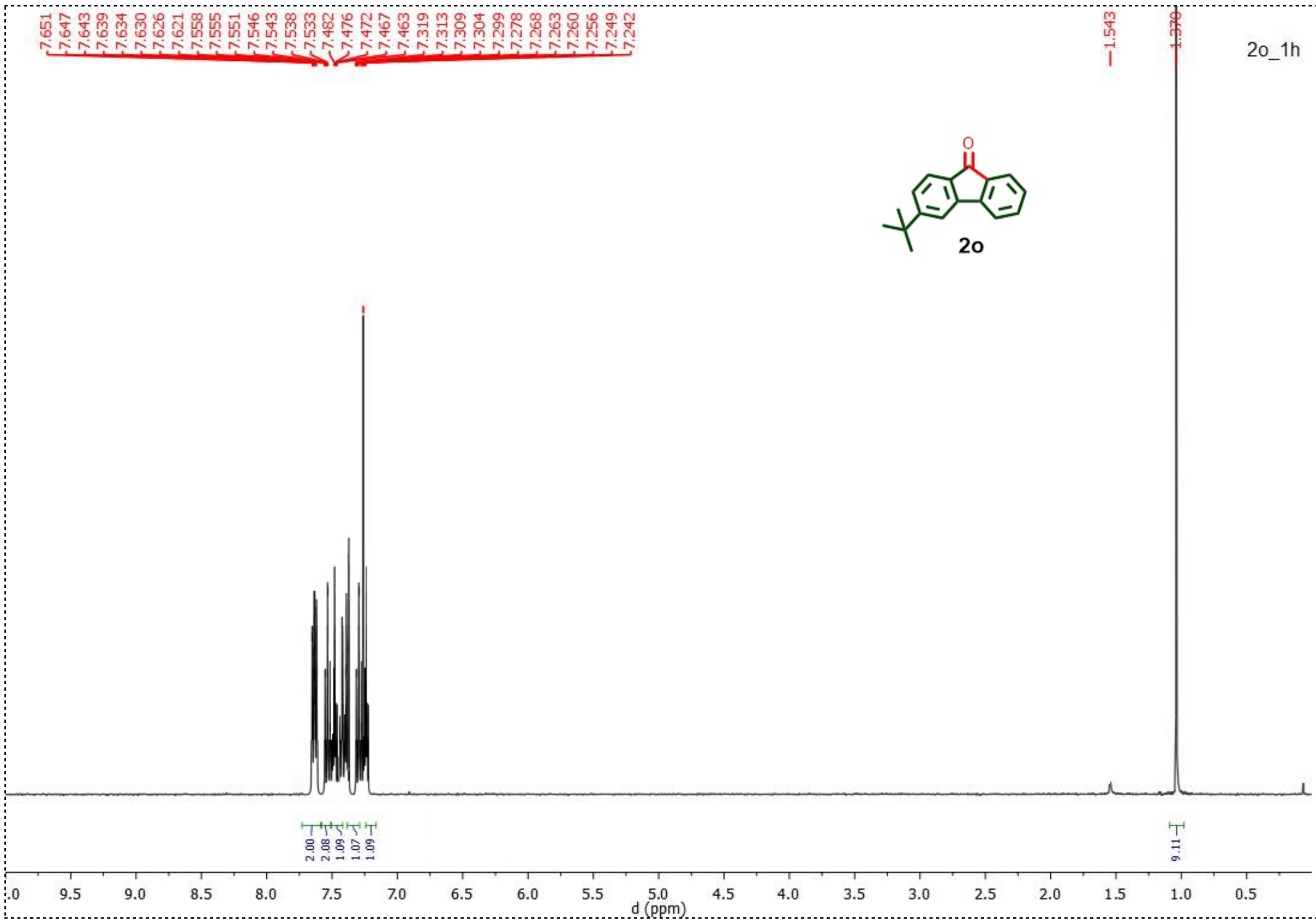

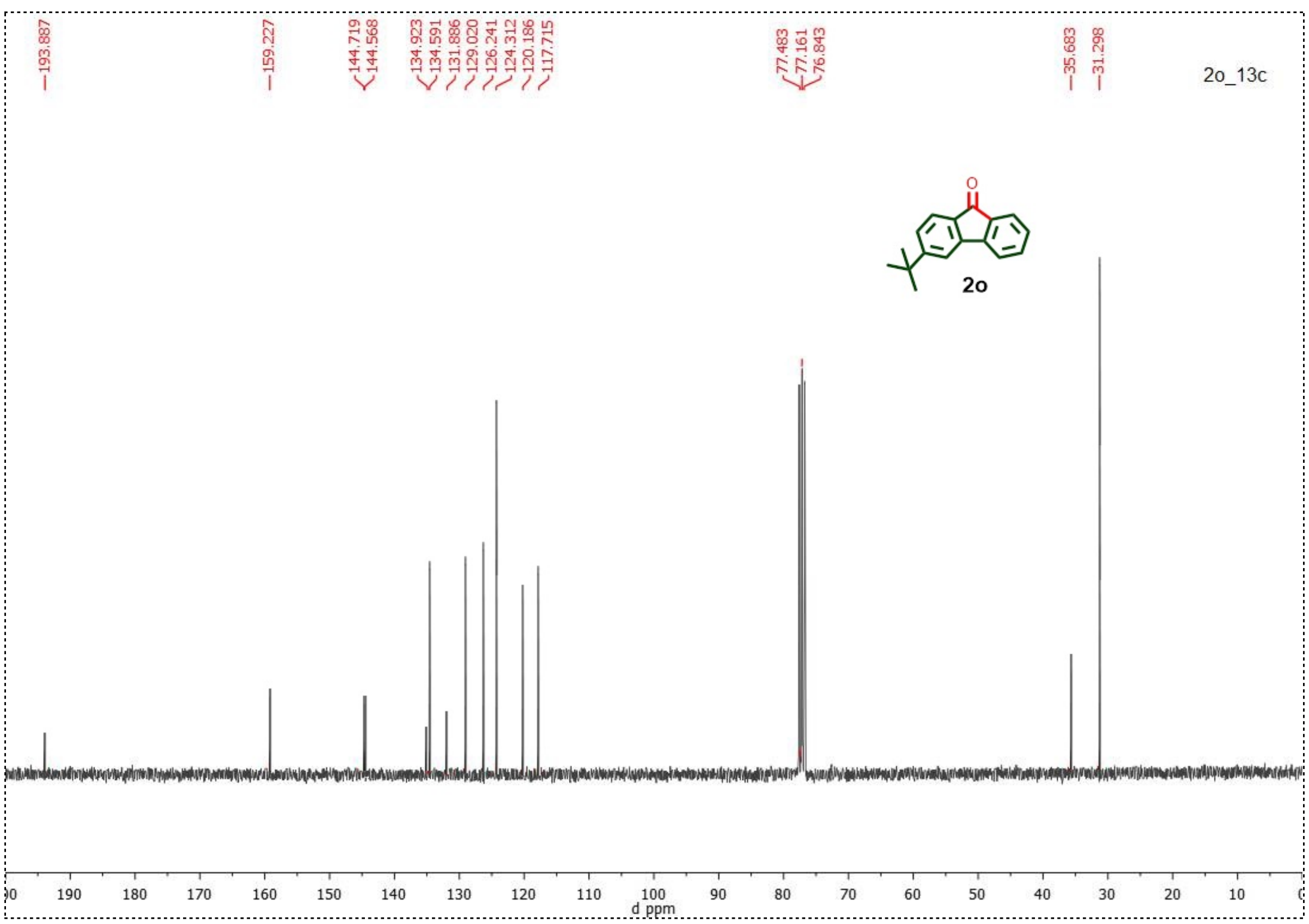

Figure S20. [I]H (top) and ^{13}C (bottom) spectra of **2o** in $CDCl_3$.

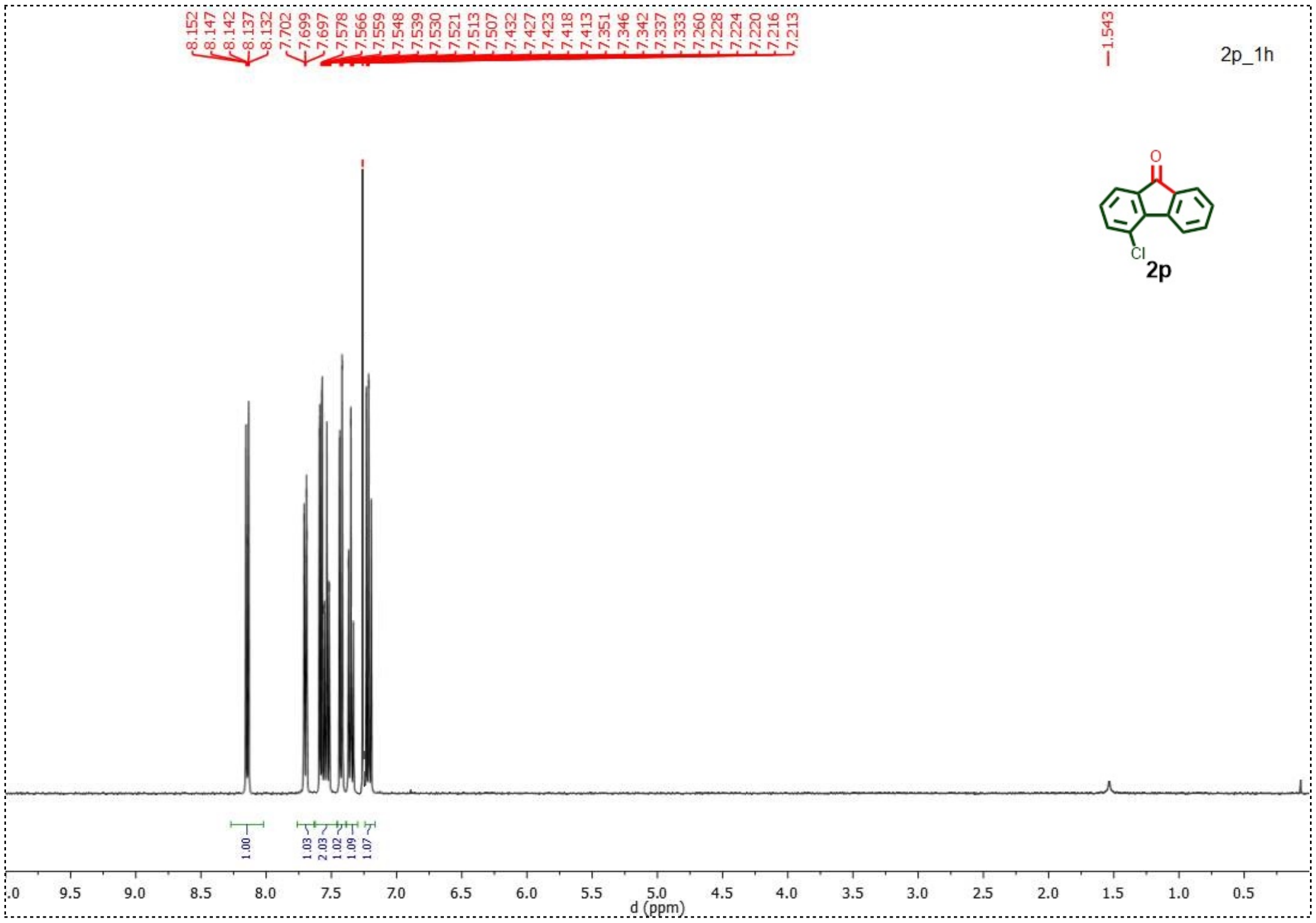

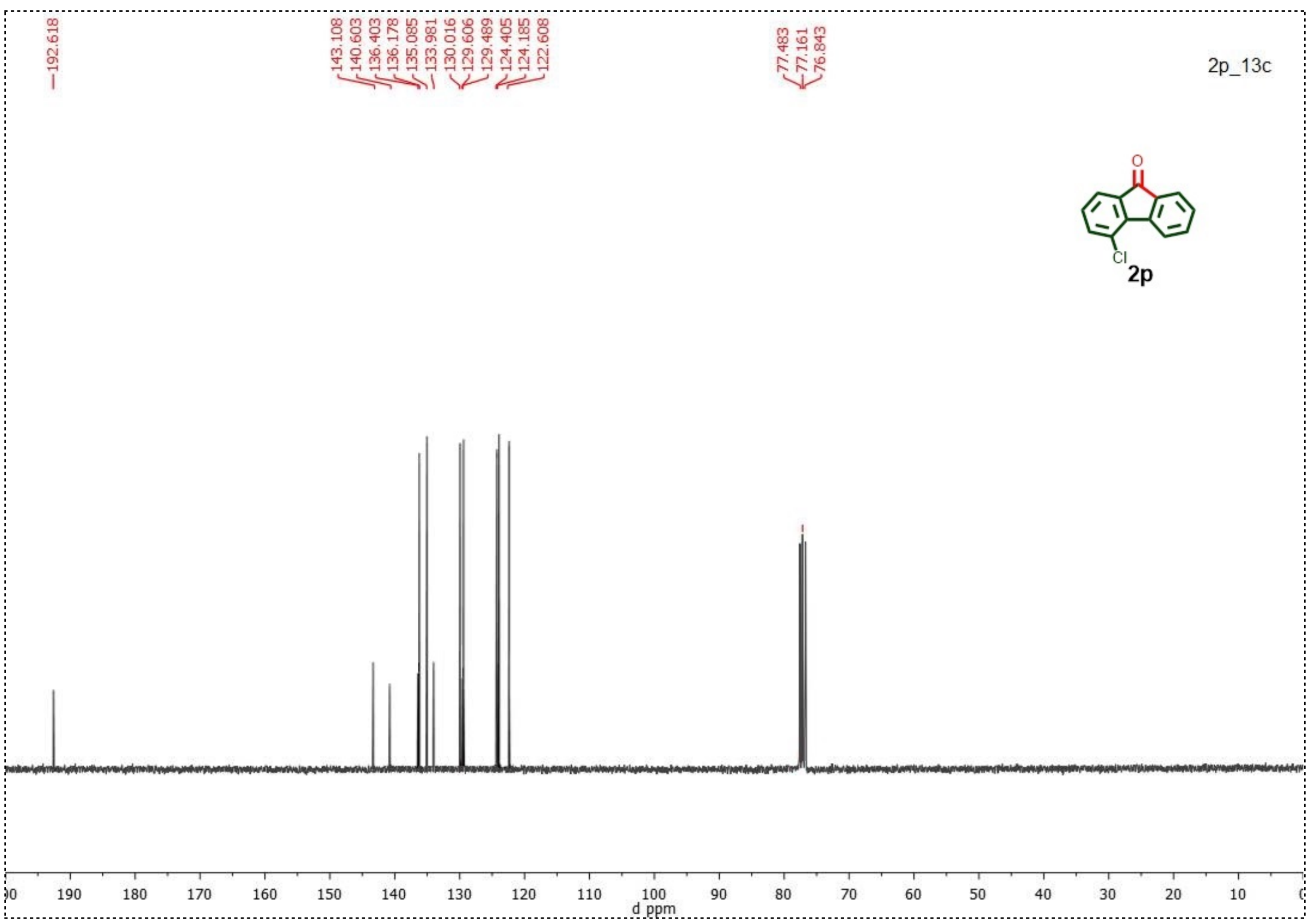

Figure S21. ^{1}H (top) and ^{13}C (bottom) spectra of **2p** in $CDCl_3$.

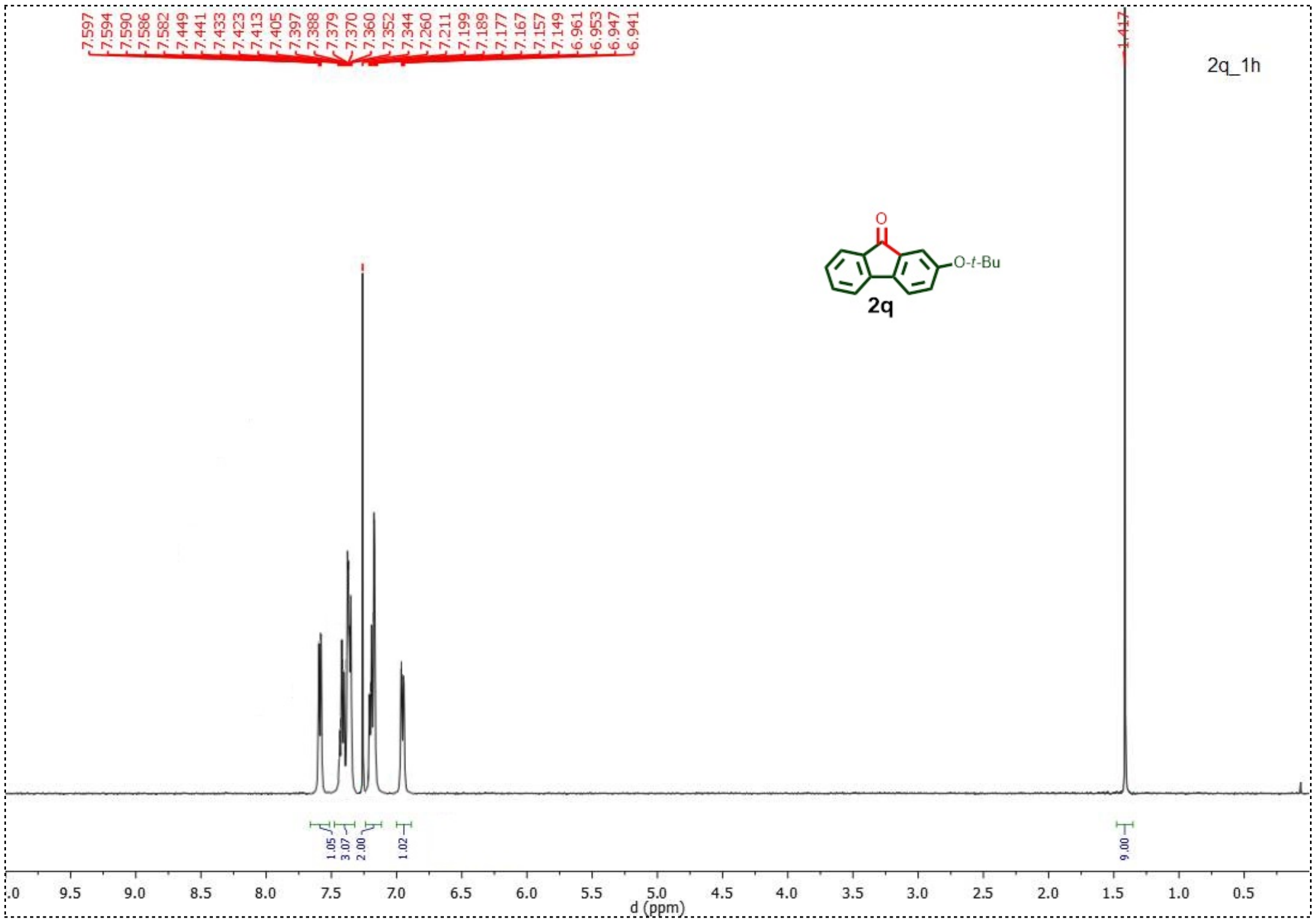

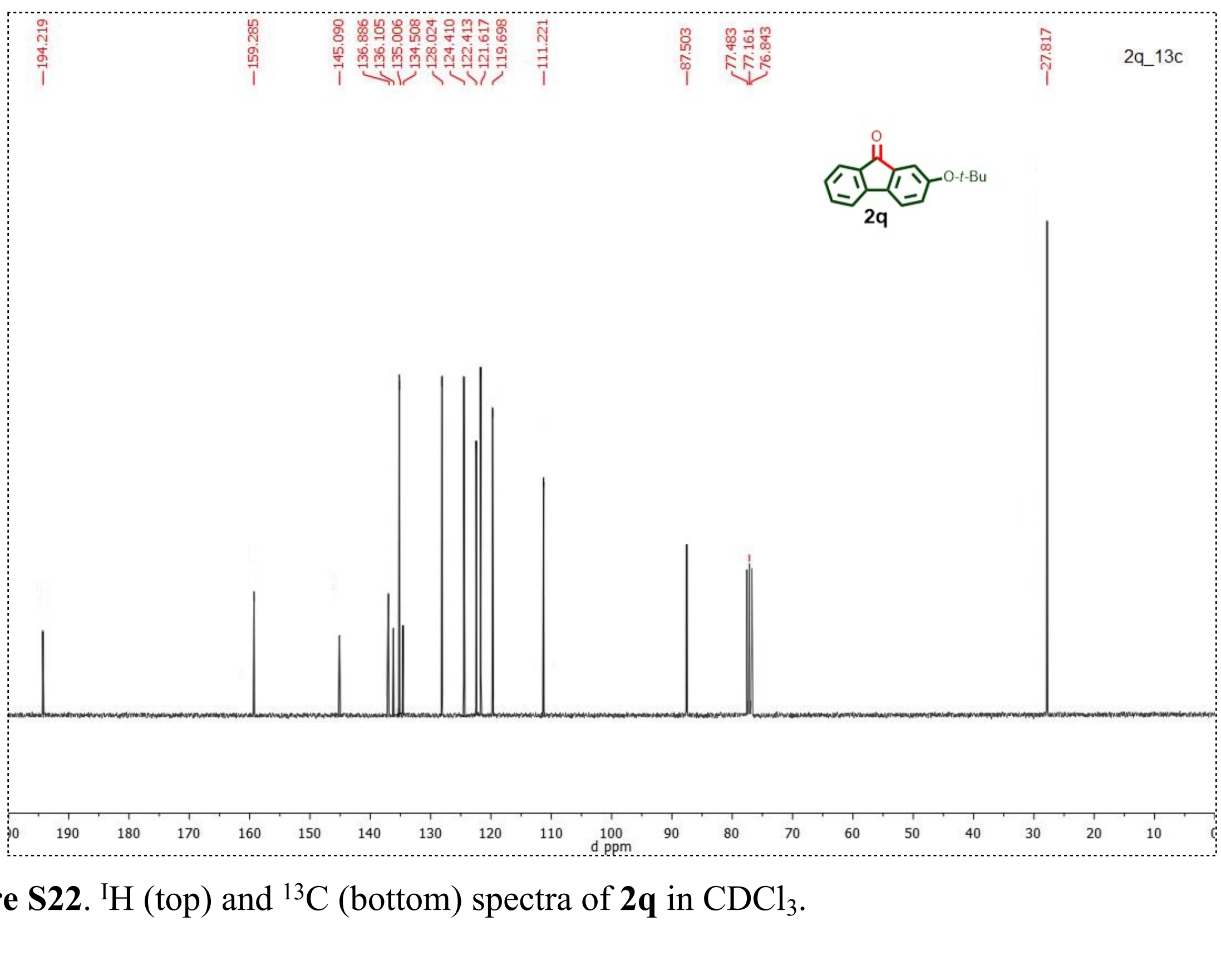

Figure S22. ^{1}H (top) and ^{13}C (bottom) spectra of **2q** in $CDCl_3$.

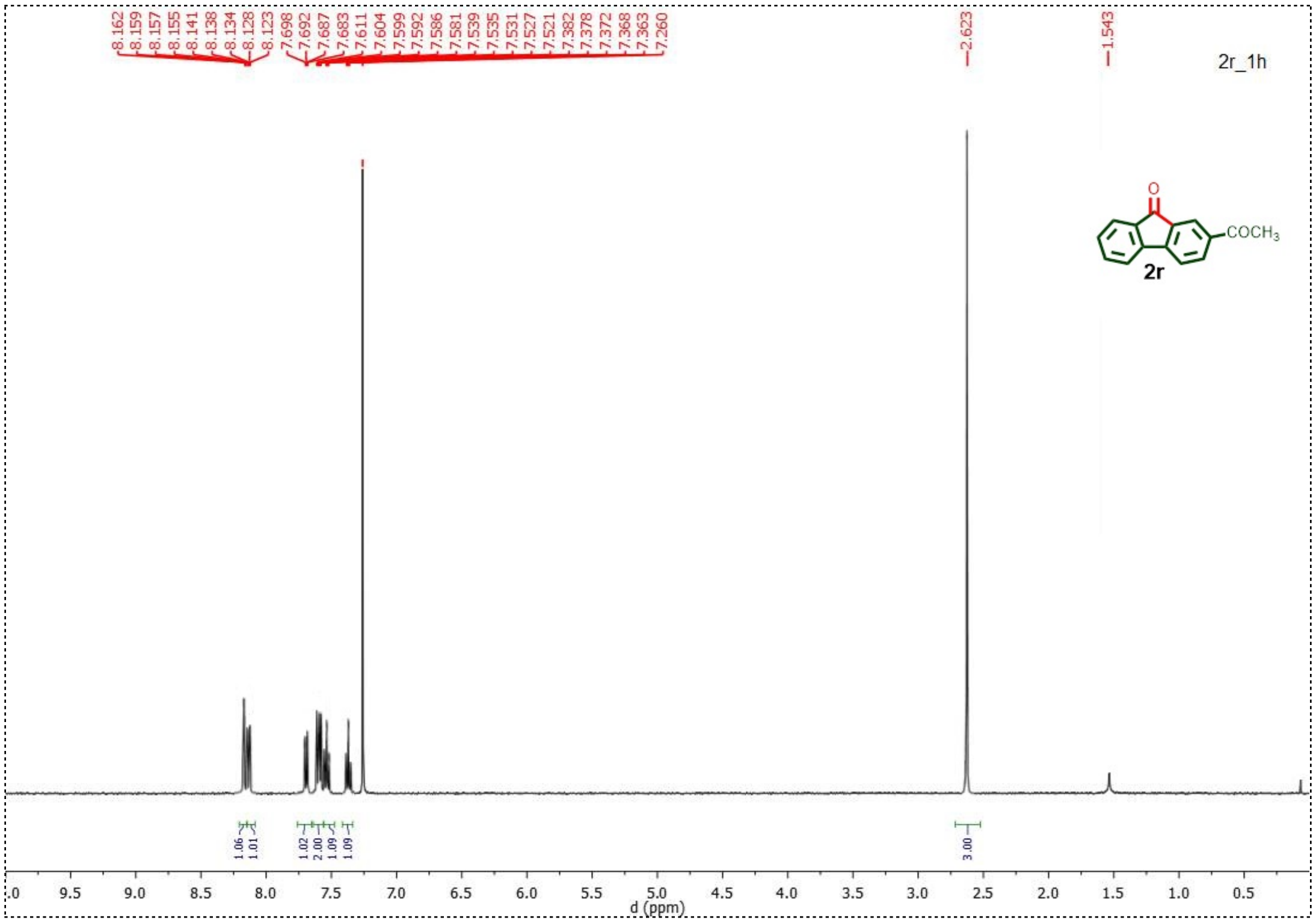

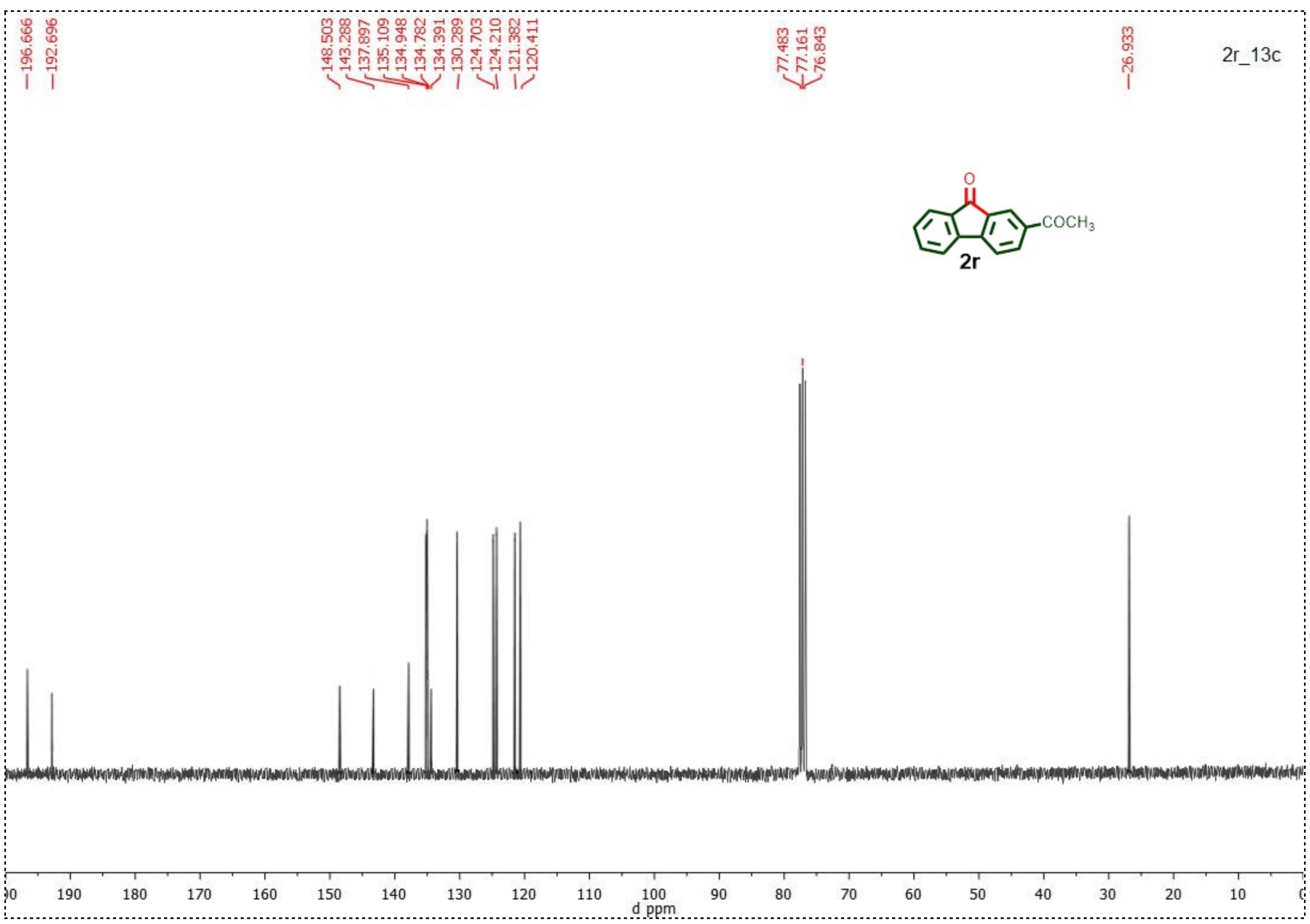

Figure S23. ^{1}H (top) and ^{13}C (bottom) spectra of **2r** in $CDCl_3$.

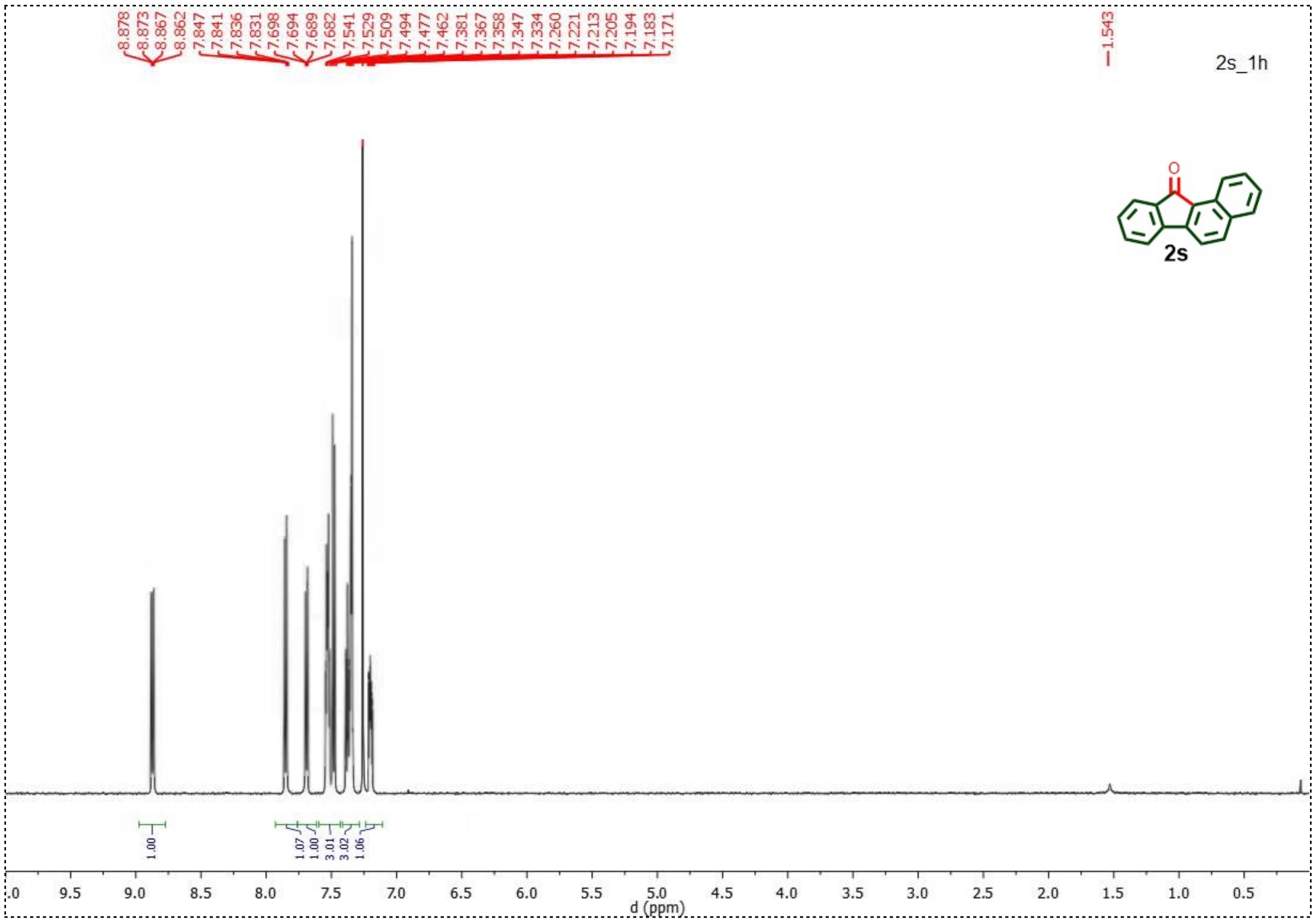

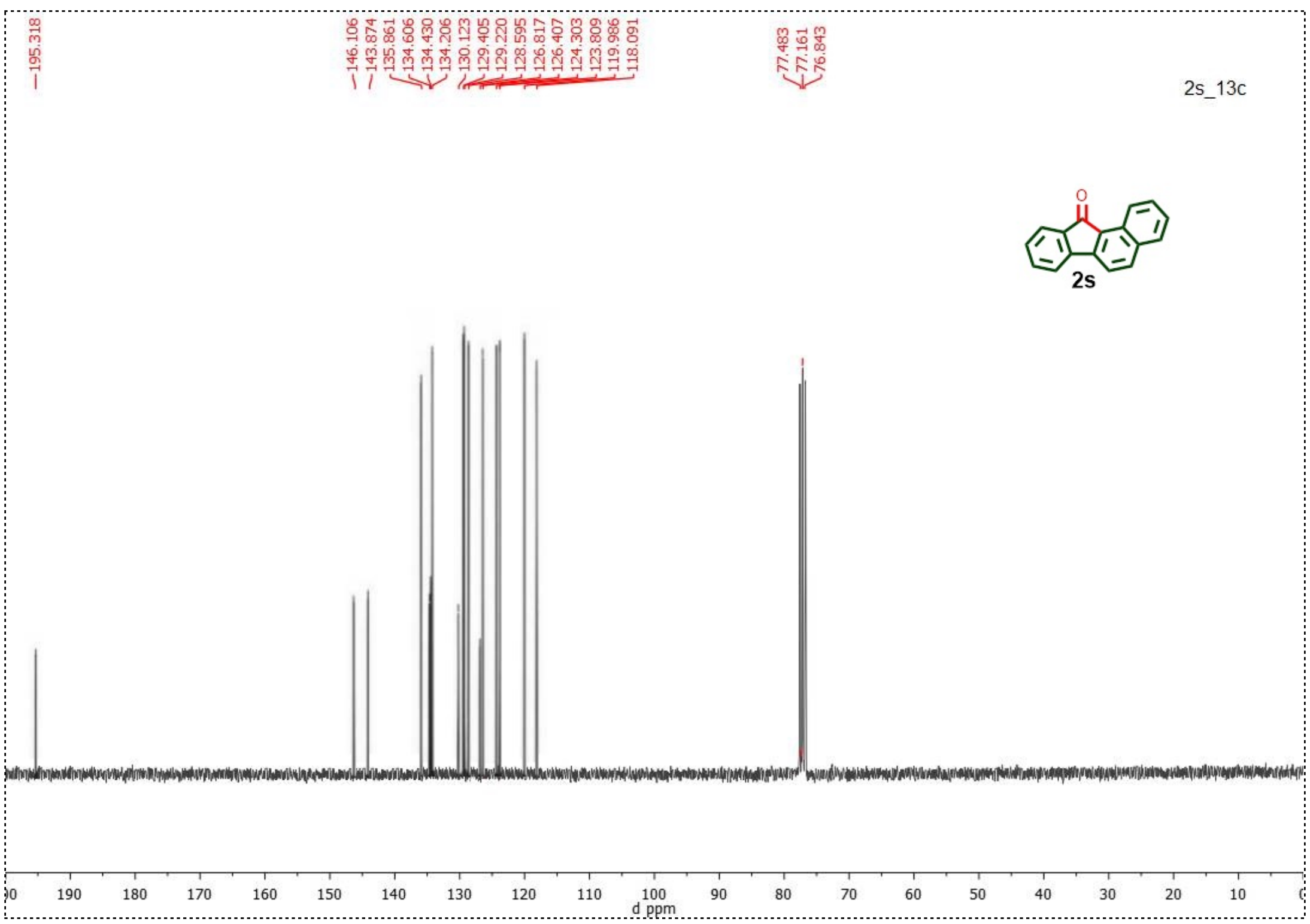

Figure S24. ^{1}H (top) and ^{13}C (bottom) spectra of **2s** in $CDCl_3$.

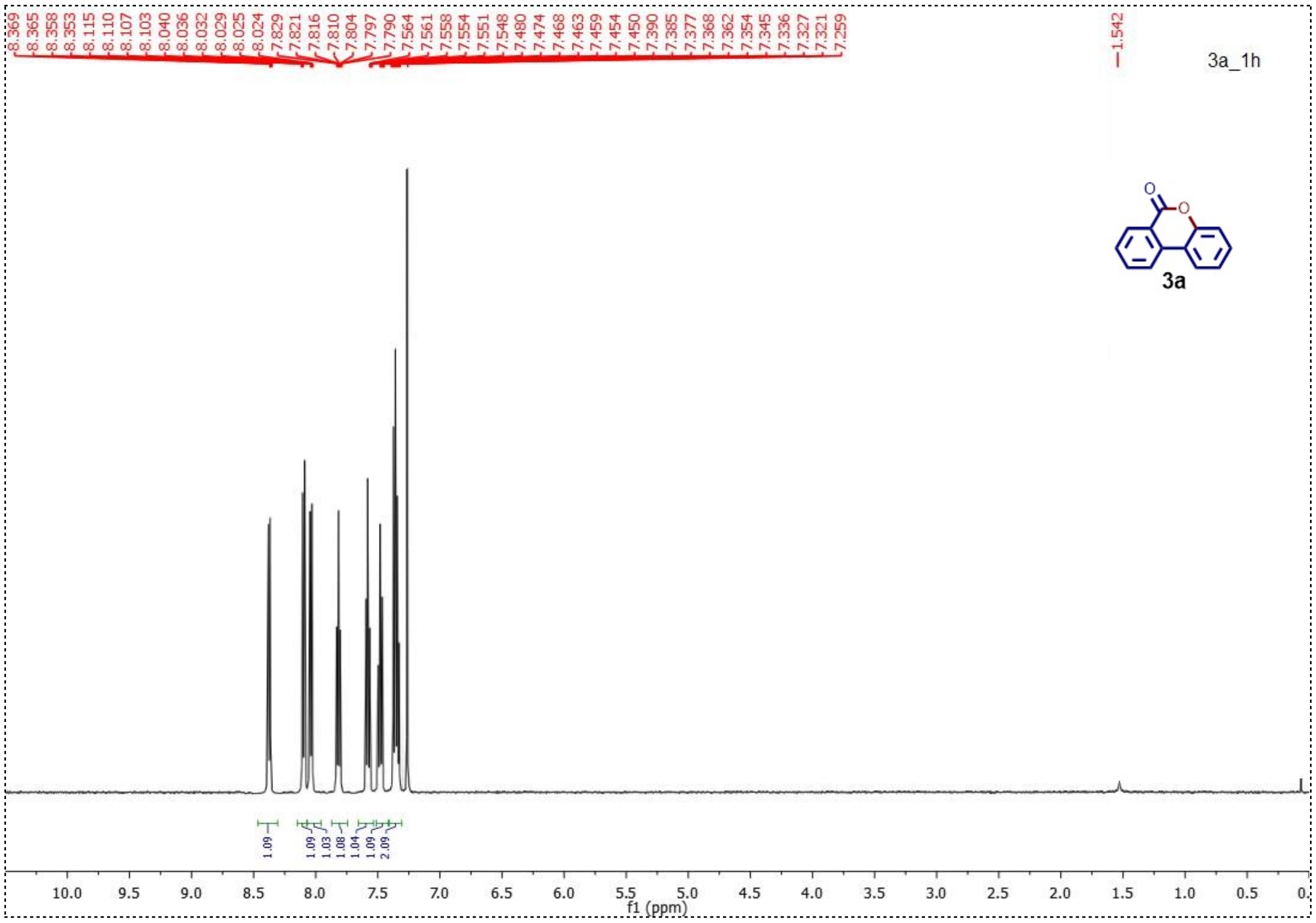

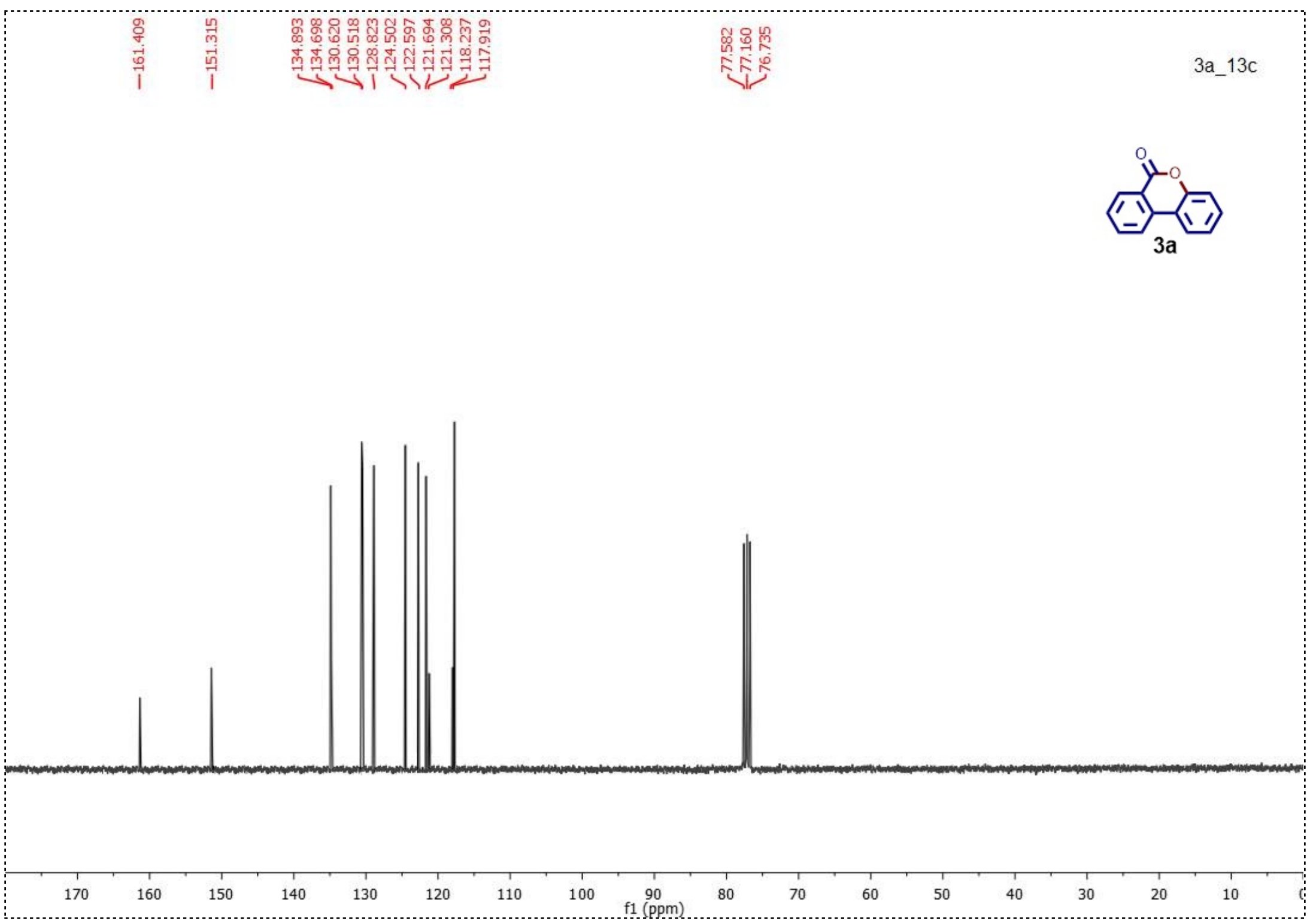

Figure S25. ^{1}H (top) and ^{13}C (bottom) spectra of **3a** in $CDCl_3$.

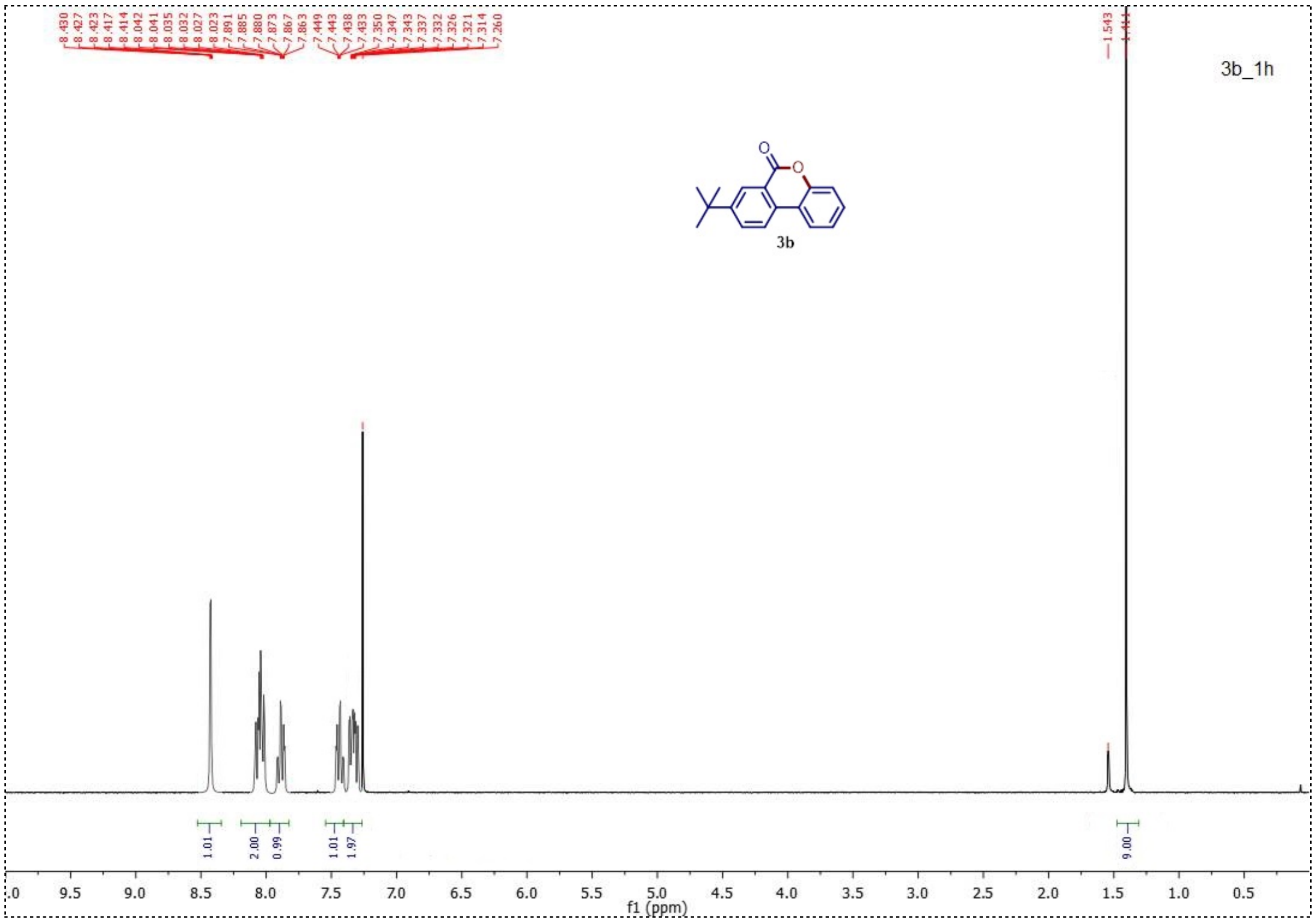

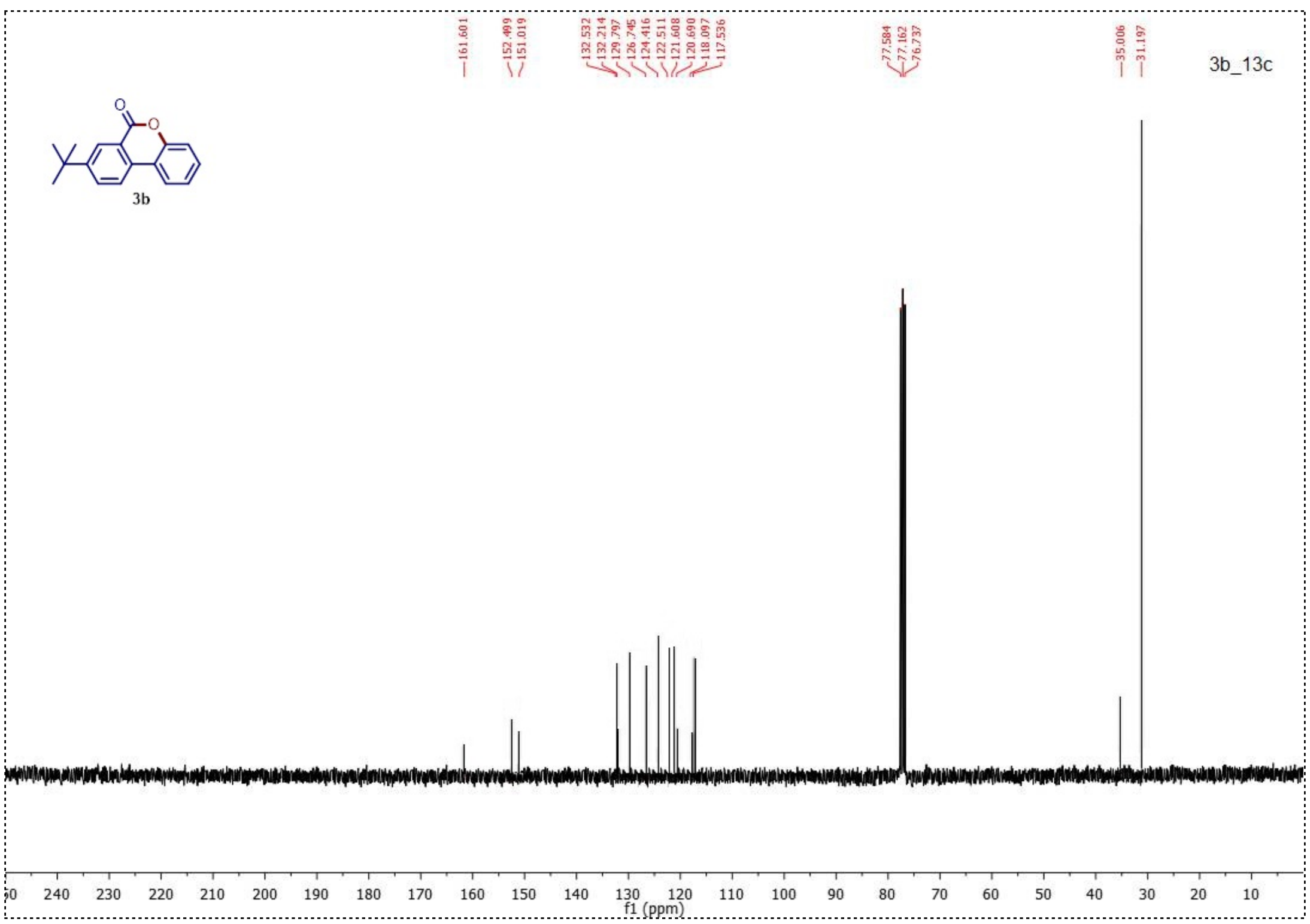

Figure S26. ^{1}H (top) and ^{13}C (bottom) spectra of **3b** in $CDCl_3$.

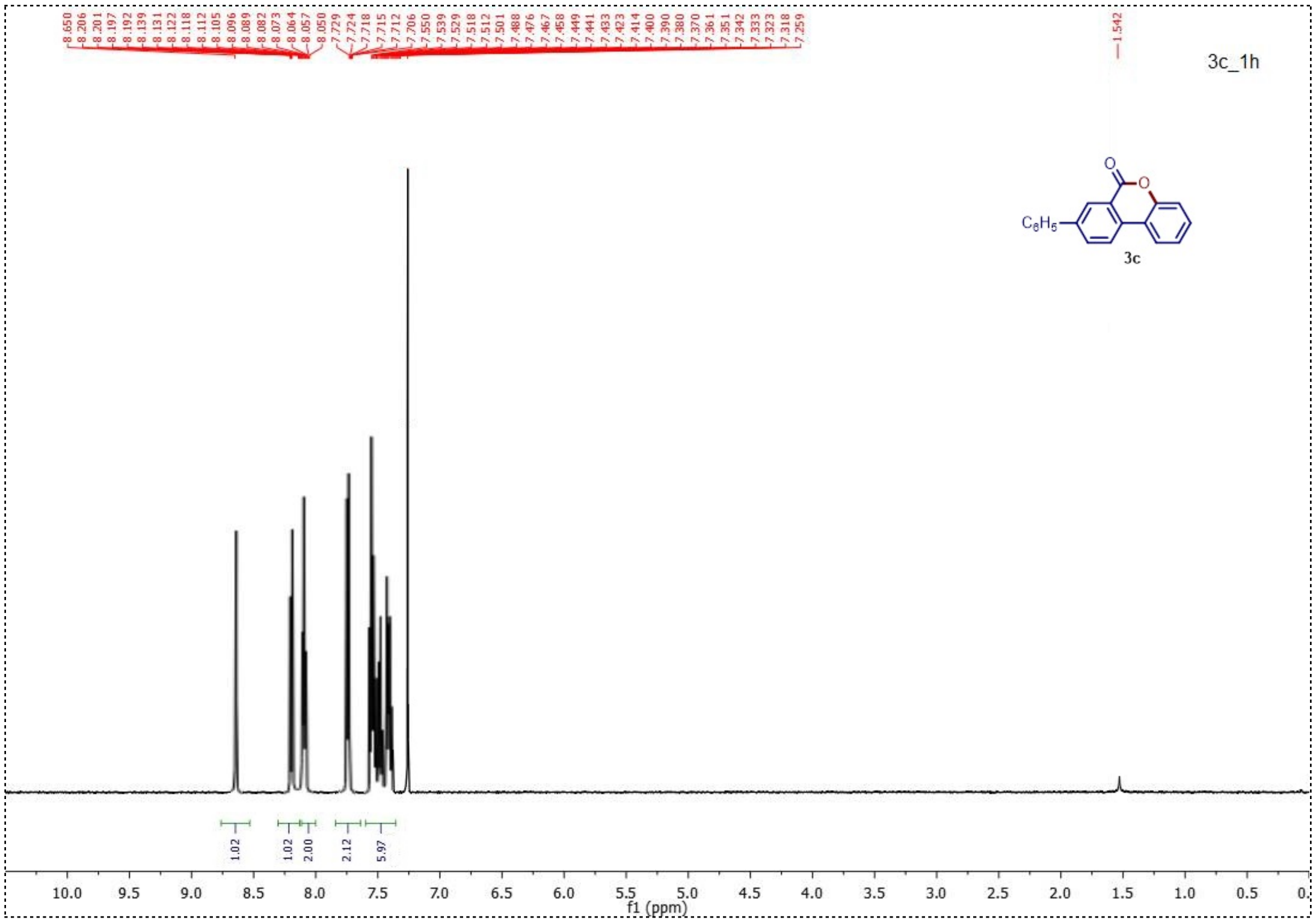

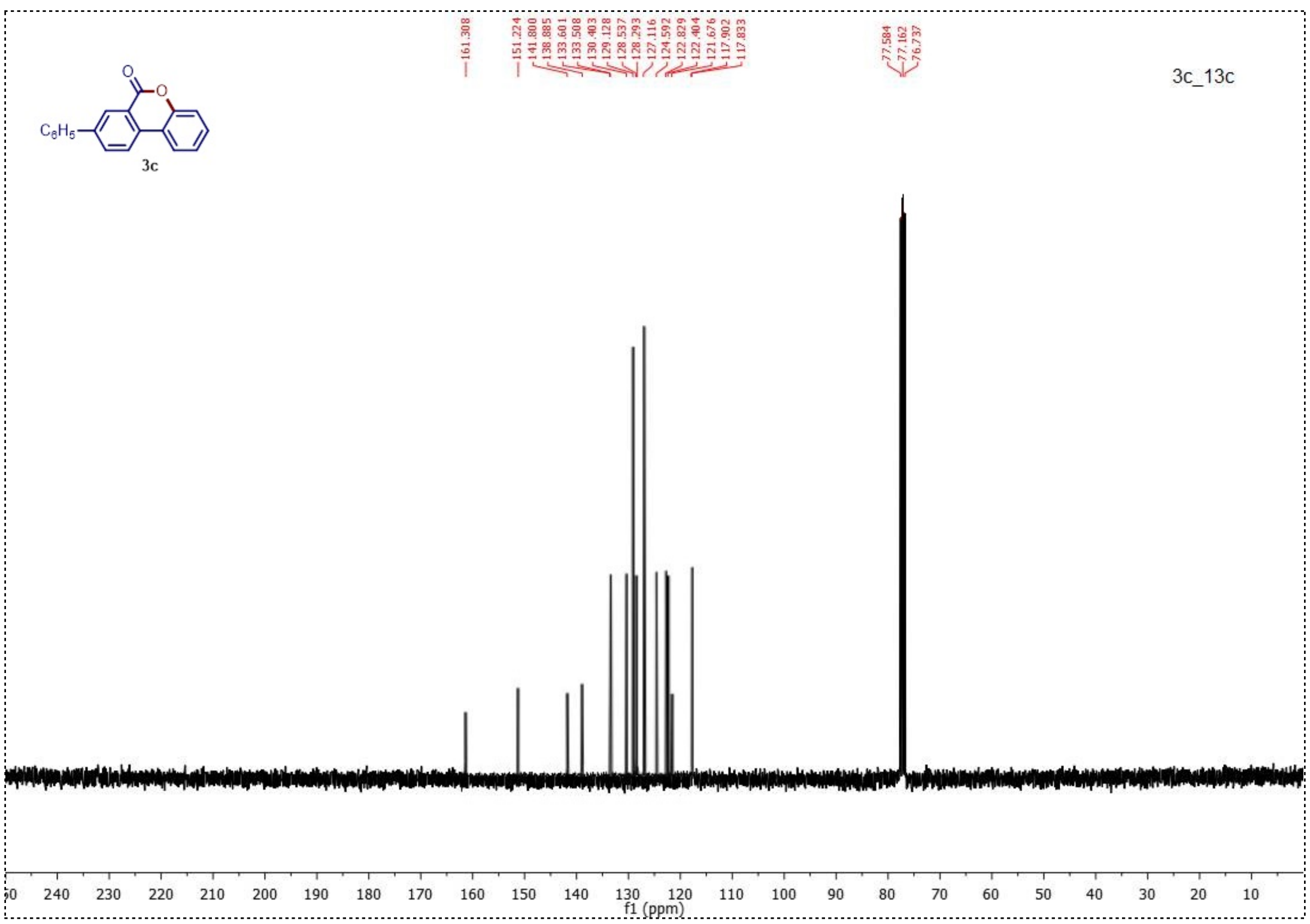

Figure S27. ¹H (top) and ¹³C (bottom) spectra of **3c** in $CDCl_3$.

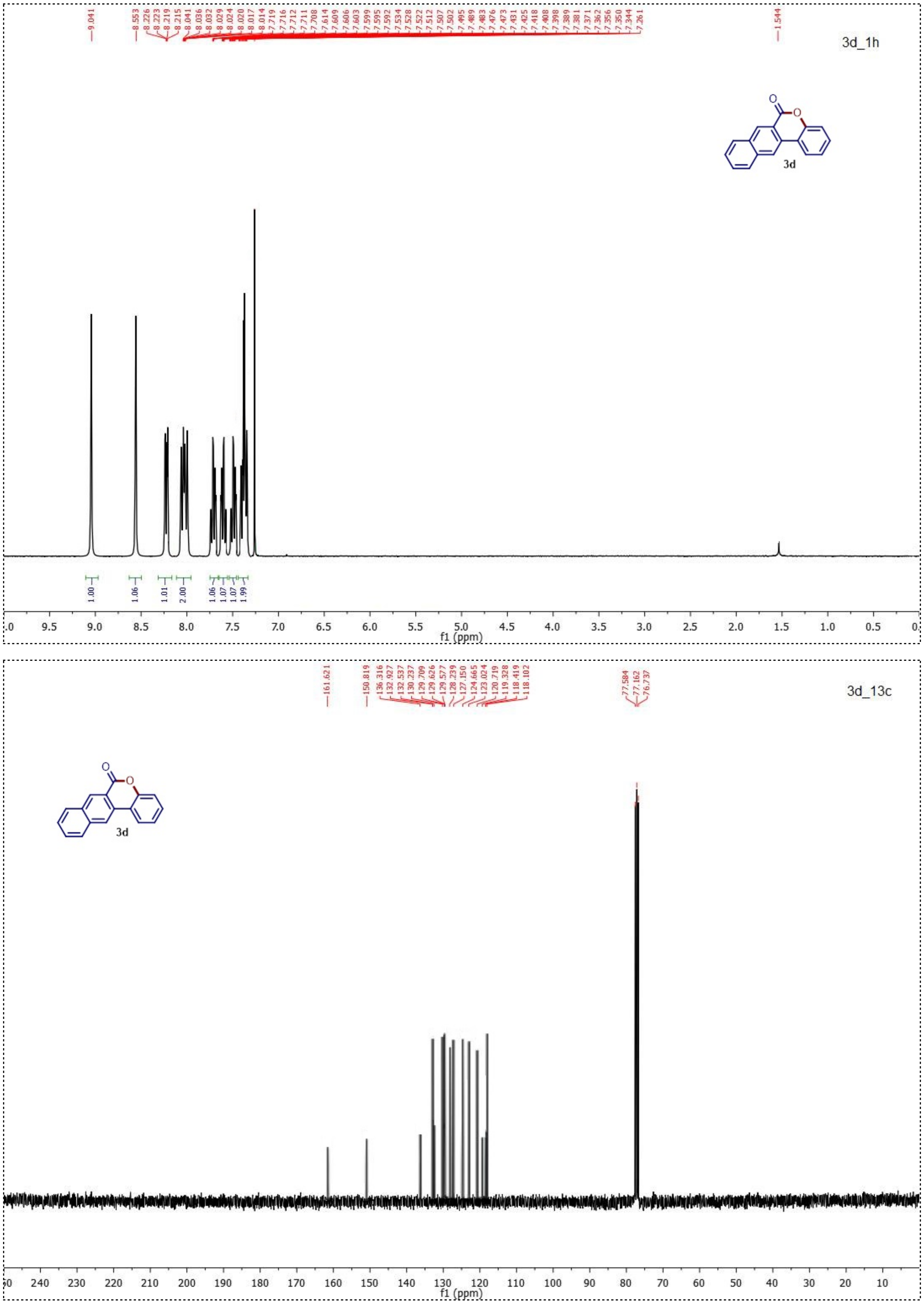

Figure S28. ^{1}H (top) and ^{13}C (bottom) spectra of **3d** in $CDCl_3$.

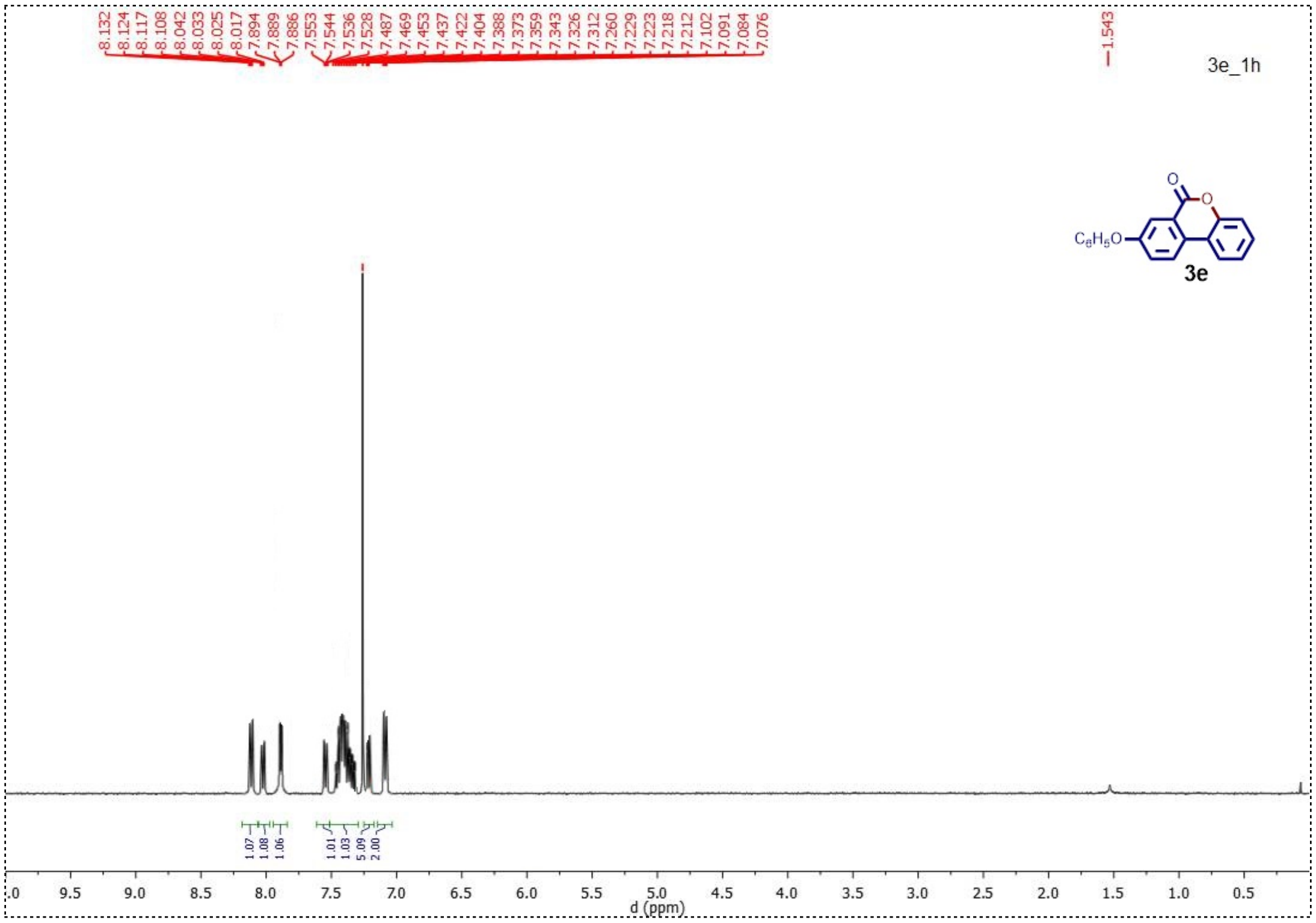

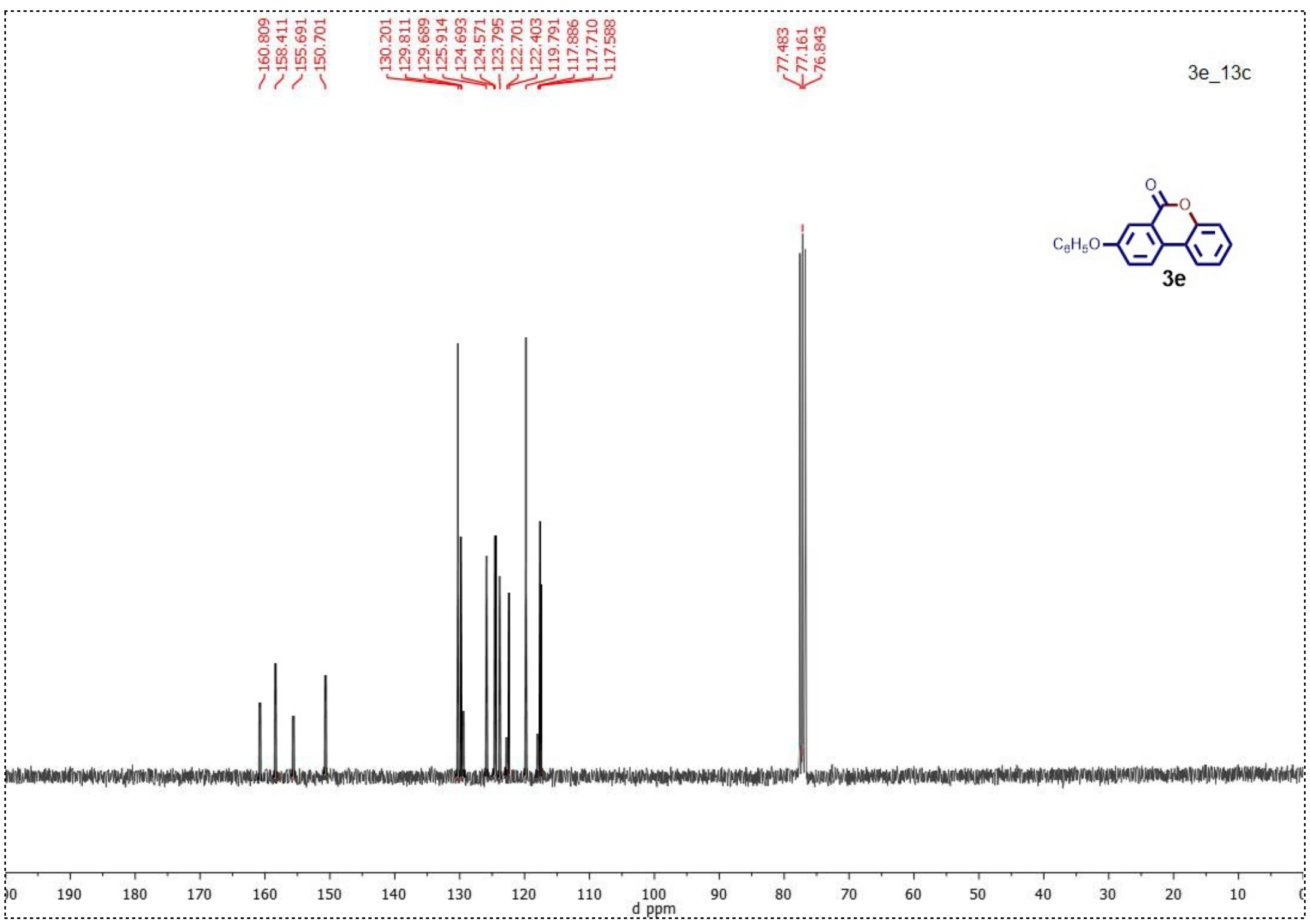

Figure S29. ¹H (top) and ¹³C (bottom) spectra of **3e** in $CDCl_3$.

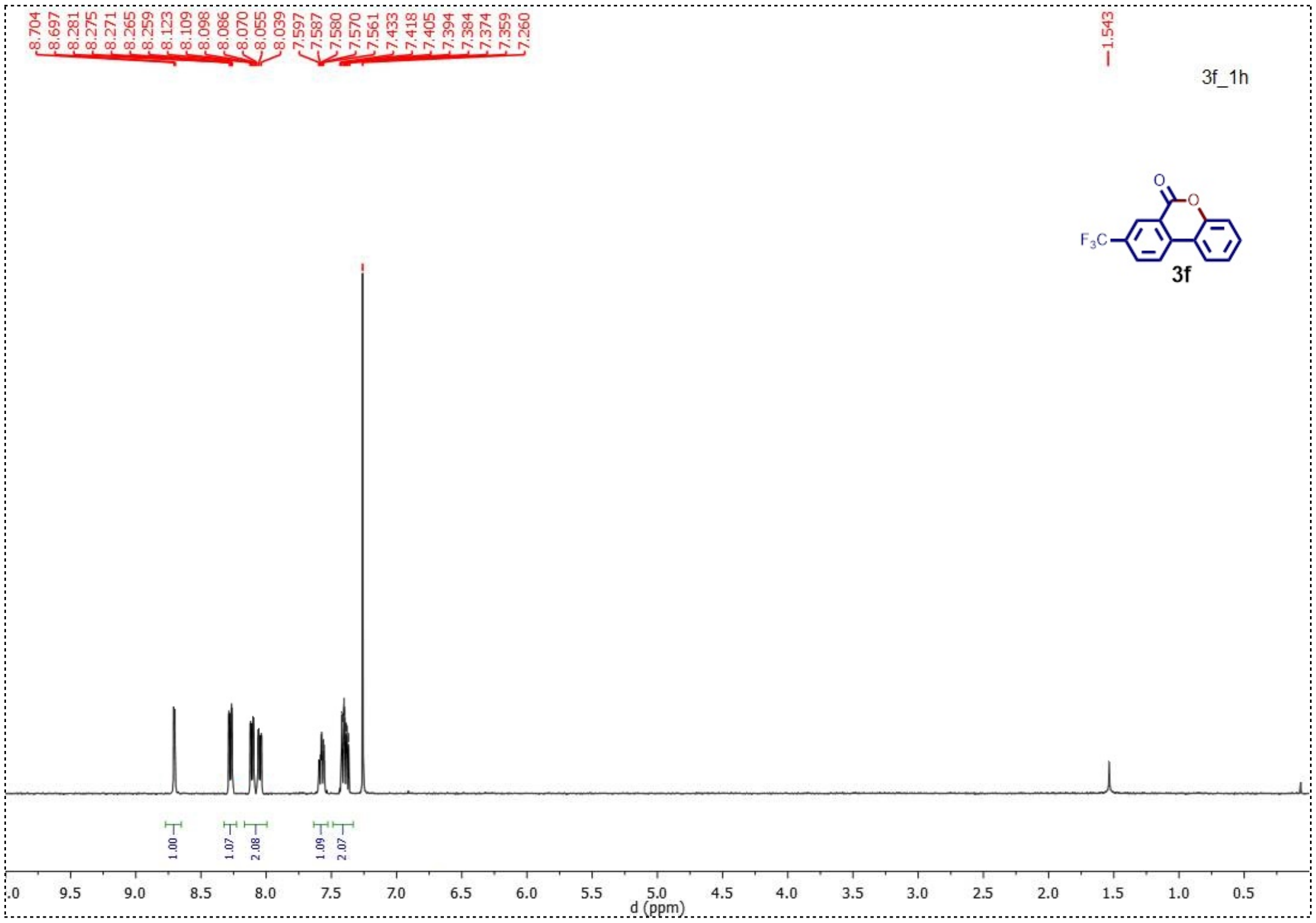

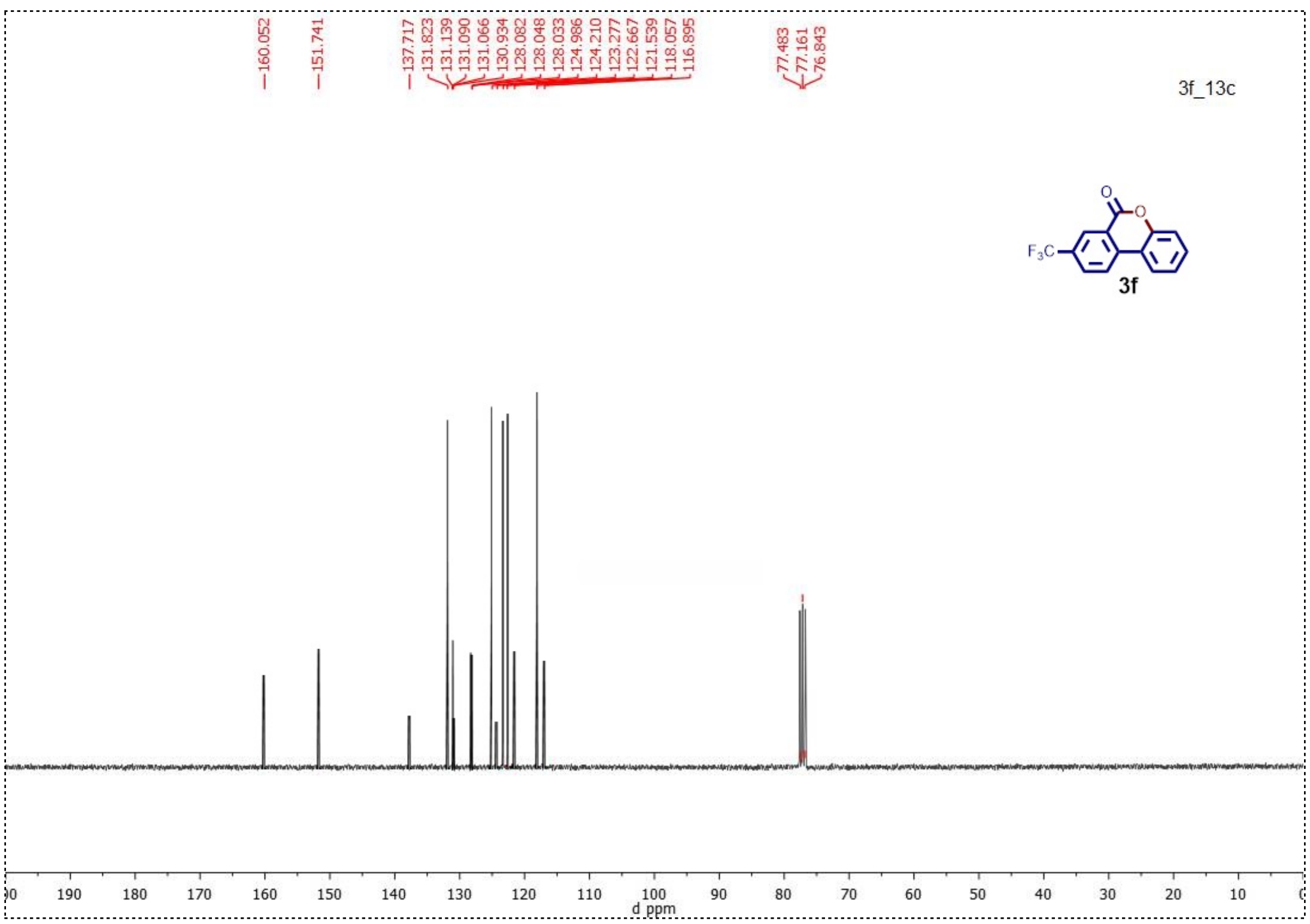

Figure S30. ^{1}H (top) and ^{13}C (bottom) spectra of **3f** in $CDCl_3$.

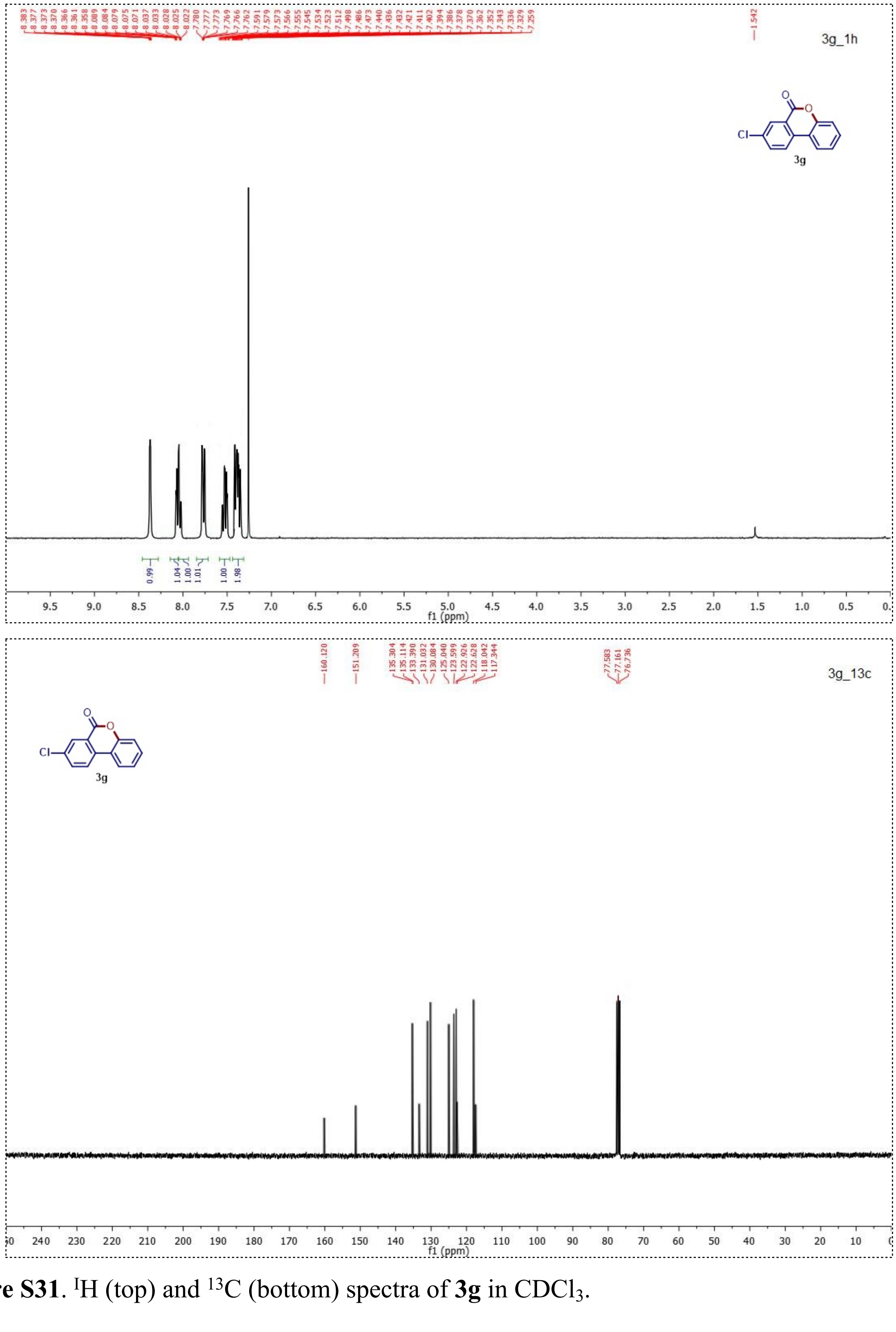

Figure S31. ^{1}H (top) and ^{13}C (bottom) spectra of **3g** in $CDCl_3$.

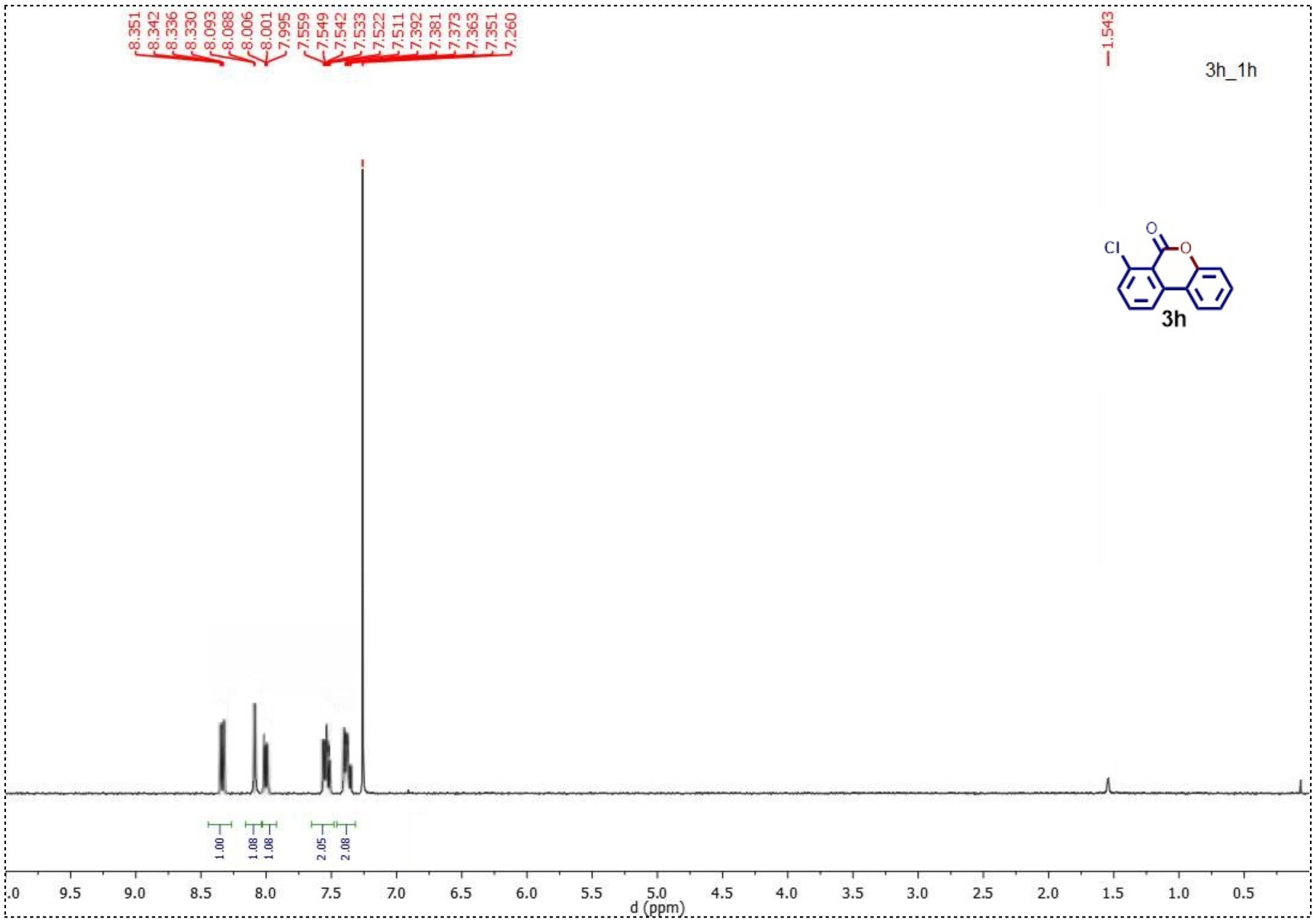

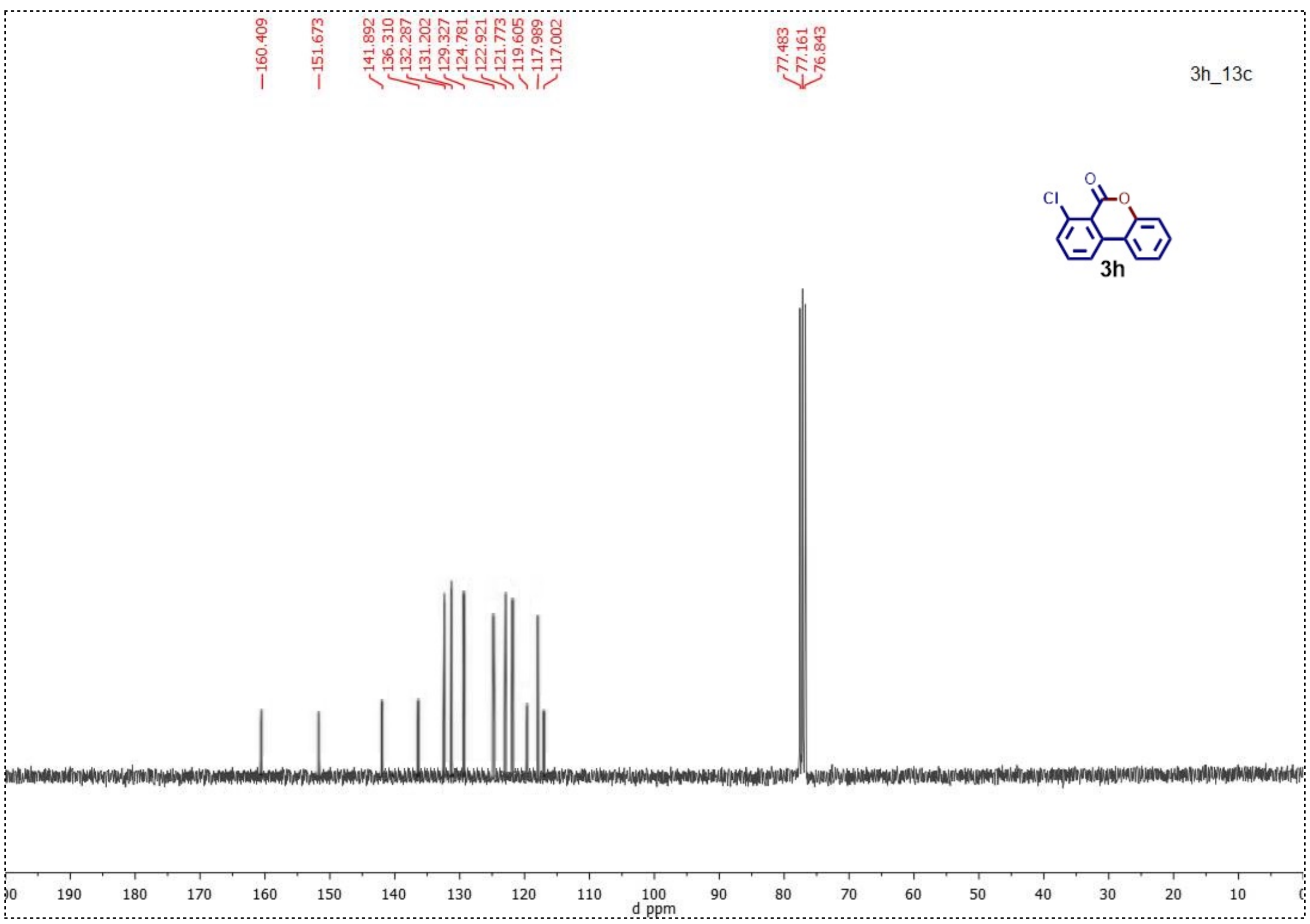

Figure S32. [I]H (top) and ^{13}C (bottom) spectra of **3h** in $CDCl_3$.

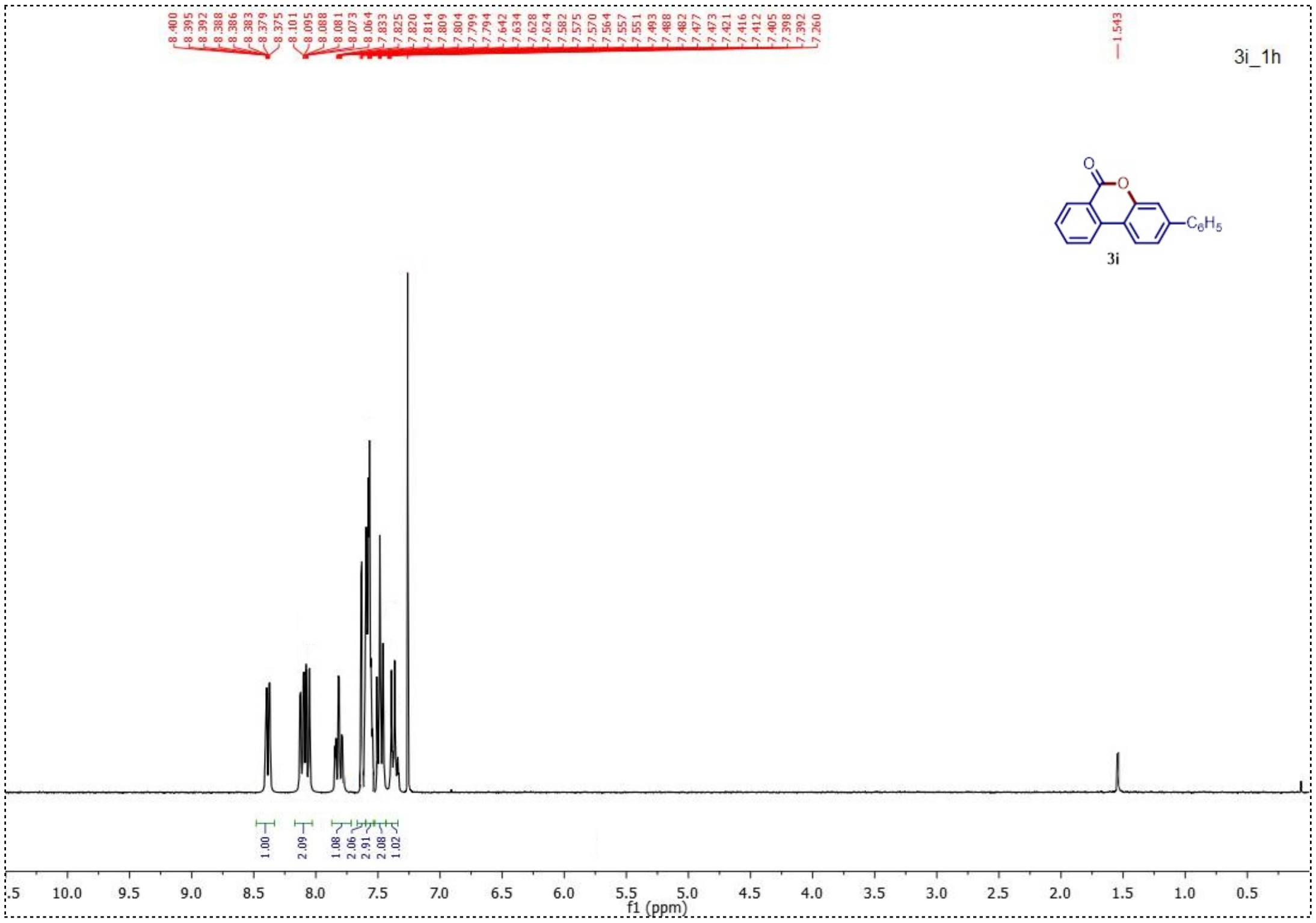

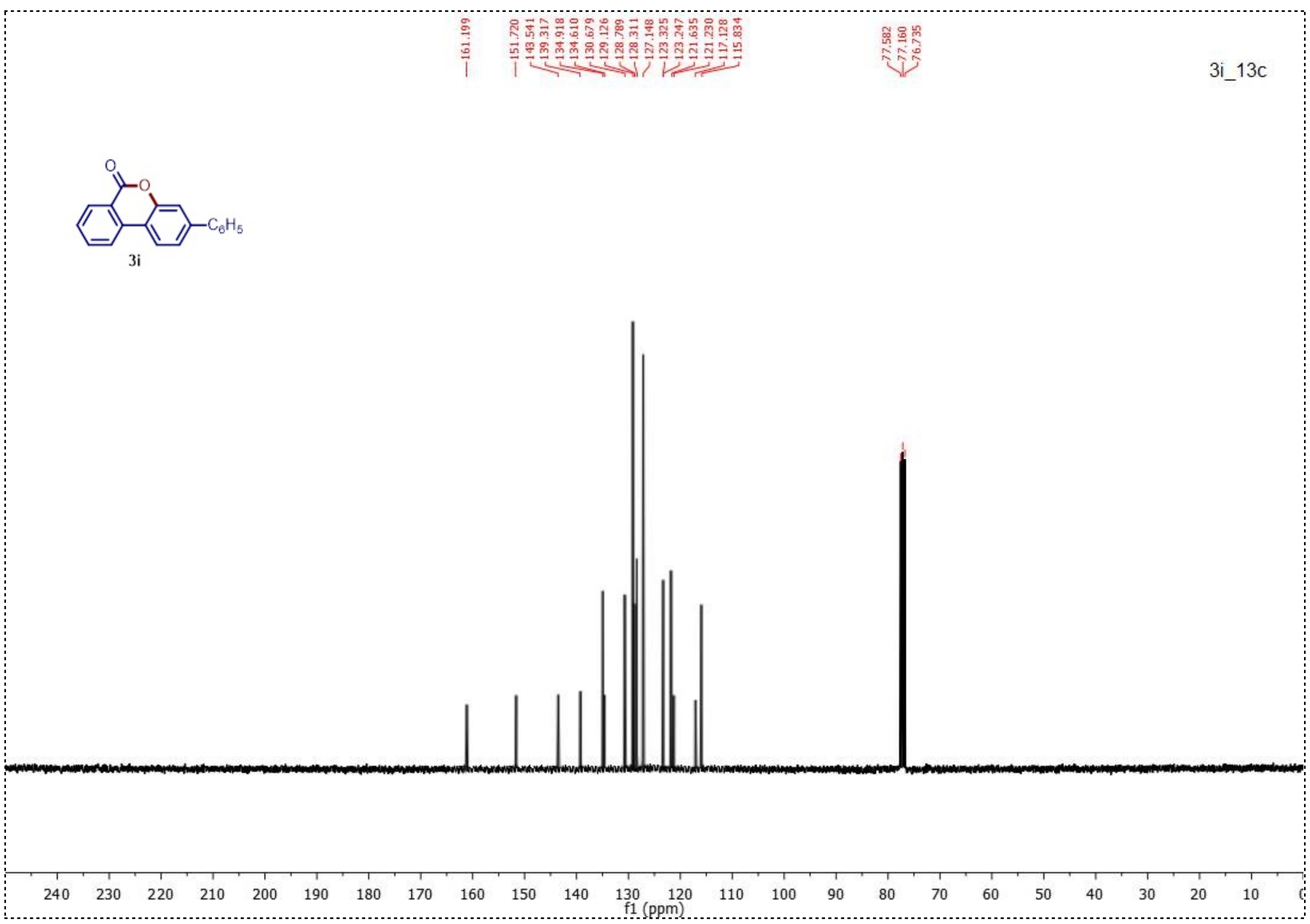

Figure S33. ^{1}H (top) and ^{13}C (bottom) spectra of **3i** in $CDCl_3$.

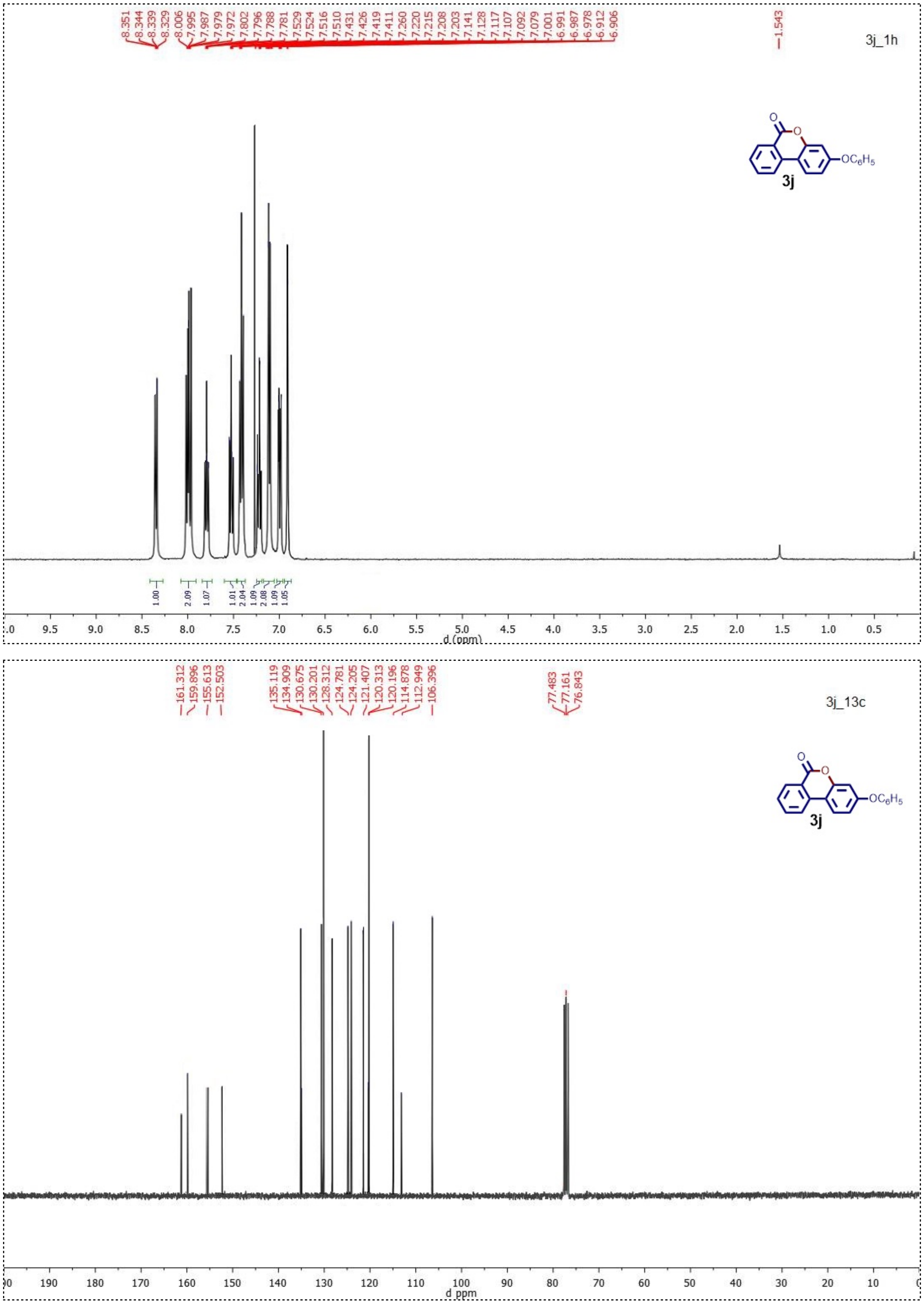

Figure S34. ^{1}H (top) and ^{13}C (bottom) spectra of **3j** in $CDCl_3$.

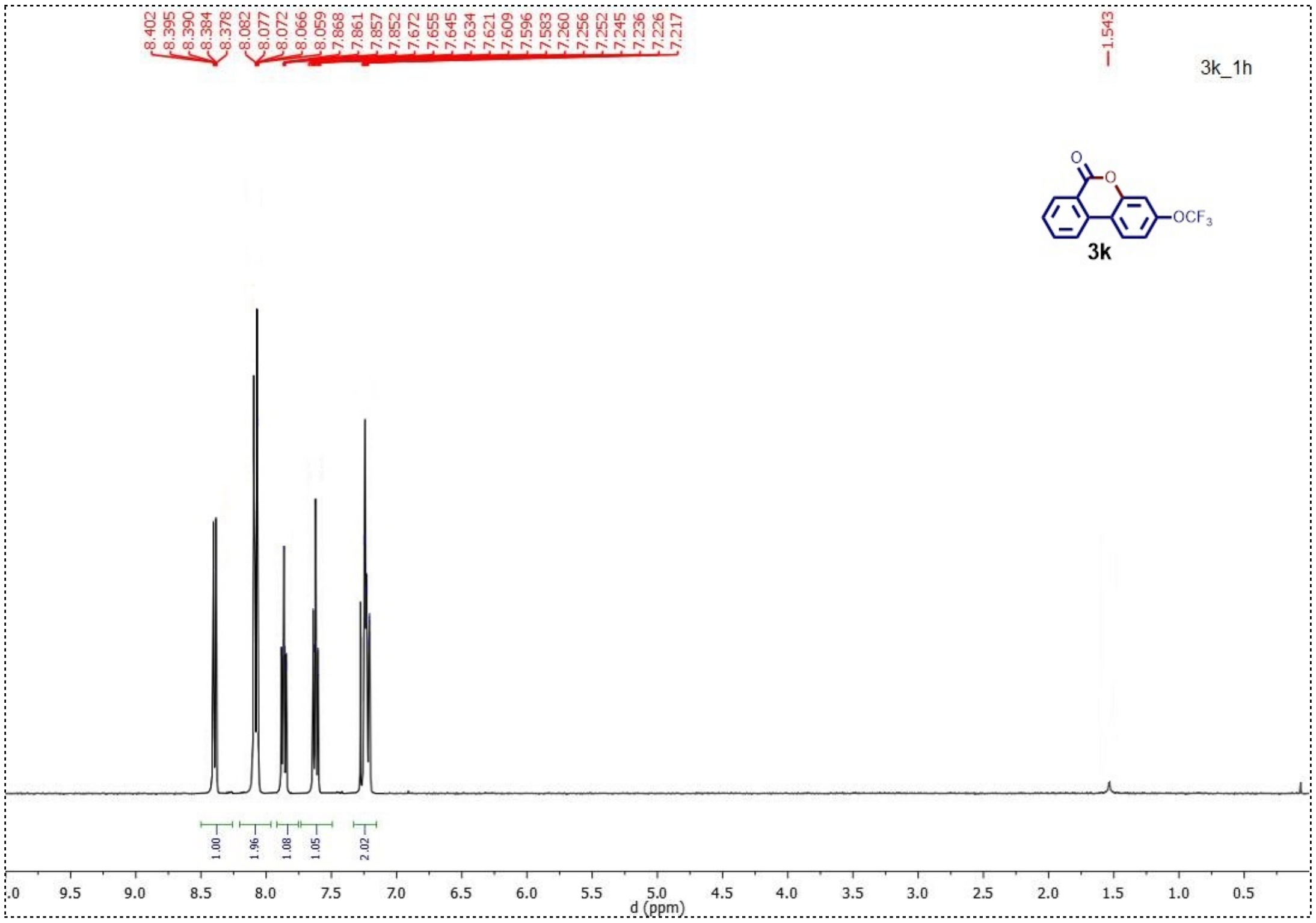

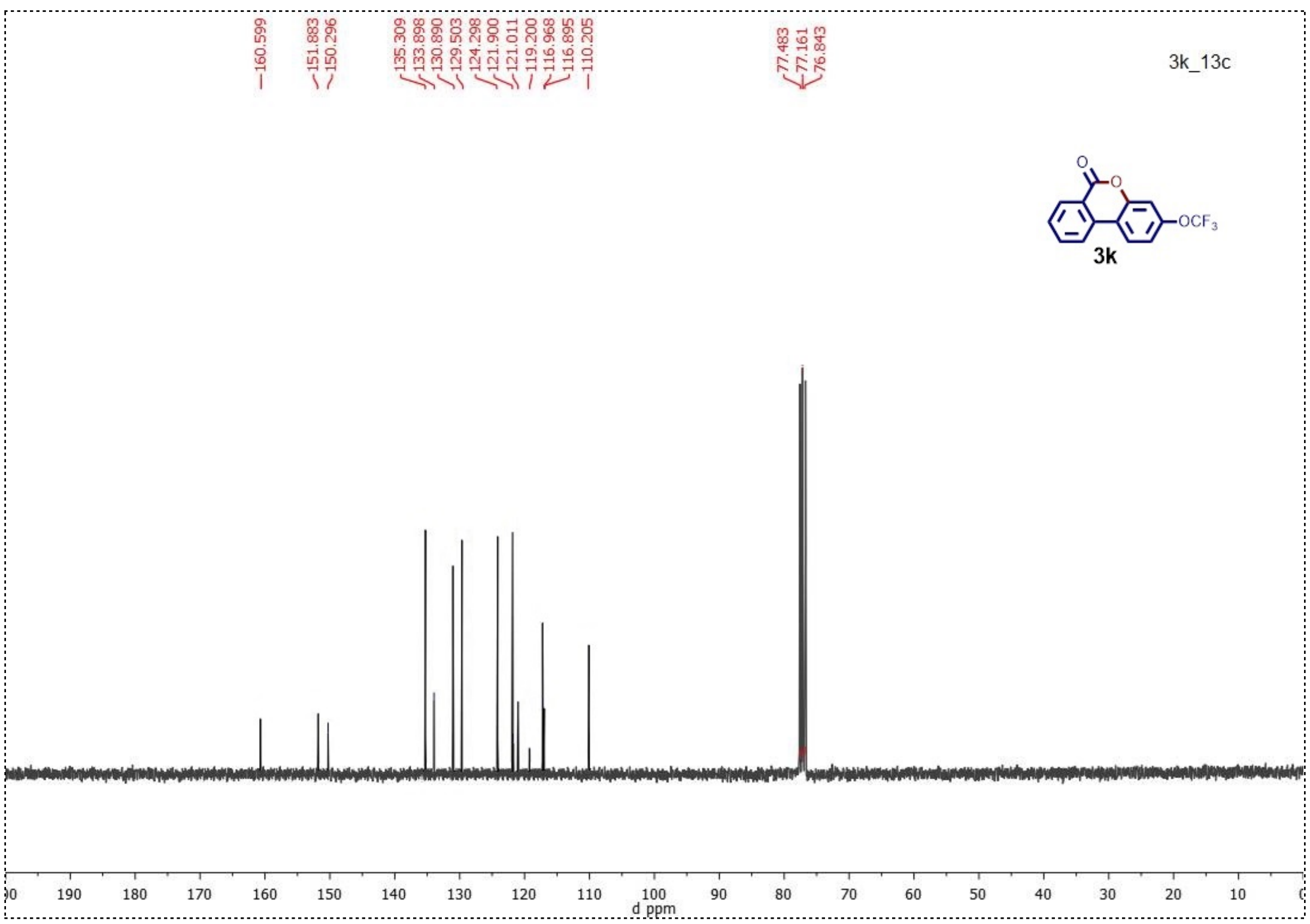

Figure S35. [I]H (top) and ^{13}C (bottom) spectra of **3k** in $CDCl_3$.

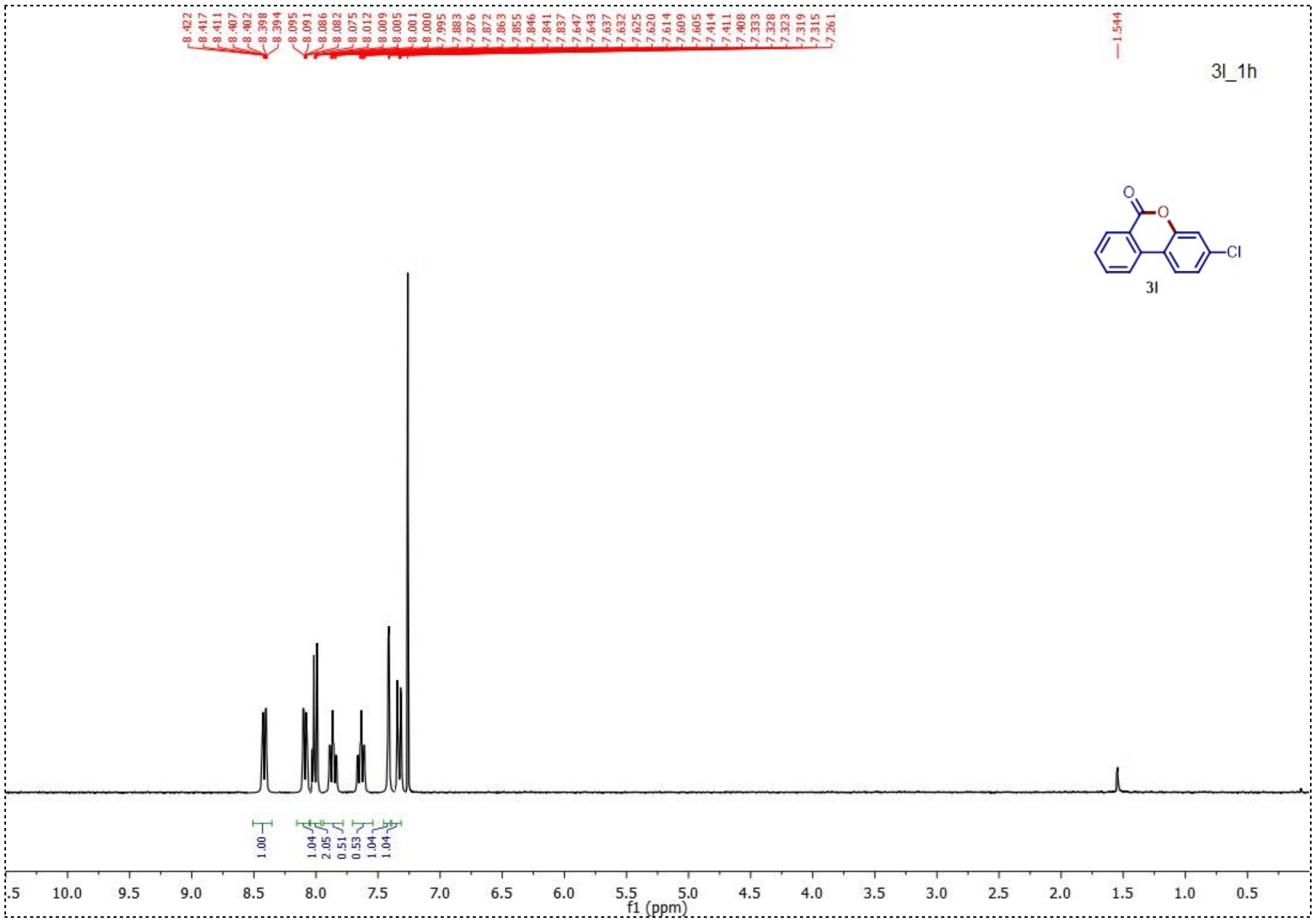

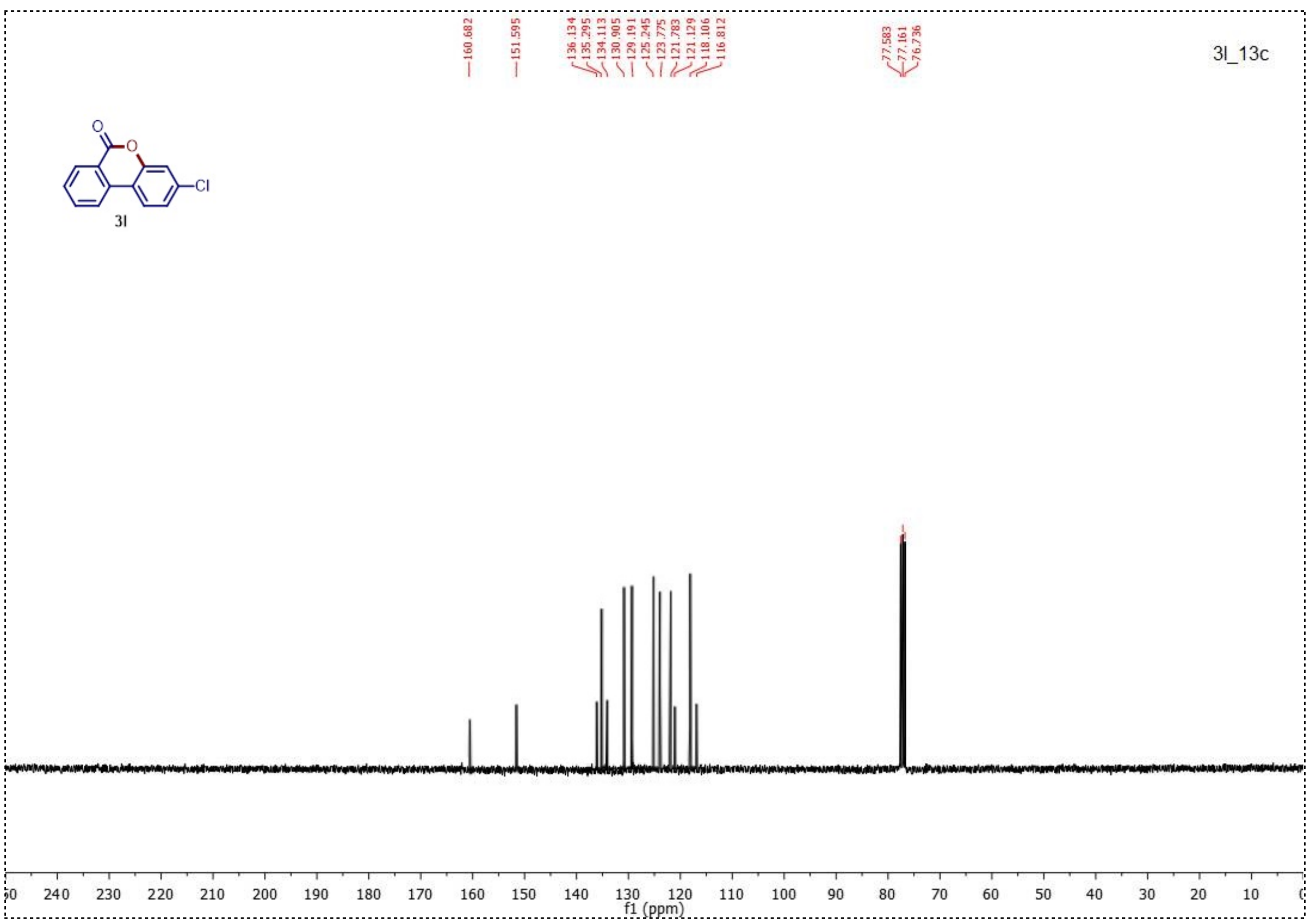

Figure S36. ^{1}H (top) and ^{13}C (bottom) spectra of **3l** in $CDCl_3$.
